# The trans-Golgi-localized protein BICAT3 regulates manganese allocation and matrix polysaccharide biosynthesis

**DOI:** 10.1093/plphys/kiac387

**Published:** 2022-08-22

**Authors:** Jie He, Bo Yang, Gerd Hause, Nico Rössner, Tina Peiter-Volk, Martin H Schattat, Cătălin Voiniciuc, Edgar Peiter

**Affiliations:** Plant Nutrition Laboratory, Institute of Agricultural and Nutritional Sciences, Faculty of Natural Sciences III, Martin Luther University Halle-Wittenberg, Halle (Saale), 06120, Germany; Independent Junior Research Group—Designer Glycans, Leibniz Institute of Plant Biochemistry, Halle (Saale), 06120, Germany; Biocentre, Martin Luther University Halle-Wittenberg, Halle (Saale), 06120, Germany; Plant Nutrition Laboratory, Institute of Agricultural and Nutritional Sciences, Faculty of Natural Sciences III, Martin Luther University Halle-Wittenberg, Halle (Saale), 06120, Germany; Plant Nutrition Laboratory, Institute of Agricultural and Nutritional Sciences, Faculty of Natural Sciences III, Martin Luther University Halle-Wittenberg, Halle (Saale), 06120, Germany; Plant Physiology, Institute of Biology, Faculty of Natural Sciences I, Martin Luther University Halle-Wittenberg, Halle (Saale), 06120, Germany; Independent Junior Research Group—Designer Glycans, Leibniz Institute of Plant Biochemistry, Halle (Saale), 06120, Germany; Horticultural Sciences Department, University of Florida, Gainesville, Florida 32611, USA; Plant Nutrition Laboratory, Institute of Agricultural and Nutritional Sciences, Faculty of Natural Sciences III, Martin Luther University Halle-Wittenberg, Halle (Saale), 06120, Germany

## Abstract

Manganese (Mn^2+^) is essential for a diversity of processes, including photosynthetic water splitting and the transfer of glycosyl moieties. Various Golgi-localized glycosyltransferases that mediate cell wall matrix polysaccharide biosynthesis are Mn^2+^ dependent, but the supply of these enzymes with Mn^2+^ is not well understood. Here, we show that the BIVALENT CATION TRANSPORTER 3 (BICAT3) localizes specifically to trans-cisternae of the Golgi. In agreement with a role in Mn^2+^ and Ca^2+^ homeostasis, BICAT3 rescued yeast (*Saccharomyces cerevisiae*) mutants defective in their translocation. Arabidopsis (*Arabidopsis thaliana*) knockout mutants of *BICAT3* were sensitive to low Mn^2+^ and high Ca^2+^ availability and showed altered accumulation of these cations. Despite reduced cell expansion and leaf size in Mn^2+^-deficient *bicat3* mutants, their photosynthesis was improved, accompanied by an increased Mn content of chloroplasts. Growth defects of *bicat3* corresponded with an impaired glycosidic composition of matrix polysaccharides synthesized in the trans-Golgi. In addition to the vegetative growth defects, pollen tube growth of *bicat3* was heterogeneously aberrant. This was associated with a severely reduced and similarly heterogeneous pectin deposition and caused diminished seed set and silique length. Double mutant analyses demonstrated that the physiological relevance of BICAT3 is distinct from that of ER-TYPE CA^2+^-ATPASE 3, a Golgi-localized Mn^2+^/Ca^2+^-ATPase. Collectively, BICAT3 is a principal Mn^2+^ transporter in the trans-Golgi whose activity is critical for specific glycosylation reactions in this organelle and for the allocation of Mn^2+^ between Golgi apparatus and chloroplasts.

## Teaser

Activity of BIVALENT CATION TRANSPORTER 3 in the trans-Golgi is critical for glycosylation reactions, plant growth under Mn deficiency, pollen tube elongation, and intracellular distribution of Mn.

## Introduction

Manganese (Mn^2+^) is an essential trace metal for plants, which functions as a cofactor of enzymes or a catalyst in metal clusters (Andresen et al., 2018; Alejandro et al., 2020). One of the most prominent roles of Mn^2+^ is to constitute the Mn_4_O_5_Ca catalytic cluster in the oxygen-evolving complex (OEC) of photosystem II (PSII) for water oxidation, and consequently the supply of electrons and protons for the photosynthetic electron chain and ATP production. Furthermore, THYLAKOID-ASSOCIATED PHOSPHATASE 38, which is critical for state transition between PSI and PSII, requires a binuclear Mn or Mg center (Wei et al., 2015). Besides its roles in photosynthetic activities, Mn^2+^ is also involved in ROS scavenging as a component of mitochondria- and peroxisome-localized manganese superoxide dismutase (He et al., 2021). Moreover, Mn^2+^ is essential for the catalytic activity of many Golgi-localized glycosyltransferases (GTs), which are critical for the glycosylation of proteins and lipids, and for the synthesis of complex cell wall matrix polysaccharides (He et al., 2021). Matrix polymers (hemicelluloses and pectins) account for two-thirds of the Arabidopsis (*Arabidopsis thaliana*) primary cell wall. They contribute substantially to the mechanical properties of the wall and are thus critical for plant growth and development (Cosgrove, 2016, 2018; Rui and Dinneny, 2020; Molina et al., 2021). Matrix polysaccharides influence the organization of cellulose microfibrils, the main load-bearing components of the cell wall, to modulate wall extensibility and organ formation (Park and Cosgrove, 2012; Rui and Anderson, 2016; Zhao et al., 2019). Golgi-localized xyloglucan xylosyltransferases (XXT1 and 2), which are involved in xyloglucan biosynthesis, are Mn^2+^-dependent (Culbertson et al., 2016, 2018). The *xxt1xxt2* double mutant is smaller than the wild-type in shoot size, with a diminished amount and abnormal composition of xyloglucans. Biosynthesis of cell wall sugars may be mutually affected, as *xxt1xxt2* not only displays a defect in xyloglucan production, but also in cellulose production and pectin methylation at the shoot apical meristem (Zhao et al., 2019). Another class of matrix sugars, pectins, plays pivotal roles in a plethora of biological processes, including cell adhesion and morphogenesis (Bouton et al., 2002; Altartouri et al., 2019; Haas et al., 2020), organ formation (Peaucelle et al., 2011), plant growth (Kim et al., 2015), pollen tube growth (Kim et al., 2015; Lund et al., 2020), fertilization (Duan et al., 2020), stomatal functions (Amsbury et al., 2016; Rui et al., 2019), and also confers resistance to biotic and tolerance to abiotic stress (Bacete et al., 2018; Wu et al., 2018; De Lorenzo et al., 2019).

In addition to matrix sugars, the *N*- and *O*-glycosylation of proteins and lipids are essential for plant development and stress responses (Kobayashi, 2016; Nagashima et al., 2018; Huby et al., 2020; Mortimer and Scheller, 2020; Seifert, 2020; Silva et al., 2020). Arabinogalactan proteins (AGPs) have been proposed to represent Ca^2+^ capacitors that reversibly bind and release apoplastic Ca^2+^ in a pH-dependent manner by their β-linked glucuronic acid (GlcA) residues and which thereby determine numerous biological processes, such as pollen tube growth and guidance, seedling growth, and Ca^2+^ oscillations in roots (Lamport et al., 2018; Lopez-Hernandez et al., 2020). Accordingly, mutants of Mn^2+^-dependent hydroxyproline galactosyltransferases (GALT2-6) that are involved in AGP biosynthesis, show defects in root, leaf, and pollen tube growth (Basu et al., 2015a, 2015b). Mn^2+^-dependent transfer of GlcA residues to glycosyl inositol phosphoryl ceramide sphingolipids by inositol phosphoryl ceramide glucuronosyltransferase 1 (IPUT1/PGSIP6/MOCA1) is also crucial for generative and vegetative development, and the glycosylated sphingolipids are believed to act as Na^+^ sensors (He et al., 2021). Altogether, an adequate Mn^2+^ supply for Golgi-localized glycosyl transfer processes appears to be crucial for most aspects of plant performance. However, as this assumption is derived from in vitro analyses of individual enzyme activities, the mechanism and bottlenecks for Mn^2+^ supply *in planta* remain poorly understood.

In Arabidopsis, Mn^2+^ is acquired by the high-affinity transporter NRAMP1 and may also enter the root in high amounts via the Fe uptake transporter IRT1. The pathway of xylem loading is dubious in dicots (Alejandro et al., 2020). Intracellularly, Mn^2+^ may be sequestrated in vacuoles by CATION DIFFUSION FACILITATOR/METAL TOLERANCE PROTEIN (CDF/MTP) and CATION/H^+^ EXCHANGER (CAX) transporters, which is an important mechanism of its detoxification and its storage in seeds (Eroglu et al., 2016, 2017; He et al., 2021; Höller et al., 2022). Transfer of Mn^2+^ into the endoplasmic reticulum by the P_2A_-type ATPase ER-TYPE CA^2+^-ATPASE 1 (ECA1) also confers tolerance to the metal (Liang et al., 1997). To fulfill its function in the OEC, Mn^2+^ needs to cross the chloroplast envelope and the thylakoid membrane. Both steps are mediated by members of the BIVALENT CATION TRANSPORTER (BICAT) family, with BICAT1/PAM71/CCHA1 supplying the thylakoid lumen and BICAT2/CMT1 operating in the inner envelope (Schneider et al., 2016; Eisenhut et al., 2018; Zhang et al., 2018). Mn^2+^ supply of the chloroplast is also dependent on the NRAMP2 transporter in the trans-Golgi network (Alejandro et al., 2017). This transporter is functionally epistatic to the vacuolar Mn^2+^ exporters NRAMP3 and NRAMP4, pointing to a crucial function of vesicular compartments for intra-organellar Mn^2+^ distribution (He et al., 2021). This notion is also supported by the severely aberrant Mn^2+^ handling in mutants of *MTP11*, that are very hypersensitive to Mn^2+^ excess and hypertolerant to Mn^2+^ limitation. MTP11 has been localized to the prevacuolar compartment and the Golgi, and, based on lower Mn^2+^ levels in the mutants, suggested to promote Mn^2+^ detoxification by vesicular secretion (Delhaize et al., 2007; Peiter et al., 2007). Another Mn^2+^ translocator, the P_2A_-type ATPase ECA3, has similarly been localized to the Golgi, and *eca3* mutants are equally sensitive to Mn^2+^ toxicity (Mills et al., 2008).

In yeast (*Saccharomyces cerevisiae*) and humans (*Homo sapiens*), Mn^2+^ loading of Golgi vesicles is pursued by SPCA and SERCA-type ATPases and transporters related to the above-mentioned BICAT proteins operating side-by-side (He et al., 2021). Those transporters, GDT1 in yeast and TMEM165 in humans, permeate Ca^2+^ besides Mn^2+^ (Demaegd et al., 2013; Thines et al., 2018; Stribny et al., 2020), as was also shown for BICAT1 and BICAT2 (Frank et al., 2019). The absence of TMEM165 inflicts defects in protein glycosylation by limiting the activity of Mn^2+^-dependent β-1,4-galactosyltransferase 1 (B4GALT1), which co-localizes with TMEM165 in the trans-Golgi (Foulquier et al., 2012; Foulquier and Legrand, 2020).

In plants, not only protein glycosylation demands Mn^2+^ as a co-factor, but also the biosynthesis of matrix polysaccharides, mediated by a plethora of glycosyl transferases (He et al., 2021). It has been known for long that those reactions are spatially organized to form an efficient assembly line in the secretory pathway. Specific Mn^2+^ transporters may thus be required for discrete glycosylation steps, as it is the case in animals. In plants, the specific role of Golgi-localized Mn^2+^ transporters and the functional interaction of their activity with Mn^2+^-requiring processes in other organelles are poorly understood despite some recent progress (Yang et al., 2021; Zhang et al., 2021). Here, we demonstrate that a protein of the BICAT family that has been suggested to function as Golgi-localized Mn^2+^ transporter is required for the formation of specific glycosyl linkages, rendering it essential under numerous vegetative and generative circumstances. Importantly, BICAT3 affects Mn^2+^ accumulation as well as its subcellular distribution between Golgi and chloroplast. Double mutant analyses show that its physiological relevance is distinct from that of Ca^2+^/Mn^2+^-ATPase ECA3.

## Results

### BICAT3 primarily localizes to the trans-Golgi and is ubiquitously expressed

**Figure 1:**
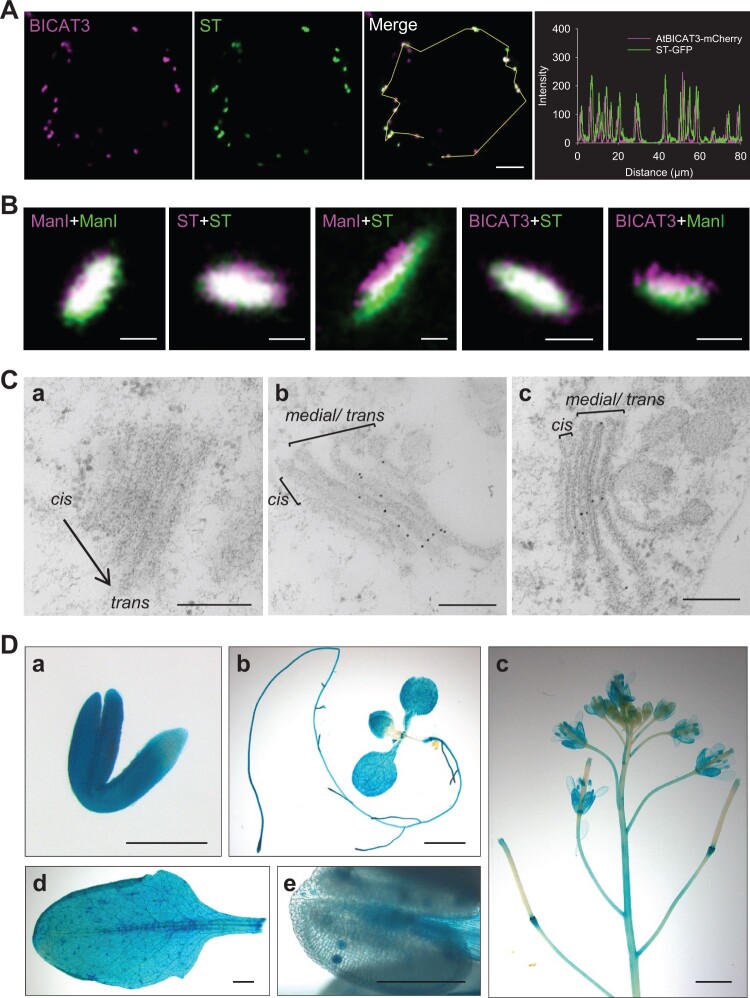
BICAT3 localizes to the trans*-*Golgi and is ubiquitously expressed in Arabidopsis. A, Subcellular localization of BICAT3 in Arabidopsis mesophyll protoplasts. *BICAT3* was co-expressed with a trans-Golgi marker (ST). Bar represents 5 µm. B, High-resolution co-localization of BICAT3, a cis-Golgi marker (ManI), and a trans-Golgi marker (ST) in *N. benthamiana* mesophyll cells. Magenta letters indicate proteins tagged with mCherry, green letters indicate proteins tagged with GFP. Bars represent 500 nm. C, Ultrastructural localization of BICAT3-Venus by immunogold staining. a, Wild-type Golgi apparatus as a negative control. b and c, Localization of BICAT3-Venus in the Golgi apparatus of *bicat3-1* leaf cells expressing *BICAT3-Venus* under control of the native *BICAT3* promoter. Scale bars represent 200 nm. D, Expression of *BICAT3* at different growth stages as determined by GUS staining of a *ProBICAT3-GUS* line. a, Germinated seed. b, 10-day-old seedling. c, Inflorescence and siliques. d, Mature rosette leaf. e, Anther. Scale bars represent 300 µm (a), 200 µm (e) or 2 mm (b, c, and d).

The genome of *A. thaliana* harbors five members of the functionally conserved UPF0016 family, BICAT 1–5. Previous phylogenetic analyses revealed that BICAT3 is the closest Arabidopsis homolog to human TMEM165 and yeast GDT1 (Demaegd et al., 2014). Both proteins are Ca^2+^/Mn^2+^ transporters localized in the Golgi and critical for Mn^2+^ supply for protein glycosylation (Foulquier et al., 2012; Colinet et al., 2016; Potelle et al., 2016; Dulary et al., 2018; Thines et al., 2019). To investigate the subcellular localization of BICAT3, we co-expressed *BICAT3* with organelle markers fused with different fluorescent proteins in Arabidopsis mesophyll protoplasts. Complementation of the *bicat3-1* mutant with *BICAT3-Venus* driven by the native *BICAT3* promoter confirmed the functionality of the fusion construct (Supplemental Figure S1). In confocal laser scanning microscopy (CLSM) analyses, BICAT3 co-localized with the trans*-*Golgi marker sialyl transferase (ST; Wee et al., 1998) (Figure 1A), but not with markers targeted to mitochondria or peroxisomes (Supplemental Figure S2). Since conventional CLSM cannot reliably discriminate Golgi subcompartments, we recorded images at high speed and resolution with a STELLARIS 8 microscope (Leica, Wetzlar, Germany). This enabled a clear discrimination of fluorescent proteins targeted to the *cis/medial*-Golgi [α-mannosidaseI (ManI); Donohoe et al. (2013)] and the trans-Golgi (ST) (Figure 1B). While BICAT3 overlapped perfectly with the trans-Golgi marker, a shift was noted to that for the cis-Golgi, indicating that BICAT3 is not a cis-Golgi-specific protein, as previously claimed (Yang et al., 2021). Supporting the live-cell analyses, immunogold labeling with Venus antibodies in the complementation line primarily detected BICAT3 in the medial- and trans-Golgi (Figure 1C).

To reveal the expression pattern of *BICAT3* in different tissues, its promoter activity was visualized by using the β-glucuronidase (GUS) reporter. Strong *GUS* expression was detected in most of the tissues (embryo, root, leaves, stem, and flowers) throughout different growth stages (Figure 1D). The expression of *BICAT3* was not affected by imbalanced Ca^2+^ or Mn^2+^ supply (Supplemental Figure S3).

### BICAT3 acts as Ca^2+^ and Mn^2+^ transporter in yeast

**Figure 2:**
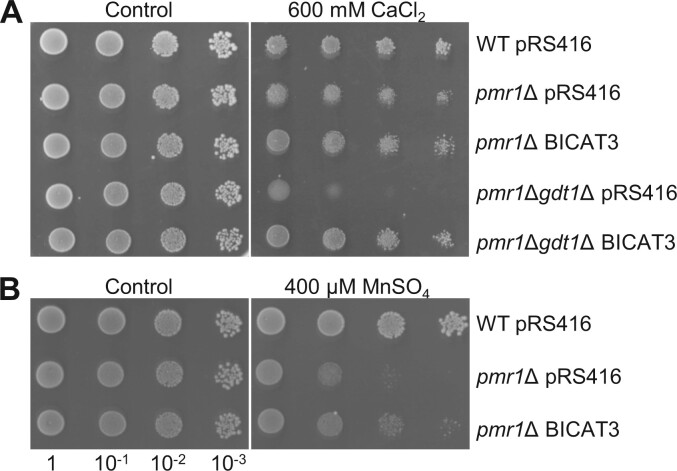
*BICAT3* complements Ca^2+^- and Mn^2+^-sensitive yeast strains. A, Growth of wild-type, *pmr1*Δ, and *pmr1*Δ*gdt1*Δ with and without *BICAT3* under Ca^2+^-toxic conditions. B, Growth of wild-type and *pmr1*Δ with and without *BICAT3* under Mn^2+^-toxic conditions. Liquid cultures of the strains were serially diluted and dropped onto media as indicated.

Putative BICAT3 orthologs in yeast and humans mediate evolutionarily conserved Ca^2+^ and Mn^2+^ transport to feed compartments of the Golgi apparatus for essential physiological processes, such as glycosylation. To test if Golgi-localized BICAT3 has similar functions in cation allocation, *BICAT3* was heterologously expressed in a *pmr1Δgdt1Δ* mutant, which displays a defect in Ca^2+^ transport into the Golgi due to the lack of the Ca^2+^ pump PMR1 and of GDT1, as well as in a *pmr1Δ* single mutant. The *pmr1Δgdt1Δ* mutant showed strong growth retardation, while *pmr1Δ* was only slightly affected by Ca^2+^ toxicity (Figure 2A) as previously reported (Demaegd et al., 2013). The Ca^2+^ sensitivity of *pmr1Δgdt1Δ* was complemented by the expression of *BICAT3*. The growth of *pmr1Δgdt1ΔBICAT3* was comparable to the *pmr1Δ* single mutant under 600-mM CaCl_2_, implying a similar ability of BICAT3 and GDT1 to transport Ca^2+^ in yeast. In addition to transport Ca^2+^, PMR1 translocates excess Mn^2+^ into the Golgi for detoxification. Heterologous expression of *BICAT3* partially complemented the Mn^2+^ sensitivity of *pmr1Δ* (Figure 2B). In contrast, BICAT3 did not complement the Zn^2+^-, Co^2+^-, Cu^2+^-, and Fe^3+^-dependent growth defects in *zrc1Δ, cot1Δ, cup2Δ*, and *ccc1Δ* yeast mutants, respectively (Supplemental Figure S4). In conclusion, the results indicate that BICAT3 acts as a Ca^2+^ and Mn^2+^ transporter in yeast.

### Growth of *bicat3* mutants is differentially affected by high and low supply of Mn^2+^ and Ca^2+^

To characterize the functions of BICAT3 in Arabidopsis, two T-DNA insertional mutants were obtained. *bicat3-1* harbors a T-DNA in the sixth intron, and *bicat3-2* carries a T-DNA insertion in the three prime untranslated region (3′-UTR) (Supplemental Figure S5). While *bicat3-1* is a knockout mutant, *bicat3-2* shows 6% of *BICAT3* transcript level compared to the wild-type. Both mutants show no obvious vegetative growth differences compared to the wild-type at mature stage under nonstressed conditions.

To assess the relevance of BICAT3 in Ca^2+^ and Mn^2+^ dependence of Arabidopsis, we cultured *bicat3-1* and wild-type seedlings on plates with different Mn^2+^ and Ca^2+^ supply. Intriguingly, young seedlings of *bicat3-1* grew more vigorously than the wild-type under control conditions (Supplemental Figures S6–S8), but were overly sensitive to Mn^2+^ deficiency (0 mM; Supplemental Figure S6) and Ca^2+^ toxicity (50 mM; Supplemental Figure S7). Conversely, *bicat3-1* seedlings were more tolerant of Mn^2+^ toxicity (1 mM; Supplemental Figure S8).

To examine the role of BICAT3 in older plants and a more controlled medium composition, *bicat3-1* and wild-type were cultured in hydroponics with different Mn^2+^ and Ca^2+^ supply (Supplemental Figure S9). With surplus Mn^2+^ (350 µM) or deficient Ca^2+^ (0.05 mM) supply, shoot and root growth of *bicat3-1* were enhanced compared to the wild-type. In contrast, shoot growth of *bicat3-1* was more sensitive than that of the wild-type to elevated Ca^2+^ (25 mM). The visually most striking difference was found under Mn^2+^ deficiency (0.05 µM). Under this condition, the size of the *bicat3-1* shoot was much smaller than that of the wild-type, and leaves of the mutant were curled (Supplemental Figure S9A). Despite this decrease in shoot size, shoot dry mass of *bicat3-1* was comparable to that of the Mn^2+^-deficient wild-type (Supplemental Figure S9B).

**Figure 3:**
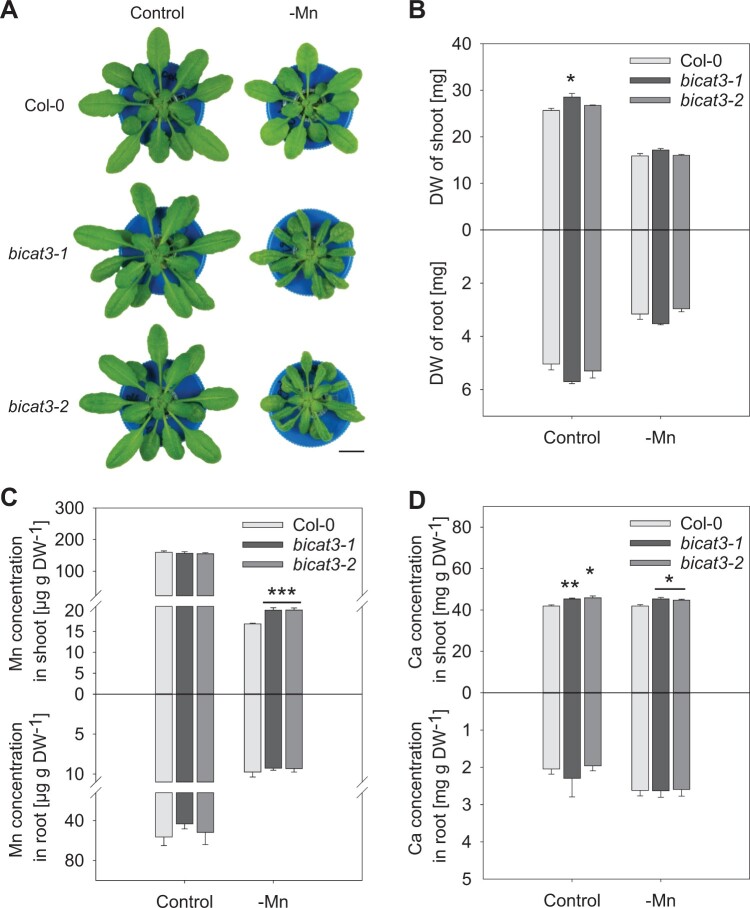
Phenotypes of Col-0 wild-type, *bicat3-1*, and *bicat3-2* under Mn^2+^ deficiency conditions. A, Growth phenotypes of 5-week-old plants cultivated in hydroponics with 3.5 µM Mn^2+^ (control) or 0.05 µM Mn^2+^ (-Mn). Images were digitally extracted for comparison. Scale bar represents 1 cm and applies to all images. B, Shoot and root DW of plants grown as in (A). C and D, Mn^2+^ and Ca^2+^ concentrations in shoots and roots of plants grown as in (A). Data indicate means + se of five biological replicates. Data were analyzed by two-tailed Student’s *t* test to identify significant differences between wild-type and mutant (^*^P < 0.05; ^**^P < 0.01; ^***^P < 0.001). The experiment was repeated twice with similar results.

To determine the role of BICAT3 in Mn^2+^ and Ca^2+^ homeostasis, we measured their concentrations in *bicat3-1* and wild-type. Under adequate supply, *bicat3-1* did not differ in Mn^2+^ concentration compared to the wild-type, but had a higher Ca^2+^ concentration in shoots (Supplemental Figure S9, C and D). However, under Mn^2+^ deficiency and Ca^2+^ toxicity, the Mn^2+^ concentration in *bicat3* shoots was elevated in comparison to the wild-type, while *bicat3-1* shoots accumulated less Mn^2+^ than the wild-type under Mn^2+^ toxicity (Supplemental Figure S9C). Altogether, BICAT3 plays a central role in the plant’s resilience to Mn^2+^ deficiency and contributes to Ca^2+^ and Mn^2+^ homeostasis in Arabidopsis. The *bicat3-2* mutant showed the same Mn^2+^ deficiency-hypersensitive growth phenotype and comparable aberrances in Mn^2+^ and Ca^2+^ concentrations in shoots and roots as *bicat3-1* (Figure 3). Mg^2+^ concentrations were also slightly but significantly lower in Mn^2+^-deficient mutant shoots, whereas Fe^3+^ and Zn^2+^ concentrations were unchanged (Supplemental Figure S10). Interestingly, relative water content was decreased in the mutants, particularly under Mn^2+^ deficiency (Supplemental Figure S11).

### *bicat3* mutants accumulate more manganese in chloroplasts and have improved photosynthesis under Mn^2+^ deficiency

**Figure 4:**
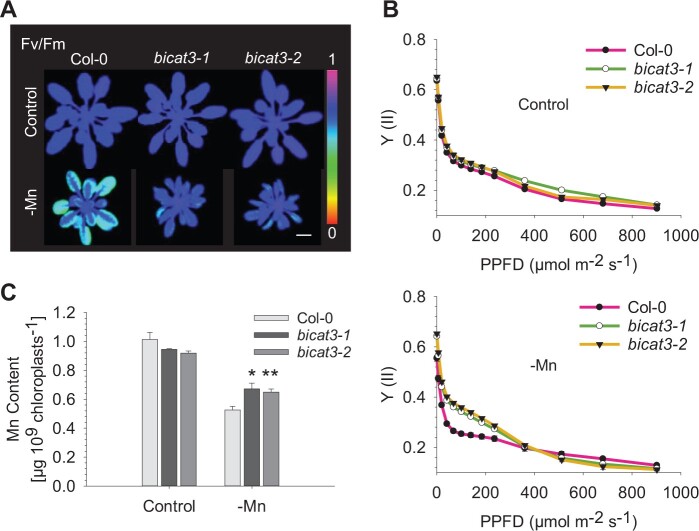
Absence of BICAT3 causes improved photosynthesis and higher Mn^2+^ contents of chloroplasts compared to the wild-type under Mn^2+^ deficiency conditions. A, *Fv/Fm* false color images of 5-week-old Col-0, *bicat3-1*, and *bicat3-2* grown in hydroponics with 3.5-µM Mn^2+^ (control) or 0.05-µM Mn (-Mn). Images were digitally extracted for comparison. Scale bar represents 1 cm and applies to all images. B, Efficiency of PS II [Y (II)] of plants grown as in A. Data indicate means ± se of five biological replicates. C, Mn^2+^ content of chloroplasts isolated from Col-0, *bicat3-1*, and *bicat3-2* plants grown as in (A). Data indicate means + se of four replicates. Data in (B) and (C) were analyzed by two-tailed Student’s *t* test to identify significant differences between wild-type and mutant (^*^P < 0.05; ^**^P < 0.01). The experiment was repeated twice with similar results.

Since a decrease in photosynthetic activity is the most prominent consequence of Mn^2+^ deficiency (Alejandro et al., 2020), we analyzed the chlorophyll fluorescence of the nutrient-starved plants. Intriguingly, despite the retarded growth of *bicat3* mutants (Figure 3), both lines showed a substantially higher maximum quantum yield of PSII (*Fv/Fm*) and effective quantum yield of PSII [Y (II)] (Figure 4, A and B). Whole-plant analysis had shown that *bicat3* mutants accumulate more Mn^2+^ in shoot compared to the wild-type under Mn^2+^ deficiency (Figure 3C;Supplemental Figure S9C). Since we hypothesized that photosynthetic improvement was caused by an enhanced supply of PSII with Mn^2+^, we determined the ion's concentration in isolated chloroplasts by inductively coupled plasma mass spectrometry (ICP–MS). Interestingly, *bicat3-1* and *bicat3-2* chloroplasts contained a higher amount of Mn^2+^ than the wild-type under Mn^2+^ deficiency (Figure 4C), demonstrating that BICAT3 determines cellular Mn^2+^ distribution, including Mn^2+^ supply to chloroplasts, which may subsequently have an effect on photosynthetic activity.

To analyze the dependency of growth defect and improved photosynthetic activity on Mn^2+^ supply level and its reversibility, plants were grown under mildly and severely Mn^2+^-deficient conditions. Both conditions led to an improved *Fv/Fm* in *bicat3-1* compared to the wild-type (Supplemental Figure S12B). This difference increased with increasing degree of Mn^2+^ deficiency, and it could be reversed by a resupply with Mn^2+^ for 1 week. Pronounced differences in Y(II) between *bicat3-1* and wild-type were only obtained under severe Mn^2+^ deficiency, and were also reversible by Mn^2+^ resupply (Supplemental Figure S12C). In contrast to photosynthetic parameters, the abnormal leaf morphology of *bicat3-1* under severe Mn^2+^ deficiency did not change after Mn^2+^ resupply (Supplemental Figure S12A).

To determine if the prominent shoot phenotypes of *bicat3* mutants are controlled systemically by the roots or locally in the shoot, reciprocal grafting of *bicat3-1* and wild-type seedlings was performed. Plants consisting of *bicat3-1* shoot and wild-type root (*b*C) showed identical shoot phenotypes to *bicat3-1* self-grafted plants (*bb*) (Supplemental Figure S13). On the other hand, plants consisting of wild-type shoot and *bicat3-1* root (C*b*) showed a shoot phenotype similar to wild-type self-grafted plants (CC). Regardless of the amount of Mn^2+^ supplied, grafted plants comprising *bicat3-1* roots (C*b* and *bb*) accumulated higher shoot Ca^2+^ concentrations. Taken together, BICAT3 contributes differentially to Ca^2+^ and Mn^2+^ utilization in roots and shoots. Higher Ca^2+^ concentrations in the *bicat3-1* shoots are caused by the absence of BICAT3 in roots, while improved photosynthetic activities, higher shoot Mn^2+^ concentrations, and the curly-leaf phenotype of *bicat3-1* under Mn^2+^ deficiency are due to its function in the shoot.

### *BICAT3* is essential for cell expansion and matrix polysaccharide biosynthesis under low Mn^2+^ availability

One of the most notable changes in the morphology of the *bicat3* mutants is the curly-leaf symptom under Mn^2+^ deficiency (Figure 3A;Supplemental Figure S9A). Cross-sections of *bicat3-1* leaf blades grown under Mn^2+^-deficient conditions showed an extremely compacted mesophyll with diminished intercellular space (Supplemental Figure S14B), accompanied by smaller palisade parenchyma, spongy parenchyma, and lower epidermis cells compared to the wild-type (Supplemental Figure S14, C and D). In addition, sections of spongy parenchyma cells were more circular in the mutant (Supplemental Figure S14E). The more compact tissue likely explains the observed decreased relative water content (Supplemental Figure S11).

Regulation of plant cell size and shape is a highly complex process, encompassing cell growth and cell division. Complex polysaccharides and, to a lesser extent, structural proteins form walls that confine the expansion of plant cells. The Golgi apparatus is a critical site for the posttranslational modification of proteins and matrix polysaccharide biosynthesis. Since Mn^2+^ is an indispensable co-factor for many glycosylation reactions, we analyzed the cell wall composition of *bicat3* mutants, which showed striking differences in growth compared to the wild-type under Mn^2+^ deficiency.

**Figure 5:**
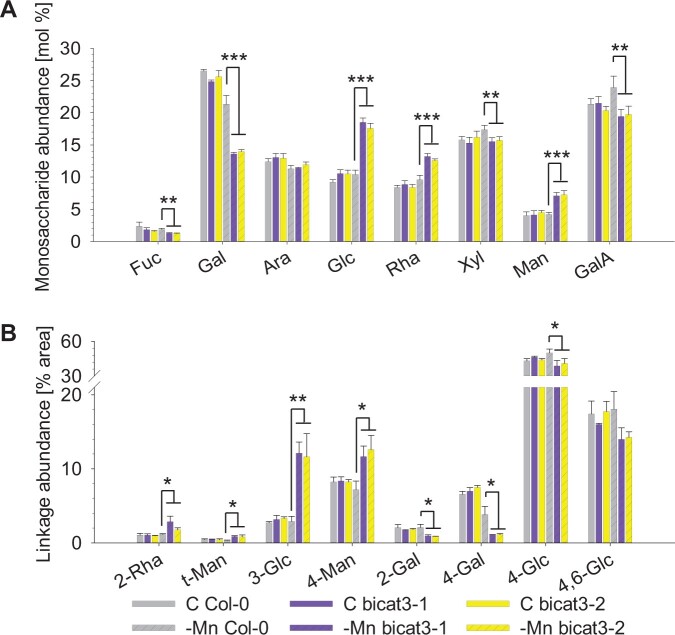
Cell wall matrix sugar components are altered in *bicat3* shoots compared to the wild-type under Mn^2+^ deficiency. A, Monosaccharide composition of shoot cell wall AIR. Col-0 and *bicat3* plants were cultured for 5 weeks in hydroponics with 3.5-µM Mn^2+^ (C) or 0.05 µM Mn (-Mn). B, Glycosidic linkages of Col-0 and *bicat3* shoot cell wall AIR. Values represent molar percentage of total carbohydrates detected. Data in (A) and (B) indicate means + sd of four and three independent biological replicates, respectively. The complete data set can be found in Supplemental Table S1. Data were analyzed by two-tailed Student’s *t* test to identify significant differences between wild-type and mutants (^*^P < 0.05; ^**^P < 0.01; ^***^P < 0.001).

Under control conditions, *bicat3* and wild-type shoot cell walls had similar monosaccharide and glycosidic linkage abundances (Figure 5). However, under Mn^2+^ deficiency, numerous alterations were observed: fucose (Fuc), galactose (Gal), xylose (Xyl), and galacturonic acid (GalA) levels significantly decreased in both *bicat3* mutants as compared to the Mn^2+^-deficient wild-type (Figure 5A). Conversely, the abundance of glucose (Glc), rhamnose (Rha), and mannose (Man) in shoot cell walls of Mn^2+^-deficient *bicat3* was significantly higher compared to those of wild-type plants. Glycosidic linkages, characteristic of different polysaccharides, were massively altered in Mn^2+^-deficient *bicat3* (Figure 5B;Supplemental Table S1), with significant increases of 2-Rha, t-Man, 3-Glc, and 4-Man linkages, accompanied by reductions in 2-Gal, 4-Gal, and 4-Glc. The highly elevated abundance of 3-Glc indicated an accumulation of callose in *bicat3* shoots under Mn^2+^ deficiency, which was confirmed by aniline blue and immunogold staining of callose (Supplemental Figure S15). Callose accumulation is often triggered by an accumulation of reactive oxygen species. 3,3'-diaminobenzidine (DAB) staining indeed showed an accumulation of H_2_O_2_ under Mn^2+^ deficiency in *bicat3-1*, but not in the wild-type (Supplemental Figure S16).

In contrast to the shoots, the monosaccharide and linkage composition of *bicat3* roots was relatively similar to the wild-type (Supplemental Figure S17), indicating organ-specific differences in Mn^2+^ handling and requirements that have yet to be explored.

### Mutation of *BICAT3* hampers seed set and pollen tube growth

**Figure 6:**
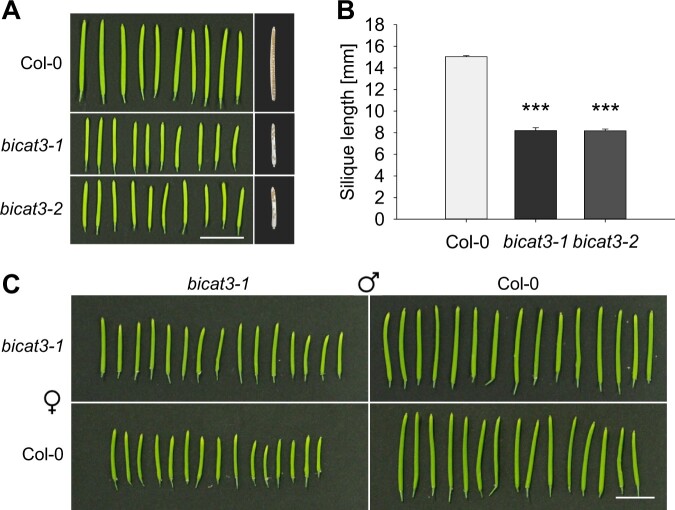
*bicat3* mutants show male gametophyte defects that lead to shorter siliques compared to the wild-type. A, Siliques of Col-0, *bicat3-1*, and *bicat3-2*. Scale bar represents 1 cm and applies to all images. B, Silique length of Col-0, *bicat3-1*, and *bicat3-2.* Data indicate means + se of 30 siliques. Data were analyzed by two-tailed Student’s *t* test to identify significant differences between wild-type and mutant (^***^P < 0.001). C, Reciprocal crossing of Col-0 and *bicat3-1.* Scale bar represents 1 cm and applies to all images. The experiment was repeated twice with similar results.

Besides the vegetative growth aberrance under Mn^2+^ limitation, the *bicat3* lines displayed a severe generative growth defect: both mutants produced short siliques with an incomplete seed set (Figure 6, A and B; Supplemental Figure S18). Additionally, *bicat3* mutant seeds were significantly larger, heavier, and germinated faster than wild-type seeds (Supplemental Figure S19), which is the likely cause of the faster seedling development of the mutants (Supplemental Figure S6–S8). To assess the biological basis of the short-silique phenomenon, reciprocal crosses between *bicat3-1* and wild-type were performed. Siliques obtained from crosses of *bicat3-1* pollen with maternal *bicat3-1* or wild-type showed a reduced length compared to those from the wild-type self-cross, whereas the siliques from *bicat3-1* flowers fertilized with wild-type pollen were comparable to those from wild-type self-cross (Figure 6C). This result indicates a male gametophyte defect to be responsible for the short-silique phenotype.

**Figure 7:**
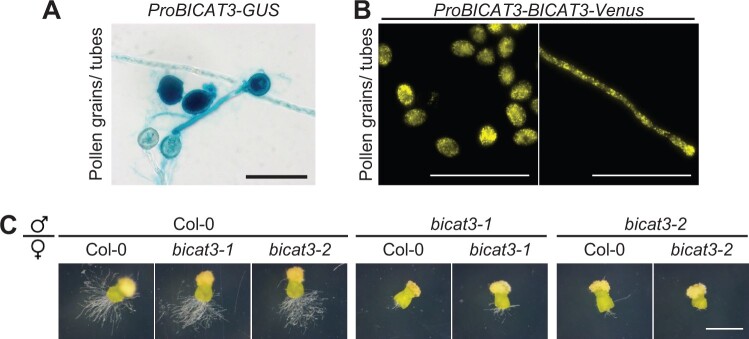
The absence of *BICAT3* hampers pollen tube growth. A, Activity of *BICAT3* promoter in pollen grains and pollen tubes as detected by GUS staining of *ProBICAT3-GUS* line. Scale bar represents 50 µm. B, Activity of *BICAT3* promoter in pollen grains and pollen tubes of *ProBICAT3-BICAT3-Venus* line. Scale bars represent 100 µm. C, *Semi* in vivo pollen tube growth assay of Col-0, *bicat3-1*, and *bicat3-2* germinated on Col-0, *bicat3-1*, or *bicat3-2* pistils. Pictures were taken 12 h after pollination. Images taken from Supplemental Figure S21. Scale bar represents 1 mm and applies to all images.

This observation provoked the question whether *BICAT3* is active in pollen. The expression of *BICAT3* in pollen grains and tubes was confirmed in both *ProBICAT3-GUS* and *ProBICAT3-BICAT3-Venus* reporter lines (Figure 7, A and B), suggesting a direct role of BICAT3 in pollen. Aniline blue staining of pollen tubes grown for 24 h in vivo showed that only a minor proportion of *bicat3-1* pollen tubes grew normally and reached the basal end of the transmitting tract (Supplemental Figure S20). To substantiate the defect in mutant pollen tube elongation, pistils were pollinated and the top part of pollinated pistils, including stigma and style, excised and subsequently cultured for 12 h (Figure 7; Supplemental Figure S21). Under those *semi* in vivo conditions, only few *bicat3-1* and *bicat3-2* pollen tubes grew out of the excised wild-type and mutant pistils after they were cultured on medium for 12 h (Figure 7C). Contrastingly, numerous wild-type pollen tubes penetrated the stigma and style of wild-type and mutant pistils and continued to grow under the same conditions. The abnormal growth of pollen tubes can be complemented by the expression of *BICAT3* driven by its native promoter (Supplemental Figure S21). Together, these results indicated that BICAT3 is critical for pollen tube elongation and consequently seed production.

### *bicat3* pollen tubes accumulate less low methyl-esterified homogalacturonan compared to wild-type pollen tubes

Cell wall polysaccharides such as pectin are crucial for pollen tube extensibility and growth (Hepler et al., 2013; Mollet et al., 2013; Dehors et al., 2019), suggesting that *bicat3* pollen tubes may have altered cell walls. In addition to the retarded pollen tube growth of *bicat3* mutants observed in vivo and in *semi* in vivo growth assays (Figure 7C;Supplemental Figure S20), *bicat3* pollen tubes showed unusual swelling and branching in vitro (Supplemental Figure S22). Ruthenium red primarily stained pectin at the tip of wild-type and complementation line pollen tubes, which was reduced in both *bicat3* mutants and accompanied by elevated staining at the branch points.

**Figure 8:**
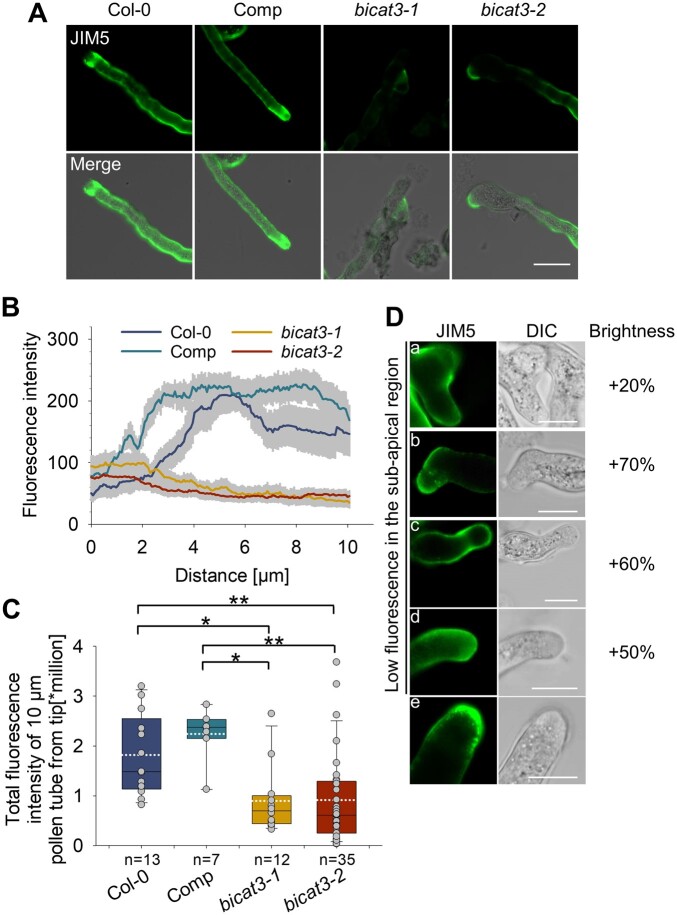
*bicat3* mutant pollen tubes contain less partially methylesterified HG with more variable deposition compared to the wild-type. A, Immunofluorescence labeling of in vitro-grown pollen tubes with JIM5 monoclonal antibody. Images were taken with identical settings. Scale bar represents 20 µm and applied to all images. B, *bicat3* mutant pollen tubes contain a similar amount of JIM5-labeled partially methylesterified HG as the wild-type at the tip, but less in sub-apical regions. Fluorescence intensity of pollen tubes (10 µm from tip) of Col-0, *bicat3-1* complementation line, and *bicat3* mutants. Data indicate means ± se of at least six biological replicates. C, Fluorescence intensity of JIM5-labeled pollen tube apex (0–10 µm from tip) of Col-0, *bicat3* complementation line, and *bicat3* mutants. The box plot shows the range of the values (upper and lower bar), median (black line), means (dotted white line), and the lower and upper quartiles. Single data points are indicated by gray circles. Data were analyzed by Kruskal–Wallis one-way analysis of variance on ranks with Dunn’s method to identify significant differences between wild-type and mutants or Comp and mutants (^*^P < 0.05; ^**^P < 0.01). D, Heterogeneity of JIM5 labeling of in vitro-grown *bicat3* pollen tubes. a–d, Pollen tubes show low fluorescence in the sub-apical region and a, higher fluorescence at the base of a branch; b, higher fluorescence at the tip and very low fluorescence at the swollen part; c, lower fluorescence at the swollen shank and higher fluorescence at a constricted section; d, homogenous but low fluorescence at the apical and sub-apical region. e, Exceptional tubes show high fluorescence at the apex. Scale bars represent 10 µm. The experiment was repeated twice with similar results.

Pectin deposition was further analyzed by immunostaining of in vitro-grown pollen tubes with the JIM5 monoclonal antibody (Dardelle et al., 2010), recognizing partially methylesterified homogalacturonan (HG). Wild-type pollen tubes accumulated partially methylesterified HG in their apical region except for the very tip (Figure 8, A and B). Staining of partially methylesterified HG was severely reduced in both *bicat3* mutants. In typical *bicat3* tubes, the low, wild-type like intensity at the apex did not increase, but rather decreased, in the subapical region (Figure 8B), causing an overall lower staining intensity (Figure 8C). However, staining patterns of individual pollen tubes varied considerably. Partially methylesterified HG showed a heterogeneous distribution in swollen and branched *bicat3* pollen tubes with increased accumulation at branch points or tips and a much lower abundance in the sub-apical region, in particular in swollen sections (Figure 8D). However, as indicated by the brightness adjustment, staining intensity in these accumulation hotspots was much weaker than that in areas of high fluorescence in wild-type tubes. In some notable exceptions, higher intensity that extended to the very tip was observed (Figure 8, C and D). The aberrant partially methylesterified HG distribution was abolished by complementation of the *bicat3-1* mutant with the wild-type *BICAT3*, confirming that the absence of BICAT3 was causal for this phenotype (Figure 8, A–C). In summary, the data demonstrate the requirement of BICAT3 for pectin deposition in pollen tubes, whereby the heterogeneity in growth defects is mirrored by a heterogeneity in pectin deposition aberrances.

### The physiological relevance of BICAT3 is distinct from that of ECA3

**Figure 9:**
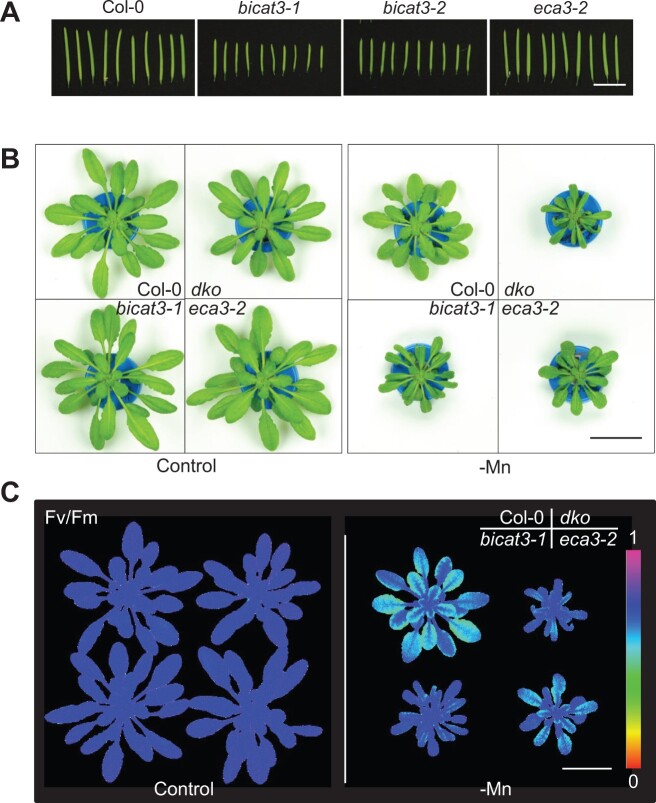
BICAT3 and ECA3 are both involved in Mn^2+^ efficiency, but with distinct roles. A, Siliques of Col-0, *bicat3-1*, *bicat3-2*, and *eca3-2*. Scale bar represents 1 cm and applies to all images. B, Growth phenotypes of 6-week-old Col-0, *bicat3-1*, *eca3-2*, and *dko* (*bicat3-1 eca3-2*) plants in grown in hydroponics with 3.5 µM Mn^2+^ (control) or 0.05 µM (-Mn). Scale bar represents 3 cm and applies to all images. C, *Fv/Fm* images of Col-0, *bicat3-1*, *eca3-2*, and *dko* plants grown under control and Mn^2+^-deficient conditions. Images were digitally extracted for comparison. Scale bar represents 3 cm and applies to all images. The experiments were repeated twice with similar results.

The growth defect of *bicat3* mutants was only apparent at low Mn^2+^ supply, and their male-sterility was incomplete. Both phenotypes are related to alterations in matrix polysaccharide composition of the cell wall, most likely inflicted by malfunction of Mn^2+^-dependent glycosyl transferases in the Golgi. Since Mn^2+^ is essential for these reactions, mechanisms other than BICAT3 have to exist to supply the Golgi with the metal. The CDF-type Mn^2+^ transporter MTP11 has been localized to the Golgi apparatus (Peiter et al., 2007). However, *mtp11* mutants have a phenotype opposite to that of *bicat3* mutants, showing hypertolerance to Mn^2+^ limitation. In contrast, mutants of the Mn^2+^-transporting P-type ATPase ECA3, which has also been localized to the Golgi, are hypersensitive to low Mn^2+^ supply (Mills et al., 2008). We therefore hypothesized that ECA3 may operate alongside BICAT3, and that double mutants would hence show exacerbated phenotypes. However, in contrast to *bicat3* mutants, silique length was not reduced in an *eca3* mutant as compared to the wild-type (Figure 9A). Together with the very low expression of *ECA3* in pollen (Qin et al., 2009), this indicates that this pump does not play a notable role in Mn^2+^ supply of the pollen Golgi.

The situation was different in vegetative tissues. As apparent in Figure 9B, the rosette size of 6-week-old plants grown under Mn^2+^ deficiency was diminished in *eca3-2* as well as in *bicat3-1*, as observed before (Figure 3A), and the double mutant displayed an exacerbation of this phenotype, which was accompanied by a likewise decrease of root and shoot dry weight (DW) (Supplemental Figure S23). The effect of both mutations on photosynthesis was unequal. Whereby the decrease in *Fv/Fm* and Y(II) under Mn^2+^ deficiency was strongly alleviated in *bicat3-1* and the double mutant, this was less pronounced in *eca3-2* (Figure 9B;Supplemental Figure S23, D and E). The previously observed increase in Mn^2+^ concentration in Mn^2+^-deficient *bicat3* was more moderate in *eca3-2* and more pronounced in the double mutant (Supplemental Figure S23B), whereas for Ca^2+^, an additive effect of both mutations was not noticeable (Supplemental Figure S23C). The mutant phenotypes indicate that BICAT3 and ECA3 do not function redundantly, but partly cooperate for efficient plant performance under limited Mn^2+^ availability.

## Discussion

Manganese is an indispensable metal for a diversity of processes in plants. One of the essential roles of Mn^2+^ is as a cofactor of enzymes that mediate protein glycosylation and polysaccharide biosynthesis in the Golgi apparatus (He et al., 2021). Despite the crucial role of Mn^2+^ in the plant’s Golgi, little is known about the transport proteins responsible for the Mn^2+^ supply of Golgi functions. In this work, we found that the ubiquitously expressed trans*-*Golgi-localized BICAT3 protein transports Mn^2+^ and Ca^2+^ in a heterologous expression system. In the absence of BICAT3, plants showed numerous aberrances under Mn^2+^ deficiency, such as growth retardation, changes in rosette morphology, and defects in glycan synthesis, albeit a strikingly better maintenance of photosynthetic activity was observed. Besides, *bicat3* mutants have a defective pollen tube growth with aberrant pectin accumulation and, ensuing from this, produce shorter siliques with fewer seeds. Thus, BICAT3 plays a vital role in maintaining Golgi functions for both vegetative and reproductive growth of the plant. The biochemical phenotypes of BICAT3 revealed in this work point to a direct involvement in Mn^2+^ homeostasis, whereby additional roles for Ca^2+^-dependent processes cannot be excluded and may also underlie some of the macroscopic phenotypes.

### BICAT3 determines responses to varying Mn^2+^ and Ca^2+^ supply

Heterologous expression of *BICAT3* in yeast mutants that are sensitive to different metals showed that BICAT3 restored their Ca^2+^ and Mn^2+^ tolerance (Figure 2). *BICAT3*-complemented *pmr1Δgdt1Δ* yeast grew comparably to the *pmr1Δ* single mutant under 600-mM CaCl_2_, indicating the functional conservation of BICAT3 and GDT1. The complementation experiments suggest that BICAT3 is specifically involved in Ca^2+^ and Mn^2+^ transport when heterologously expressed in yeast. Such a poor selectivity between Ca^2+^ and Mn^2+^ is characteristic of transport proteins belonging to numerous families. In plants, these include ER-type Ca^2+^-ATPases (e.g. ECA1 Wu et al., 2002) and ECA3 (Li et al., 2008; Mills et al., 2008), Calcium Exchangers (e.g. CAX2; Shigaki et al., 2003), as well as BICAT proteins, for which transport of Ca^2+^ and Mn^2+^ have been directly demonstrated in BICAT1 and 2 (Schneider et al., 2016; Wang et al., 2016; Frank et al., 2019). The physiological relevance of the dual functionality is largely unresolved, and it is unclear how homeostasis of both elements is regulated independently by nondiscriminating transporters (He et al., 2021).

Both young (2-week-old) and older (5-week-old) plants of *bicat3* loss-of-function mutants showed altered responses to different Ca^2+^ and Mn^2+^ supply levels compared to the wild-type. In conditions of Mn^2+^ deficiency and Ca^2+^ toxicity, *bicat3* mutants performed worse than the wild-type, while they responded superiorly to Mn^2+^ toxicity and Ca^2+^ deficiency (Supplemental Figure S6–S9). The most striking growth difference between *bicat3* mutants and the wild-type was found under Mn^2+^ starvation. Collectively, these results indicate a role of BICAT3 in Mn^2+^ and Ca^2+^ homeostasis of Arabidopsis, in particular during low Mn^2+^ availability. It remains to be clarified if the altered response of *bicat3* mutants to extreme Ca^2+^ supply is due to a Ca^2+^ transport activity of the transporter, or due to the Mn^2+^-versus-Ca^2+^ selectivity of the processes that back up BICAT3. In mammalian cells, Mn^2+^-mediated rescue of glycosylation defects inflicted by deletion of the BICAT3 homolog, TMEM165, is likely dependent on ER-resident SERCA-type ATPases, indicating the ER potentially contributes to the supply of Mn^2+^ to the Golgi (Houdou et al., 2019). In contrast, the Golgi-localized SPCA1 Ca^2+^/Mn^2+^-ATPase did apparently not contribute to the rescue of Mn^2+^-dependent glycosylation in *tmem165* mutants (Houdou et al., 2019). It is unresolved if the normal vegetative growth and glycosylation pattern of *bicat3* mutants under control conditions are due to other Golgi-localized Mn^2+^ transport proteins or a supply via vesicular trafficking from the ER. The comparative analysis of *bicat3*, *eca3*, and *bicat3 eca3* mutants (Figure 9) indicated that the Golgi-localized Mn^2+^ pump ECA3 does most likely not operate alongside BICAT3, but both proteins may function in series, either subcellularly in different cisternae, or in different cell types.

The *bicat3* mutants were more tolerant to Mn^2+^ toxicity in both plate and hydroponic cultures under long-day and short-day conditions, represented by a higher shoot and root biomass than that of the wild-type (Supplemental Figures S8 and S9). Interestingly, *bicat3* shoots accumulated less Mn^2+^ compared to those of the wild-type under elevated Mn^2+^ conditions, which likely explains the better growth of *bicat3* under Mn^2+^ toxicity. In contrast to our results, a previous study reported a *higher* sensitivity of *bicat3* (*pml3*) mutants to Mn^2+^ toxicity compared to the wild-type (Yang et al., 2021). These findings are difficult to reconcile with our findings and might be due to different media (nutrient composition and concentration), growth conditions (light cycle and light intensity), or plant age.

### BICAT3 determines matrix polysaccharide biosynthesis

BICAT3 operates as a Golgi-localized Mn^2+^ transporter, and the biosynthesis of the cell wall matrix polysaccharides, pectin, and hemicellulose, is mediated by Golgi-localized glycosyl transferases, many of which require Mn^2+^ (He et al., 2021). We, therefore, hypothesized that the prominent aberrations in leaf shape, and cell size and morphology of Mn^2+^-deficient *bicat3* mutants are related to an altered cell wall matrix. This notion was strongly supported by a combined analysis of monosaccharide and glycosidic linkage composition, which showed substantial changes in pectin structure (Figure 5). A reduction in the abundance of GalA indicated that the overall pectin content was lower in shoot cell walls of Mn^2+^-deficient *bicat3* mutants as compared to those of the wild-type. Additionally, the abundance of Rha and the 2-Rha linkage was increased, while that of Gal and the 4-Gal linkage was severely decreased. These aberrances indicate that the β-1,4-galactan side chain substitution of RG I was affected in *bicat3* shoot cell walls (He et al., 2021). In Arabidopsis, this addition of galactose from UDP-α-D-Gal to growing β-1,4-galactan chains is catalyzed by highly Mn^2+^-dependent galactan synthases (GALS1 to 3) (Ebert et al., 2018; Laursen et al., 2018), which were probably not sufficiently supplied with their metal cofactor in the *bicat3* mutants.

Galactan side chain substitution of RG I occurs in the trans cisternae of the Golgi (Zhang and Staehelin, 1992), which corresponds to the primary localization of BICAT3, as identified by high-resolution CLSM and immunogold staining (Figure 1). In this respect, a previously claimed preferable localization of BICAT3 (PML3) in the cis-Golgi (Yang et al., 2021), which was concluded from conventional CLSM data, cannot be confirmed.

Along with xyloglucans, galactan domains of RG I interact with cellulose microfibrils, hence maintaining cell wall structure (Zykwinska et al., 2007). A truncation of galactan side chains also interferes with porosity and thus water-binding capability of the plant cell walls (Klaassen and Trindade, 2020). However, a *gals1 gals2 gals3* triple mutant did not display a macroscopic phenotype (Ebert et al., 2018), suggesting that the severe reduction of 4-Gal does not account for the impaired growth of Mn^2+^-deficient *bicat3* mutants.

By association with receptor-like proteins (RLP) or wall-associated kinases (WAK), pectins not only modulate the extensibility, flexibility, and rigidity of the cell wall, but are also involved in feedback regulation to control wall homeostasis during cell expansion (Franck et al., 2018). Thereby WAKs are essential for cell expansion and pathogen resistance (Kohorn and Kohorn, 2012). WAK2 binds to pectin in the cell wall, and *WAK2* antisense plants show small and curly-bent leaf phenotypes with smaller cell size (Wagner and Kohorn, 2001). The curled-leaf phenotype of *bicat3* might thus be related to signaling pathways induced by the defects in pectin biosynthesis under Mn^2+^ deficiency.

As compared to the shoot, aberrances in monosaccharide composition and linkage in the root were much less pronounced (Supplemental Figure S17). This is in disagreement with a previous analysis, which describes a morphological disintegration of the root apex (Yang et al., 2021). This was not observed in any of our experiments, with exemplary images shown in Supplemental Figure S24. Again, the reason for this disagreement is unclear and may lie in the growth conditions.

The abundance of callose was drastically increased in cell walls of *bicat3* shoots (Supplemental Figure S15), which coincided with a higher abundance of Glc and 3-Glc linkage (Figure 5). Callose is not synthesized in the Golgi, but by callose synthases at the plasma membrane. Hence, callose deposition in *bicat3* mutants likely is an indirect response to glycan defects inflicted by the absence of BICAT3. Callose deposits play multiple roles in plants, in particular in the regulation of plasmodesmata and in responses to biotic and abiotic stresses (De Storme and Geelen, 2014; Rissel et al., 2017; Hunter et al., 2019; Wang et al., 2021). Recently, callose synthesis was reported to be induced by Ca^2+^ deficiency, preventing cell death (Shikanai et al., 2020). Due to its function in cross-linking pectins, Ca^2+^ deficiency is believed to induce cell wall damage that is counteracted by callose depositions. In support of this, expression of a cell wall damage-induced transcription factor, OsWRKY42, in Arabidopsis leads to intensified callose accumulation (Pillai et al., 2018). We therefore hypothesize that the abnormal accumulation of callose in *bicat3* mutants is a result of cell wall defect-induced signaling pathways under Mn^2+^ deficiency. This is supported by the increased accumulation of the ROS H_2_O_2_ (Supplemental Figure S16), which is a common component of those pathways.

A previous study also found alterations in shoot cell wall composition of *bicat3* mutants, which, however, did not fully correspond to our observations (Yang et al., 2021). While similar Alcohol-insoluble residue (AIR) preparation methods were used in the two studies, different analytical methods (high-performance anion-exchange chromatography coupled with pulsed amperometric detection [HPAEC-PAD] here and gas chromatography–mass spectrometry [GC–MS] in Yang et al. (2021)) were used for the final quantification. While the GC–MS method only detected neutral sugars after derivatization, HPEAC-PAD allowed us to also quantify the abundance of GalA, the main building block of pectin. Nevertheless, the major changes in the Mn^2+^-deficient mutant shoots differed between the two studies: less Man, Ara, Xyl, and Glc compared to the wild-type in Yang et al. (2021); less Gal, but more Glc and Man relative to the wild-type in this work (Figure 5A). These differences should not be the result of the different analytical techniques used, but instead suggest that the precise cultivation conditions likely have a profound effect on modulating the *bicat3* mutant chemotypes.

Taken together, under limited Mn^2+^ supply, the loss of *BICAT3* causes multiple direct and indirect changes in cell wall polysaccharide biosynthesis. It remains to be determined which of the observed macroscopic phenotypes are a direct result of which of those alterations.

### BICAT3 determines pollen tube growth and seed set

Apart from their drastic vegetative phenotype under Mn^2+^ deficiency, *bicat3* mutants were severely affected in reproduction, producing fewer but larger seeds in shorter siliques (Figure 6; Supplemental Figure S19). As evident from reciprocal crossing, this reduced fertility was caused by a defect in the male gametophyte (Figures 6, C and 7, C). Pollen tubes of *bicat3* mutants showed highly heterogeneous morphologies and were often branched or swollen (Figure 8; Supplemental Figure S22).

We hypothesized that the defective pollen tube growth was caused by a Mn^2+^-dependent function of the Golgi. Elongation of the pollen tube requires the deposition of cell wall material at the tip. The most abundant pectin, HG, is synthesized in the Golgi by HG synthetases of the GAUT family (Atmodjo et al., 2011), which contain a Mn^2+^-binding DXD motif. For the GAUT1:GAUT7 enzyme complex an absolute dependence on Mn^2+^ has been experimentally shown (Amos et al., 2018). Numerous *GAUT* genes are expressed in pollen, most prominently *GAUT13* and *14*. Pollen tubes of the *gaut13 gaut14* mutant are swollen and defective in tube growth (Wang et al., 2013), similar, but more severely, to what we observed in *bicat3* mutants. Also mutants of the functionally redundant *GAUT5*, *6*, and *7*, that are more weakly expressed in pollen, were defective in tube elongation (Lund et al., 2020). In those GAUT mutants, HGs are unconventionally distributed, with weakly esterified HG, detected by the JIM5 antibody, being severely reduced in the *gaut13 gaut14* mutant (Wang et al., 2013). This defect closely resembles our observations on *bicat3* pollen. The abundance of weakly esterified HG in subapical regions was generally severely diminished in *bicat3*, which most likely led to a loss in rigidity and hence in a swelling of the otherwise perfectly cylindrical tube.

The pattern of pectin deposition and the tube morphology showed substantial variability in the *bicat3* mutant, which may be related to varying Mn^2+^ resources in the pollen population, affecting Mn^2+^-dependent processes to different degrees (Figure 8). This was also evident in vivo, where only some pollen tubes extended sufficiently to reach an ovule. The abnormal swelling was always associated with a decreased HG abundance, and abnormal branching was accompanied by a localized HG deposition. In agreement with previous analyses (Chebli et al., 2012; Zhang et al., 2021), the deposition of weakly esterified HG was weaker at the tip of the wild-type tube, and this tip-low pattern was not apparent in the *bicat3* mutants (Figure 8B). However, the distribution of weakly esterified HG in *bicat3* differed somewhat to that found in a previous report, which described a consistently increased deposition at the tip of *bicat3*, with no absolute difference to the wild-type in subapical regions (Zhang et al., 2021). Those different patterns may have been caused by different intensities of Mn^2+^ limitation.

Despite the close match of aberrant HG deposition and growth defects, a contribution of other Mn^2+^-dependent processes to the phenotype cannot be excluded. Naturally, Golgi-localized enzymes involved in the synthesis of further cell wall polyssacharides and glycoproteins may also be affected. For example, MGP4 involved in RG-II biosynthesis (Liu et al., 2011), GALT2-6 and HPGT1-3 involved in AGP biosynthesis (Ogawa-Ohnishi and Matsubayashi, 2015; Basu et al., 2015a, 2015b), and HPAT1-3 involved in extensin biosynthesis (Ogawa-Ohnishi et al., 2013), all show pollen tube growth defects, and any contribution of these proteins to the *bicat3* phenotypes remains to be demonstrated.

### *BICAT3* restricts Mn^2+^ bioavailability for chloroplasts

Intriguingly, although growth of *bicat3* mutants suffered substantially under Mn^2+^ deficiency, photochemical efficiency of the mutants was superior to that of the wild-type (Figure 4). This coincided with moderately higher Mn^2+^ concentrations in shoots of the mutants (Figure 3). Grafting experiments revealed that improved photosynthesis and the morphological phenotype of *bicat3-1* were both caused by the loss of BICAT3 in the shoot (Supplemental Figure S13). Since the photosynthetic light reaction is highly sensitive to Mn^2+^ deficiency (Alejandro et al., 2020), we assumed that the intracellular allocation of Mn^2+^ to chloroplasts may be altered in *bicat3* mutants. Indeed, under Mn^2+^ deficiency *bicat3-1* and *bicat3-2* chloroplasts accumulated more Mn^2+^ than those of the wild-type, rendering the Mn^2+^ limitation less severe (Figure 4). This indicates that BICAT3 is able to govern the competition of different cellular compartments for Mn^2+^. The effect of the *bicat3* mutations on photosynthesis was more pronounced than that on chloroplast Mn^2+^ content. However, we do not expect both parameters to be linearly associated but rather in a threshold-like manner. Below a certain concentration of free Mn^2+^ in the chloroplast, synthesis, and stability of the water-splitting complex will be severely affected. A small increase in chloroplast Mn^2+^ concentration may therefore cause a marked improvement of photosynthesis at low Mn^2+^ supply. It currently cannot be discerned whether Mn^2+^ supply of other organelles, such as mitochondria, is also improved in the absence of BICAT3.

In this respect, the TGN-localized transporter NRAMP2, in conjunction with the vacuolar transporters NRAMP3 and 4, has previously been proposed a role in inter-organellar Mn^2+^ distribution toward chloroplasts under Mn^2+^-limiting conditions. This appears to be supported by decreased or increased chloroplast Mn^2+^ concentrations in *NRAMP2* knockdown or overexpression plants, respectively (Alejandro et al., 2017). However, since expression of *NRAMP2*, *3*, and *4* in leaves is strictly confined to the vascular system (Thomine et al., 2003; Alejandro et al., 2017), the operation of an NRAMP2-driven Mn^2+^ supply chain of chloroplasts and a possible interference of BICAT3 with this process demands further scrutiny.

Taken together, BICAT3 occupies a central position in Mn^2+^ use efficiency of Arabidopsis, both directly by supplying Golgi cisternae with Mn^2+^, and indirectly by determining intracellular Mn^2+^ distribution. Aberrant Mn^2+^ homeostasis in the Golgi apparatus causes specific defects in glycosyl transfer reactions, affecting cell size, leaf shape, and the tip growth of pollen tubes. The fact that this Mn^2+^ transporter and Mn^2+^-dependent processes depending on it are located in the same cisternae supports the model of a subcompartmental arrangement of the polysacharide assembly line. Numerous Mn^2+^ transporters have by now been identified in the plant’s secretory pathway. These do not function merely redundantly, but have distinct and sometimes even opposite physiological functions. The differential relevance of BICAT3 and ECA3 is exemplary for this not yet fully understood division of labor. Elucidation of the functional interaction of those Mn^2+^ transporters on subcompartmental, subcellular, and tissue level is a consequential next step to be pursued.

## Materials and methods

### Plant materials and growth conditions

Arabidopsis (*A. thaliana*) T-DNA insertion mutant lines were supplied by the Nottingham Arabidopsis Stock Centre. *bicat3*-*1* (GABI_027F07) and *bicat3-2* (SALK_97998) mutants with a Columbia-0 (Col-0) background were obtained from the GABI collection and the SALK collection, respectively (Ülker et al., 2008). Homozygous plants were identified by using gene-specific primers as listed in Supplemental Table S2. The insertions were identified with GABI_LB and SALK_LB primers for the lines of the GABI collection and the SALK collection, respectively. For identification of gene knockout, gene-specific primers were used in reverse transcription–PCR (RT–PCR). Amplification of *Beta-6 Tubulin* (*TUB6*; At5g12250) was used as an internal control. To quantify the expression of *BICAT3* in the knock-down mutant, RT–qPCR was performed. *Actin2* (*ACT2*; At3g18780) was used as housekeeping gene.

To generate a *bicat3-1* complementation line, a genomic DNA fragment containing the 5′-UTR and promoter region (2,017-bp upstream of the ATG), the coding sequence, and the 3′-UTR (493-bp downstream of the TAG) of *BICAT3* was cloned into pGreenII, yielding pGreenII-gBICAT3. *bicat3-1* plants were stably transformed with the pGreenII-gBICAT3 construct by floral dip (Clough and Bent, 1998). Seedlings were selected by spraying with Basta. This line was used in the *semi* in vivo pollen assay and in immunostaining of pollen tubes.

To assemble the pCAMBIA2300u-PrBICAT3-BICAT3-Venus-tNos vector, the region 1,811-bp upstream of the *BICAT3* start codon, *BICAT3-Venus*, and a *Nos* terminator were amplified from Col-0 genomic DNA, pART7-BICAT3-Venus, and pILTAB381, respectively, using USER cloning-compatible primers (Nour-Eldin et al., 2006). The pART7-BICAT3-Venus vector was generated by inserting the BICAT3 CDS into pART7-Venus using XmaI-containing primers. *bicat3-1* plants were stably transformed with the pCAMBIA2300u-PrBICAT3-BICAT3-Venus-tNos construct by floral dip. Seedlings were selected on half-strength Murashige and Skoog (1/2 MS) plates containing kanamycin (50 μg mL^−1^). This line was used in pollen tube and immunogold localization experiments.

To cultivate plants on 1/2 MS agar plates, seeds of wild-type and transgenic Arabidopsis were sterilized in 70% (v/v) ethanol for 1 min, 30% (v/v) bleach for 5 min and rinsed 5 times with sterile water. Seeds were stratified for 3 days at 4°C in darkness.

For testing the growth of plants under Mn^2+^ deficiency on plates, stratified seeds were sown on self-made 1/2 MS agar with or without Mn^2+^ (Supplemental Table S3), containing 8 g L^−1^ agar (No. 2,266.1, Carl Roth, Karlsruhe, Germany) and 2.5 g L^−1^ sucrose. Plants were cultured for 2 weeks in a growth cabinet (AR75, Percival Scientific, Perry, IA, USA) set to 21°C day and 19°C night temperature and 16-h light period with a light intensity of 150-µmol m^−2^ s^−1^ photons. For root length measurements, plates were scanned every other day from the 4th until the 14th day. Primary root length of seedlings was measured by the NeuronJ plugin of Fiji.

For testing the growth of plants under Ca^2+^ and Mn^2+^ toxicity on plates, stratified seeds were precultured for 7 days on 1/2 MS agar including B5 vitamins (M0231, Duchefa Biochemie, Haarlem, the Netherlands), containing 8-g L^−1^ agar (Phyto Agar P1003, Duchefa Biochemie) and 2.5-g L^−1^ sucrose, adjusted to pH 5.8 with KOH. Plants were cultured in a growth cabinet as above. Seven-day-old seedlings were transferred to new 1/2 MS media with or without additional 50-mM CaCl_2_ or 1-mM MnSO_4_, and cultured for another 8 days. Plates were scanned every other day after transferring the seedlings for root length measurement. Primary root length of seedlings was measured as above.

For cultivation of Arabidopsis in hydroponics, stratified seeds were sown on 1/2 MS plates as above. After 12 days of growth, seedlings were transferred to seedling holders created by removing the tip of a 1.5-mL centrifuge tube and a cylindrical polyethylene foam piece (BKF A. Fleuren, Friesoythe, Germany). A nutrient solution based on Peiter et al. (2007) was used as hydroponics basal medium (Supplemental Table S3). After growing for 10 days in 1-L containers with basal medium, roots and seedling holders were washed 3 times with ddH_2_O. Seedlings were transferred to 4-L containers containing control or treatment media, and cultivated further for 2 weeks. In the case of recovery experiments, plants were cultivated in basal media for one more week after 2 weeks of Mn^2+^ deficiency treatment. Plants on plates and in hydroponics were cultured in a growth cabinet (ATC26, Conviron, Winnipeg, Canada) set to 21°C day and 19°C night temperatures and 10-h light period with a light intensity of 150-µmol m^−2^ s^−1^ photons. The nutrient solution was changed twice a week.

### Subcellular localization

The full-length *BICAT3* coding sequence (CDS) was used to construct pART7-BICAT3-Venus and pART7-BICAT3-mCherry. Organellar markers for co-localization experiments were obtained from Nelson et al. (2007). Protoplasts of the Arabidopsis Col-0 wild-type and an ST-GFP *trans*-Golgi marker line (Hawes and Satiat-Jeunemaitre, 2005) were prepared and transiently transformed with the constructs as previously described (Peiter et al., 2007). Twenty four hours after transformation, fluorescence was observed by confocal laser microscopy using a LSM 880META containing a PMT detector and with a planapochromatic lens (63×/1.4 Oil) (Carl Zeiss, Jena, Germany). Images were acquired in lambda mode (Figure 1) or channel mode (Supplemental Figure S2) with a resolution of 1,024 × 1,024 pixels and 512 × 512 pixels, respectively. Excitation light source was a 488-nm multiline ion laser and a 561-nm argon laser. Images were acquired by using the Zeiss ZEN blue software; the display range was optimized by applying the Best Fit function in the software. In channel mode, pictures were acquired by using the multitrack function. Venus and mCherry were detected by using a 446–553 and 588–651-nm band pass filter, respectively. Images in lambda mode were acquired at a collection bandwidth of 410–651 nm in plane scan mode for single lines with four repetitions. GFP and mCherry signals were separated by linear unmixing using previously generated spectra obtained from protoplasts transiently transformed with a pART7 vector containing the respective fluorophore.

For localization of fusion proteins in Golgi cisternae, *Agrobacterium tumefaciens* GV3101 containing the desired constructs were grown to an optical density (600 nm) of 0.6, harvested, and resupended in Infiltration Buffer (10-mM MgCl_2_, 5-mM MES [KOH pH 5.3], 150-µM acetosyringone). The desired combinations were mixed, and leaves of 5-week-old *Nicotiana benthamiana* plants were infiltrated with a 1-mL syringe. After 48 h, infiltrated leaf pieces were cut out and water-soaked using a syringe. The leaf disc was placed on a high-precision coverslip (170 ± 5 µm, Marienfeld, Lauda-Königshofen, Germany) with water as mounting medium and covered with a second coverslip. Images were taken with a STELLARIS 8 microscope (Leica, Wetzlar, Germany) equipped with a HC PL APO CS2 63×/1.30 glycerol objective. A White Light Laser was used as excitation light source, and GFP and mCherry were excited at 489 and 587 nm, respectively. Emitted light was detected with the Hybrid-Detector (HyD) with the respective ranges of 494–572 and 592–649 nm. Images were edited using the LAS X software (Leica).

For ultrastructural localization of BICAT3-Venus, leaf segments were cryo-fixed and freeze-substituted as described (Tabassum et al., 2020). For immunolabeling of ultrathin sections, we used a polyclonal anti-Venus antibody (St John’s Laboratory, London, UK; diluted 1:300) detected by a rabbit anti-goat secondary antibody conjugated with 10-nm gold (G5527, Sigma; diluted 1:100). Sections were poststained with uranyl acetate and lead citrate using an EMSTAIN instrument (Leica, Wetzlar, Germany) and observed with a Libra 120 transmission electron microscope (Carl Zeiss) operating at 120 kV. Images were taken by using a dual-speed on-axis SSCCD camera (BM-2k-120; TRS, Moorenweis, Germany).

For observation of BICAT3-Venus in pollen and pollen tubes, pollen of *bicat3-1* stably expressing *BICAT3-Venus* was harvested and cultured in vitro for 3 h as described below and subsequently observed by fluorescence microscopy using a AxioCam MRm Rev. 3 camera mounted on an Axio Observer.Z1 (Carl Zeiss) microscope equipped with filter set FS46HE (excitation 488–512 nm, emission 520–550 nm) and a Plan Apochromatic lens (40×/1.3 oil). Excitation light source was a HXP-120 lamp. Images were acquired by using the Zeiss ZEN blue software; the display range was optimized by applying the Best Fit function of the software.

### Histochemical GUS staining

For promoter-GUS studies, the *BICAT3* promoter region 1,811-bp upstream of the start codon was amplified from Col-0 genomic DNA and cloned into pBl101 using Xma I, upstream of the *uidA* gene (Peiter et al., 2007). Arabidopsis Col-0 plants were stably transformed with this construct by the floral-dip method (Clough and Bent, 1998). Transformants were selected using kanamycin. Plants were cultivated either on sterile 1/2 MS agar plates or in soil. Plant materials were transferred to GUS staining solution containing 100-mM sodium phosphate buffer (pH 7.0), 10-mM EDTA, 3-mM K_4_[Fe(CN)_6_], 0.5-mM K_3_[Fe(CN)_6_], 0.1% (v/v) Triton X-100, and 2-mM 5-Bromo-4-chloro-3-indolyl-beta-d-glucuronic acid (X-Gluc, X-Gluc Direct, Malaga, Spain) in DMSO. Samples were vacuum-infiltrated with staining solution 2 times for 5 min and subsequently stained for 3 h at 37°C. Chlorophyll was removed by 80% (v/v) ethanol. Of 15 independent promoter-GUS lines, 13 showed the same staining pattern. Two homozygous single-insertion lines were chosen for in-depth analysis. The stained tissues were documented with an AxioCam HRc digital camera (Carl Zeiss) mounted on a SteREO Discovery V.20 stereomicroscope (Carl Zeiss).

For GUS staining of pollen and pollen tubes, pollen was harvested and cultured in vitro for 3 h as described below and subsequently stained with GUS staining solution for 3 h at 37°C.

### Yeast complementation

The pRS416-BICAT3 plasmid was prepared by inserting the *BICAT3* full-length sequence in pRS416 using BamHI and SalI. Yeast (*S. cerevisiae*) wild-type (BY4741, Y00000, Euroscarf, Oberursel, Germany) and mutants BY4741-*pmr1*Δ and BY4741-*gdt1*Δ*pmr1*Δ were transformed with pRS416 or pRS416-BICAT3 as described (Frank et al., 2019). Transformants were selected on synthetic complete (SC) media lacking uracil. Yeast drop assays were performed on SC-Ura (pH 5.5) and AP-Ura (pH 5.5) media to assess Mn^2+^ toxicity and Ca^2+^ toxicity, respectively, as described previously (Peiter et al., 2007; Frank et al., 2019).

### Metal concentration measurements

For determination of metal concentrations, shoots were excised and roots were sequentially washed in ice-cold washing buffer I (1-mM MES-KOH, pH 5.8) for 10 min, in washing buffer II (10-mM EDTA and 1-mM MES-KOH, pH 5.8) for 10 min, and in MilliQ-H_2_O for several times. Shoots and roots were dried for 3 days at 65°C in a drying cabinet (Linn High Therm, Eschenfelden, Germany). Dried shoots and roots were weighed into PFA vessels (CEM, Matthews, NC, USA) and digested in 65% (v/v) HNO_3_ for 20 min at 190°C in a MARS 5 Xpress (CEM) microwave oven with the temperature ramped to 190°C in 15 min. Elements were analyzed by microwave plasma—atomic emission spectrometry (MP-AES; 4210, Agilent Technologies, Palo Alto, CA, USA).

For the determination of metal concentrations in chloroplasts, plants were cultivated and treated in a hydroponic system as described above. After removing midrib veins, leaf samples (around 2-g fresh weight) were homogenized in 45-mL homogenization buffer (0.4-M sorbitol, 20-mM Tricine-NaOH [pH 8.4], 10-mM EDTA, 1-g L^−1^ BSA, 5-mM NaHCO_3_, and 1 -mM MgCl_2_) by using a blender (Waring, Stamford, CT, USA) at low speed for 5 s. The mixture was filtered through 75-µm mesh (prewetted with homogenization buffer) into 50-mL tubes. Subsequently, the extracts were squeezed through two layers of prewetted 10-µm nylon mesh and concentrated at 1,500 *g* for 5 min. The pellet was resuspended in at least 2-mL washing buffer (80-mM sorbitol, 4-mM Tricine-NaOH [pH 8.4], 0.5-mM EDTA, and 1-mM MgCl_2_) and then loaded gently onto a discontinuous 40/85% (w/v) Percoll gradient in washing buffer followed by centrifugation (4,000 *g*, 15 min, 4°C) in a swinging bucket rotor. Around 1-mL intact chloroplasts were recovered from the 40/85% (w/v) Percoll interphase. Three volumes of washing buffer (around 3 mL) were added, gently mixed, and centrifuged (10 min at 200 *g* followed by 1 min at 1,700 *g*; at 4°C). Chloroplasts were washed 3 times with 2-mL cold washing buffer. The integrity of the chloroplasts was verified by microscopy, and the number of chloroplasts was determined by counting with a Neubauer chamber. An aliquot of 500-µL chloroplast suspension together with 100-µL 65% (v/v) HNO_3_ were pipetted into a 15-mL Falcon tube and digested overnight at 70°C. The ion concentrations were determined by ICP-MS using an iCAP-RQ ICP-MS instrument (Thermo Fisher Scientific, Bremen, Germany) fitted with a MicroFlow PFA-200 nebulizer and a Cetac ASX-560 (Teledyne, Cetac Technologies, Omaha, NE, USA) autosampler.

### Photosynthesis measurements

Plants were dark-adapted for 30 min, and all fluorescence measurements were performed on intact plants at room temperature. Images were captured, and maximal photochemical efficiency of PSII (*Fv/Fm*) and photochemical efficiency of PSII (Y (II)) changes with increasing light intensities were determined by IMAGING-PAM MAXI Version (IMAGING-PAM *M-Series*, Walz, Effeltrich, Germany). Saturating flashes (902 μmol m^−2^ s^−1^) were used to measure the maximum quantum yield of PSII. The photochemical efficiency of PSII was measured under a series of flashes with increasing intensity (1, 7, 21, 42, 69, 101, 140, 185, 237, 361, 512, 681, and 902 μmol m^−2^ s^−1^).

### Grafting

For reciprocal grafting of *bicat3-1* and Col-0 wild-type, stratified seeds were sown onto 1/2 MS plates, which were placed vertically in a growth cabinet set to 21°C day and 19°C night temperatures and 10-h light period with a light intensity of 150-µmol photons m^−2^ s^−1^ and grown for 5–6 days. Grafting was performed by using the Two Segment Shoot-Root Graft method according to Melnyk (2017) with some modifications. Sterile water was added to an empty petri dish containing two pieces of sterile Whatman 3MM Chr cellulose chromatography paper (WHA3030917, Whatman, Maidstone, UK) and one sterile Amersham Hybond-N^+^ membrane (RPN303B, Cytiva, Marlborough, MA, USA) strip on the top. One cotyledon of the seedling was cut off and discarded, and a transverse butt-end cut through the hypocotyl close to the shoot was made. Scions and rootstocks were grafted on the Hybond membrane strip, and the redundant water in the petri dish was removed by sterile Whatman paper strips. Three days after grafting, 1-mL sterile water was added to plates. One week after grafting, successfully grafted plants which were well attached and without adventitious roots were transferred to 1/2 MS plates containing 0.8% (w/v) agar and 0.25% (w/v) sucrose. After recovering for 1 week, well-developed grafted plants were transferred to hydroponic solution. Grafted plants grew in complete hydroponic basal media for 12 days and were cultured for two more weeks after transfer to treatment media. Plants were cultured as described above for hydroponic cultures.

### Leaf sectioning and cell size measurements

To visualize leaf flatness, leaves were embedded in 30-g L^−1^ low-melting agarose (Biozym Scientific, Hessisch Oldendorf, Germany); 40-μm sections were prepared using a vibrating microtome (Hyrax V 50, Carl Zeiss). To quantify leaf cell size and shape, leaf segments were fixed and embedded as described (Eschen-Lippold et al., 2012); semi-thin sections (1 µm) were transferred to glass slides and stained with 1-g L^−1^ toluidine blue. Images were taken by using an Axioskop 20 microscope (Carl Zeiss) equipped with an AxioCam MRc camera (Carl Zeiss). Morphometric measurements were performed with the iTEM software (Olympus SIS, Münster, Germany).

### Monosaccharide and sugar linkage determination

Plants were cultivated in a hydroponic system as described above. AIR was prepared from 18 to 25 mg of lyophilized tissue per sample essentially as previously described (Polko et al., 2018). In brief, the tissue was homogenized with a ball mill, sequentially washed with 70% (v/v) ethanol, chloroform: methanol (1:1 v/v), and acetone, followed by enzymatic de-starching. De-starched AIR polysaccharides were hydrolyzed and analyzed for monosaccharide composition via HPAEC-PAD as previously described (Voiniciuc and Günl, 2016), with the exact instrument and eluent gradient described by Mielke et al. (2021). Partially methylated alditol acetates of glycosidic linkages in 1 mg of the AIR material were derivatized and analyzed by GC–MS exactly as described in a recent publication (Robert et al., 2021).

### Callose detection

Callose depositions in leaves were analyzed by aniline blue staining according to Rissel et al. (2017) with slight modifications. Leaves were fixated and destained in 1:3 acetic acid/ethanol, washed in 150-mM K_2_HPO_4_ for 30 min, and subsequently incubated in Aniline blue solution (0.1 g L^−1^ Aniline blue [CI 42780, Carl Roth], 150-mM K_2_HPO_4_) in the dark for 3 h. Stained leaves were embedded in 50% (v/v) glycerol. Callose depositions were documented using a AxioCam MRm Rev. 3 camera mounted on an Axio Observer.Z1 (Carl Zeiss) microscope equipped with filter set 49 (DAPI, Carl Zeiss).

For ultrastructural localization of callose, the material was prepared, treated, and observed as described (Eschen-Lippold et al., 2012).

### Reciprocal crossing

Four- to five-week-old plants grown in the greenhouse were used for reciprocal crossing. Anthers were removed from the late unopened flowers, avoiding damage of the female reproductive part. Emasculated plants were cultivated overnight in the greenhouse for further maturation of stigmata. The stigmatic surfaces of emasculated flowers were pollinated with the desired pollen. Pollinated plants were further cultivated in the greenhouse until siliques were matured. Siliques were documented by SLR camera.

### Seed germination assay

Sterilized and stratified seeds were sown on 10 layers of sterilized wet Blue Roll paper and cultured in darkness at 22°C. Germinated seeds with emerged radicles were counted every 8 h.

### Pollen tube growth assays

For the in vivo assay, 4—to 5-week-old plants grown on soil in the greenhouse were used. Anthers were removed from late unopened flowers, avoiding damage of the female reproduction part. Emasculated plants were cultivated further overnight for maturation of the stigmata. The stigmatic surfaces of emasculated flowers were pollinated with desired pollen. Pistils were harvested 48 h after pollination and destained in 1:3 acetic acid/ethanol until they were transparent. Pistils were further incubated in 8-M NaOH overnight and subsequently stained with Aniline blue solution (0.1-g L^−1^ Aniline blue [CI 42780, Carl Roth], 150-mM K_2_HPO_4_) for 5 h in the dark after washing 3 times with 150-mM K_2_HPO_4_. Pictures were acquired with an LSM 880META with a Plan-apochromatic lens (20x/0.8) (Carl Zeiss) in tile scanning mode.

The *semi* in vivo pollen tube growth assay was performed as described (Dickinson et al., 2018). One hour after hand-pollination, pollinated pistils were excised above the junction to the ovary and subsequently placed on a gel pad in a humid chamber. Pictures were acquired with a SteREO Discovery V.20 stereomicroscope (Carl Zeiss).

For detection of pectin in pollen tubes, pollen were cultured in vitro for 6 h as described (Ischebeck et al., 2008). Pollen tubes were subsequently stained by 0.1 g L^−1^ Ruthenium Red (Sigma-Aldrich, St Louis, MO, USA) for 10 min. Pictures were acquired with an AxioCam MRc camera mounted on an Axioskop 20 microscope (Carl Zeiss).

### Immunostaining of pollen tubes

Freshly opened flowers from 4- to 5-week-old Arabidopsis plants grown on soil in the greenhouse were harvested and incubated in a humid box at 22°C for 30 min. Pollen was collected into filter-sterilized liquid pollen germination media (5-mM KCl, 1-mM MgSO_4_, 5-mM CaCl_2_, 0.1-g L^−1^ H_3_BO_3_, 100-g L^−1^ sucrose, pH 7.5) by moving flowers up and down in the medium. Pollen grains were incubated at 30°C for 40 min before further incubation at 22°C for 3 h. For immunostaining, pollen tubes were fixed in freshly prepared 30-g L^−1^ paraformaldehyde and 100-g L^−1^ sucrose in PIPES buffer (50-mM PIPES [pH 6.9], 2-mM EGTA, 2-mM MgSO_4_) at room temperature for 2 h. Pollen tubes were subsequently washed 2 times in PIPES buffer and 3 times in PBS (pH 7.2). For removing the residual paraformaldehyde, pollen tubes were incubated in 0.1-M NH_4_Cl PBS buffer for 5 min and once in PBS. For detection of low-esterified HG, pollen tubes were further incubated overnight in the 1:10-diluted primary antibody solution (JIM5, Plant Probes, University of Leeds, UK) in PBS buffer with 50-g L^−1^ BSA at 4°C after blocking in PBS buffer with 50-g L^−1^ BSA for 0.5 h. Pollen tubes were washed 4 times in PBS buffer with 1-g L^−1^ BSA, followed by 90-min incubation with 1:500 diluted secondary antibody solution (goat anti-rat IgG/Alexa Fluor 488 [A-11006, Thermo Fisher Scientific, Waltham, MA, USA]). Pollen tubes were subsequently washed 3 times with PBS buffer and transferred to microscopy slides. Fluorescence was observed by CLSM using a LSM 880META with a Plan-apochromatic lens (40×/0.95) (Carl Zeiss). Pictures of pollen tubes were taken in channel mode with the same gain and a resolution of 1,024 × 1,024. Excitation light source was a 488-nm multiline ion laser. Alexa Fluor 488 was detected by using a 493–598-nm band pass filter. Images were acquired by using the Zeiss ZEN blue software.

### Statistical analyses

Statistical analyses were performed with SigmaPlot version 13.0 (Systat, San Jose, CA, USA). The comparison of two groups was done by Student’s *t* test. Significance values are defined as ^*^P <0.05, ^**^P<0.01, and ^***^P<0.001. The number of replicates and the repetition of experiments is indicated in the figure legends.

### Accession numbers

The Arabidopsis Genome Initiative locus numbers for the genes mentioned in this article are as follows: BICAT3 (AT5G36290); ECA3 (AT1G10130).

## Supplemental data

The following materials are available in the online version of this article.

**Supplemental Figure S1.** Expression of *BICAT3:Venus* driven by the native *BICAT3* promoter complements the growth defect of the *bicat3-1* mutant under Mn^2+^ deficiency.

**Supplemental Figure S2.** BICAT3 does not co-localize with mitochondria and peroxisomes.

**Supplemental Figure S3.** GUS staining of *ProBICAT3-GUS* seedlings grown under different Mn^2+^ and Ca^2+^ supply levels.

**Supplemental Figure S4.** BICAT3 does not complement Fe^3+^-sensitive (*ccc1Δ*), Zn^2+^-sensitive (*zrc1Δ*), Cu^2+^-sensitive (*cup2Δ*), and Co^2+^-sensitive (*cot1Δ*) yeast strains.

**Supplemental Figure S5.** Characterization of *bicat3* mutants.

**Supplemental Figure S6.** *bicat3-1* shows retarded growth compared to the wild-type under Mn^2+^ deficiency.

**Supplemental Figure S7.** *bicat3-1* shows retarded root growth compared to the wild-type under Ca^2+^ toxicity.

**Supplemental Figure S8.** *bicat3-1* shows improved growth compared to the wild-type under Mn^2+^ toxicity.

**Supplemental Figure S9.** Growth of Col-0 and *bicat3-1* under different Ca^2+^ and Mn^2+^ supply.

**Supplemental Figure S10.** Fe^2+^, Zn^2+^, and Mg^2+^ concentrations of 5-week-old plants cultivated in hydroponics with 3.5 µM Mn^2+^ (control) or 0.05 µM Mn^2+^ (-Mn).

**Supplemental Figure S11.** Relative water content of shoots of 5-week-old Col-0, *bicat3-1*, and *bicat3-2* plants cultivated in hydroponics with 3.5 µM Mn^2+^ (control) or 0.05 µM Mn^2+^ (-Mn).

**Supplemental Figure S12.** Phenotypes of Col-0 and *bicat3-1* under different Mn^2+^ supply levels.

**Supplemental Figure S13.** Phenotypes of reciprocally grafted Col-0 and *bicat3-1* plants under control and Mn^2+^ deficiency (0.05 µM Mn^2+^) conditions.

**Supplemental Figure S14.** The cell size and shape of *bicat3-1* leaves changes under Mn^2+^ deficiency (0.05 µM Mn^2+^) compared to the wild-type.

**Supplemental Figure S15.** *bicat3-1* accumulates more callose in leaves compared to the wild-type under Mn^2+^ deficiency (0.05 µM Mn^2+^).

**Supplemental Figure S16.** *bicat3-1* accumulates more H_2_O_2_ in leaves compared to the wild-type under Mn^2+^ deficiency (0.05 µM Mn^2+^).

**Supplemental Figure S17.** Cell wall matrix sugar components in *bicat3* roots compared to the wild-type under Mn^2+^ deficiency (0.05 µM Mn^2+^) and control conditions.

**Supplemental Figure S18.** *bicat3-1* and *bicat3-2* mutants produce shorter siliques compared to the wild-type.

**Supplemental Figure S19.** The *bicat3-1* mutant produces larger seeds and germinates faster than the wild-type.

**Supplemental Figure S20.** Aniline blue staining of Col-0 and *bicat3-1* pollen tubes grown for 48 h in vivo.

**Supplemental Figure S21.** *Semi* in vivo pollen tube growth assay of Col-0, *bicat3-1*, *bicat3-2*, and *bica3-1* complemented by expression of *BICAT3* driven by its native promoter.

**Supplemental Figure S22.** *bicat3* mutant pollen tubes grow aberrantly in vitro and show abnormal pectin distribution.

**Supplemental Figure S23.** BICAT3 and ECA3 distinctly determine growth, Mn^2+^ and Ca^2+^ accumulation, and photosynthesis under Mn^2+^ limitation.

**Supplemental Figure S24.** Roots of *bicat3* mutants show no morphological defects under Mn^2+^ deficiency.

**Supplemental Table S1.** Glycosidic linkages of Col-0 and *bicat3* shoot and root cell wall AIR.

**Supplemental Table S2.** Primers and constructs used in this study.

**Supplemental Table S3.** Media used in this study.

## Supplementary Material

kiac387_Supplementary_Data

## Notes

E.P. and J.H. designed the research. J.H., B.Y., G.H., N.R., and T.P.V. performed research. M.S. and C.V. contributed new analytic tools. J.H., B.Y., G.H., N.R., C.V., and E.P. analyzed data; J.H. and E.P. wrote the article with contributions of all authors.

The author responsible for distribution of materials integral to the findings presented in this article in accordance with the policy described in the Instructions for Authors (https://academic.oup.com/plphys/pages/general-instructions) is: Edgar Peiter (edgar.peiter@landw.uni-halle.de).

## References

[kiac387-B1] Alejandro S , Cailliatte R, Alcon C, Dirick L, Domergue F, Correia D, Castaings L, Briat J-F, Mari S, Curie C (2017) Intracellular distribution of manganese by the *trans*-Golgi network transporter NRAMP2 is critical for photosynthesis and cellular redox homeostasis. Plant Cell 29: 3068–3084 29180598 10.1105/tpc.17.00578 PMC5757278

[kiac387-B2] Alejandro S , Höller S, Meier B, Peiter E (2020) Manganese in plants: from acquisition to subcellular allocation. Front Plant Sci 11: 300 32273877 10.3389/fpls.2020.00300 PMC7113377

[kiac387-B3] Altartouri B , Bidhendi AJ, Tani T, Suzuki J, Conrad C, Chebli Y, Liu N, Karunakaran C, Scarcelli G, Geitmann A (2019) Pectin chemistry and cellulose crystallinity govern pavement cell morphogenesis in a multi-step mechanism. Plant Physiol 181: 127–141 31363005 10.1104/pp.19.00303 PMC6716242

[kiac387-B4] Amos RA , Pattathil S, Yang JY, Atmodjo MA, Urbanowicz BR, Moremen KW, Mohnen D (2018) A two-phase model for the non-processive biosynthesis of homogalacturonan polysaccharides by the GAUT1:GAUT7 complex. J Biol Chem 293: 19047–19063 30327429 10.1074/jbc.RA118.004463 PMC6295712

[kiac387-B5] Amsbury S , Hunt L, Elhaddad N, Baillie A, Lundgren M, Verhertbruggen Y, Scheller HV, Knox JP, Fleming AJ, Gray JE (2016) Stomatal function requires pectin de-methyl-esterification of the guard cell wall. Curr Biol 26: 2899–2906 27720618 10.1016/j.cub.2016.08.021 PMC5106435

[kiac387-B6] Andresen E , Peiter E, Küpper H (2018) Trace metal metabolism in plants. J Exp Bot 69: 909–954 29447378 10.1093/jxb/erx465

[kiac387-B7] Atmodjo MA , Sakuragi Y, Zhu X, Burrell AJ, Mohanty SS, Atwood JA, Orlando R, Scheller HV, Mohnen D (2011) Galacturonosyltransferase (GAUT)1 and GAUT7 are the core of a plant cell wall pectin biosynthetic homogalacturonan: galacturonosyltransferase complex. Proc Natl Acad Sci USA 108: 20225–20230 22135470 10.1073/pnas.1112816108 PMC3250160

[kiac387-B8] Bacete L , Mélida H, Miedes E, Molina A (2018) Plant cell wall-mediated immunity: cell wall changes trigger disease resistance responses. Plant J 93: 614–636 29266460 10.1111/tpj.13807

[kiac387-B9] Basu D , Tian L, Wang W, Bobbs S, Herock H, Travers A, Showalter AM (2015) A small multigene hydroxyproline-O-galactosyltransferase family functions in arabinogalactan-protein glycosylation, growth and development in Arabidopsis. BMC Plant Biol 15: 295 26690932 10.1186/s12870-015-0670-7 PMC4687291

[kiac387-B10] Basu D , Wang W, Ma S, DeBrosse T, Poirier E, Emch K, Soukup E, Tian L, Showalter AM (2015) Two hydroxyproline galactosyltransferases, GALT5 and GALT2, function in arabinogalactan-protein glycosylation, growth and development in Arabidopsis. PLoS One 10: e0125624 25974423 10.1371/journal.pone.0125624 PMC4431829

[kiac387-B11] Bouton S , Leboeuf E, Mouille G, Leydecker M-T, Talbotec J, Granier F, Lahaye M, Höfte H, Truong H-N (2002) *QUASIMODO1* encodes a putative membrane-bound glycosyltransferase required for normal pectin synthesis and cell adhesion in Arabidopsis. Plant Cell 14: 2577–2590 12368506 10.1105/tpc.004259 PMC151237

[kiac387-B12] Chebli Y , Kaneda M, Zerzour R, Geitmann A (2012) The cell wall of the Arabidopsis pollen tube-spatial distribution, recycling, and network formation of polysaccharides. Plant Physiol 160: 1940–1955 23037507 10.1104/pp.112.199729 PMC3510122

[kiac387-B13] Clough SJ , Bent AF (1998) Floral dip: a simplified method for *Agrobacterium*-mediated transformation of *Arabidopsis thaliana*. Plant J 16: 735–743 10069079 10.1046/j.1365-313x.1998.00343.x

[kiac387-B14] Colinet AS , Sengottaiyan P, Deschamps A, Colsoul M-L, Thines L, Demaegd D, Duchene M-C, Foulquier F, Hols P, Morsomme P (2016) Yeast Gdt1 is a Golgi-localized calcium transporter required for stress-induced calcium signaling and protein glycosylation. Sci Rep 6: 24282 27075443 10.1038/srep24282 PMC4830978

[kiac387-B15] Cosgrove DJ (2016) Plant cell wall extensibility: connecting plant cell growth with cell wall structure, mechanics, and the action of wall-modifying enzymes. J Exp Bot 67: 463–476 26608646 10.1093/jxb/erv511

[kiac387-B16] Cosgrove DJ (2018) Diffuse growth of plant cell walls. Plant Physiol 176: 16–27 29138349 10.1104/pp.17.01541 PMC5761826

[kiac387-B17] Culbertson AT , Ehrlich JJ, Choe J-Y, Honzatko RB, Zabotina OA (2018) Structure of xyloglucan xylosyltransferase 1 reveals simple steric rules that define biological patterns of xyloglucan polymers. Proc Natl Acad Sci USA 115: 6064–6069 29784804 10.1073/pnas.1801105115 PMC6003343

[kiac387-B18] Culbertson AT , Tietze AA, Tietze D, Chou Y-H, Smith AL, Young ZT, Zabotina OA (2016) A homology model of Xyloglucan Xylosyltransferase 2 reveals critical amino acids involved in substrate binding. Glycobiology 26: 961–972 27146522 10.1093/glycob/cww050

[kiac387-B19] Dardelle F , Lehner A, Ramdani Y, Bardor M, Lerouge P, Driouich A, Mollet JC (2010) Biochemical and immunocytological characterizations of Arabidopsis pollen tube cell wall. Plant Physiol 153: 1563–1576 20547702 10.1104/pp.110.158881 PMC2923879

[kiac387-B20] De Lorenzo G , Ferrari S, Giovannoni M, Mattei B, Cervone F (2019) Cell wall traits that influence plant development, immunity, and bioconversion. Plant J 97: 134–147 30548980 10.1111/tpj.14196

[kiac387-B21] De Storme N , Geelen D (2014) Callose homeostasis at plasmodesmata: molecular regulators and developmental relevance. Front Plant Sci 5: 138 24795733 10.3389/fpls.2014.00138 PMC4001042

[kiac387-B22] Dehors J , Mareck A, Kiefer-Meyer M-C, Menu-Bouaouiche L, Lehner A, Mollet J-C (2019) Evolution of cell wall polymers in tip-growing land plant gametophytes: composition, distribution, functional aspects and their remodeling. Front Plant Sci 10: 441 31057570 10.3389/fpls.2019.00441 PMC6482432

[kiac387-B23] Delhaize E , Gruber BD, Pittman JK, White RG, Leung H, Miao Y, Jiang L, Ryan PR, Richardson AE (2007) A role for the *AtMTP11* gene of Arabidopsis in manganese transport and tolerance. Plant J 51: 198–210 17559518 10.1111/j.1365-313X.2007.03138.x

[kiac387-B24] Demaegd D , Colinet A-S, Deschamps A, Morsomme P (2014) Molecular evolution of a novel family of putative calcium transporters. PLoS One 9: e100851 24955841 10.1371/journal.pone.0100851 PMC4067407

[kiac387-B25] Demaegd D , Foulquier F, Colinet AS, Gremillon L, Legrand D, Mariot P, Peiter E, Van Schaftingen E, Matthijs G, Morsomme P (2013) Newly characterized Golgi-localized family of proteins is involved in calcium and pH homeostasis in yeast and human cells. Proc Natl Acad Sci USA 110: 6859–6864 23569283 10.1073/pnas.1219871110 PMC3637739

[kiac387-B26] Dickinson H , Rodriguez-Enriquez J, Grant-Downton R (2018) Pollen germination and pollen tube growth of *Arabidopsis thaliana*: *in vitro* and semi *in vivo* methods. Bio-protocol 8: e2977 34395777 10.21769/BioProtoc.2977 PMC8328669

[kiac387-B27] Donohoe BS , Kang B-H, Gerl MJ, Gergely ZR, McMichael CM, Bednarek SY, Staehelin LA (2013) *Cis*-Golgi cisternal assembly and biosynthetic activation occur sequentially in plants and algae. Traffic 14: 551–567 23369235 10.1111/tra.12052 PMC3622843

[kiac387-B28] Duan Q , Liu MCJ, Kita D, Jordan SS, Yeh FLJ, Yvon R, Carpenter H, Federico AN, Garcia-Valencia LE, Eyles SJ, et al (2020) FERONIA controls pectin- and nitric oxide-mediated male-female interaction. Nature 579: 561–566 32214247 10.1038/s41586-020-2106-2

[kiac387-B29] Dulary E , Yu SY, Houdo M, de Bettignies G, Decool V, Potelle S, Duvet S, Krzewinski-Recchi M-A, Garat A, Matthijs G, et al (2018) Investigating the function of Gdt1p in yeast Golgi glycosylation. Biochim Biophys Act 1862: 394–402 10.1016/j.bbagen.2017.11.006 29108953

[kiac387-B30] Ebert B , Birdseye D, Liwanag AJM, Laursen T, Rennie EA, Guo XY, Catena M, Rautengarten C, Stonebloom SH, Gluza P, et al (2018) The three members of the Arabidopsis glycosyltransferase family 92 are functional β-1,4-galactan synthases. Plant Cell Physiol 59: 2624–2636 30184190 10.1093/pcp/pcy180

[kiac387-B31] Eisenhut M , Hoecker N, Schmidt SB, Basgaran RM, Flachbart S, Jahns P, Eser T, Geimer S, Husted S, Webers APM, et al (2018) The plastid envelope CHLOROPLAST MANGANESE TRANSPORTER1 is essential for manganese homeostasis in Arabidopsis. Mol Plant 11: 955–969 29734002 10.1016/j.molp.2018.04.008

[kiac387-B32] Eroglu S , Giehl RFH, Meier B, Takahashi M, Terada Y, Ignatyev K, Andresen E, Küpper H, Peiter E, von Wirén N (2017) Metal Tolerance Protein 8 mediates manganese homeostasis and iron re-allocation during seed development and germination. Plant Physiol 174: 1633–1647 28461400 10.1104/pp.16.01646 PMC5490884

[kiac387-B33] Eroglu S , Meier B, von Wirén N, Peiter E (2016) The vacuolar manganese transporter MTP8 determines tolerance to iron deficiency-induced chlorosis in Arabidopsis. Plant Physiol 170: 1030–1045 26668333 10.1104/pp.15.01194 PMC4734556

[kiac387-B34] Eschen-Lippold L , Landgraf R, Smolka U, Schulze S, Heilmann M, Heilmann I, Hause G, Rosahl S (2012) Activation of defense against *Phytophthora infestans* in potato by down-regulation of syntaxin gene expression. New Phytol 193: 985–996 22243492 10.1111/j.1469-8137.2011.04024.x

[kiac387-B35] Foulquier F , Amyere M, Jaeken J, Zeevaert R, Schollen E, Race V, Bammens R, Morelle W, Rosnoblet C, Legrand D, et al (2012) TMEM165 deficiency causes a congenital disorder of glycosylation. Am J Hum Genet 91: 15–26 22683087 10.1016/j.ajhg.2012.05.002 PMC3397274

[kiac387-B36] Foulquier F , Legrand D (2020) Biometals and glycosylation in humans: congenital disorders of glycosylation shed lights into the crucial role of Golgi manganese homeostasis. Biochim Biophys Act 1864: 129674 10.1016/j.bbagen.2020.129674 32599014

[kiac387-B37] Franck CM , Westermann J, Boisson-Dernier A (2018) Plant malectin-like receptor kinases: from cell wall integrity to immunity and beyond. Annu Rev Plant Biol 69: 301–328 29539271 10.1146/annurev-arplant-042817-040557

[kiac387-B38] Frank J , Happeck R, Meier B, Hoang MTT, Stribny J, Hause G, Ding H, Morsomme P, Baginsky S, Peiter E (2019) Chloroplast-localized BICAT proteins shape stromal calcium signals and are required for efficient photosynthesis. New Phytol 221: 866–880 30169890 10.1111/nph.15407

[kiac387-B39] Haas KT , Wightman R, Meyerowitz EM, Peaucelle A (2020) Pectin homogalacturonan nanofilament expansion drives morphogenesis in plant epidermal cells. Science 367: 1003–1007 32108107 10.1126/science.aaz5103 PMC7932746

[kiac387-B40] Hawes C , Satiat-Jeunemaitre B (2005) The plant Golgi apparatus - going with the flow. Biochim Biophys Act 1744: 93–107 10.1016/j.bbamcr.2005.03.009 15922463

[kiac387-B41] He J , Rössner N, Hoang MTT, Alejandro S, Peiter E (2021) Transport, functions, and interaction of calcium and manganese in plant organellar compartments. Plant Physiol 187: 1940-1972 35235665 10.1093/plphys/kiab122 PMC8890496

[kiac387-B42] Hepler PK , Rounds CM, Winship LJ (2013) Control of cell wall extensibility during pollen tube growth. Mol Plant 6: 998–1017 23770837 10.1093/mp/sst103 PMC4043104

[kiac387-B43] Höller S , Küpper H, Brückner D, Garrevoet J, Spiers K, Falkenberg G, Andresen E, Peiter E (2022) Overexpression of *METAL TOLERANCE PROTEIN8* reveals new aspects of metal transport in *Arabidopsis thaliana* seeds. Plant Biol 24: 23–29 34546650 10.1111/plb.13342

[kiac387-B44] Houdou M , Lebredonchel E, Garat A, Duvet S, Legrand D, Decool V, Klein A, Ouzzine M, Gasnier B, Potelle S, et al (2019) Involvement of thapsigargin- and cyclopiazonic acid-sensitive pumps in the rescue of TMEM165-associated glycosylation defects by Mn^2^^+^. FASEB J 33: 2669–2679 30307768 10.1096/fj.201800387R

[kiac387-B45] Huby E , Napier JA, Baillieul F, Michaelson LV, Dhondt-Cordelier S (2020) Sphingolipids: towards an integrated view of metabolism during the plant stress response. New Phytol 225: 659–670 31211869 10.1111/nph.15997 PMC6973233

[kiac387-B46] Hunter K , Kimura S, Rokka A, Tran HC, Toyota M, Kukkonen JP, Wrzaczek M (2019) CRK2 enhances salt tolerance by regulating callose deposition in connection with PLDα1. Plant Physiol 180: 2004–2021 31118265 10.1104/pp.19.00560 PMC6670071

[kiac387-B47] Ischebeck T , Stenzel I, Heilmann I (2008) Type B phosphatidylinositol-4-phosphate 5-kinases mediate *Arabidopsis* and *Nicotiana tabacum* pollen tube growth by regulating apical pectin secretion. Plant Cell 20: 3312–3330 19060112 10.1105/tpc.108.059568 PMC2630452

[kiac387-B49] Kim SJ , Held MA, Zemelis S, Wilkerson C, Brandizzi F (2015) CGR2 and CGR3 have critical overlapping roles in pectin methylesterification and plant growth in *Arabidopsis thaliana*. Plant J 82: 208–220 25704846 10.1111/tpj.12802

[kiac387-B50] Klaassen MT , Trindade LM (2020) RG-I galactan side-chains are involved in the regulation of the water-binding capacity of potato cell walls. Carbohydr Polym 227: 115353 31590885 10.1016/j.carbpol.2019.115353

[kiac387-B51] Kobayashi K (2016) Role of membrane glycerolipids in photosynthesis, thylakoid biogenesis and chloroplast development. J Plant Res 129: 565–580 27114097 10.1007/s10265-016-0827-y PMC5897459

[kiac387-B52] Kohorn BD , Kohorn SL (2012) The cell wall-associated kinases, WAKs, as pectin receptors. Front Plant Sci 3: 88 22639672 10.3389/fpls.2012.00088 PMC3355716

[kiac387-B53] Lamport DTA , Tan L, Held MA, Kieliszewski MJ (2018) Pollen tube growth and guidance: occam’s razor sharpened on a molecular arabinogalactan glycoprotein Rosetta Stone. New Phytol 217: 491–500 28990197 10.1111/nph.14845

[kiac387-B54] Laursen T , Stonebloom SH, Pidatala VR, Birdseye DS, Clausen MH, Mortimer JC, Scheller HV (2018) Bifunctional glycosyltransferases catalyze both extension and termination of pectic galactan oligosaccharides. Plant J 94: 340–351 29418030 10.1111/tpj.13860

[kiac387-B55] Li X , Chanroj S, Wu Z, Romanowsky SM, Harper JF, Sze H (2008) A distinct endosomal Ca^2+^/Mn^2+^ pump affects root growth through the secretory process. Plant Physiol 147: 1675–1689 18567829 10.1104/pp.108.119909 PMC2492598

[kiac387-B56] Liang F , Cunningham KW, Harper JF, Sze H (1997) ECA1 complements yeast mutants defective in Ca^2+^ pumps and encodes an endoplasmic reticulum-type Ca^2+^-ATPase in *Arabidopsis thaliana*. Proc Natl Acad Sci USA 94: 8579–8584 9238019 10.1073/pnas.94.16.8579 PMC23025

[kiac387-B57] Liu XL , Liu LF, Niu QK, Xia CA, Yang KZ, Li R, Chen LQ, Zhang XQ, Zhou YH, Ye D (2011) MALE GAMETOPHYTE DEFECTIVE 4 encodes a rhamnogalacturonan II xylosyltransferase and is important for growth of pollen tubes and roots in Arabidopsis. Plant J 65: 647–660 21288267 10.1111/j.1365-313X.2010.04452.x

[kiac387-B58] Lopez-Hernandez F , Tryfona T, Rizza A, Yu XL, Harris MOB, Webb AAR, Kotake T, Dupree P (2020) Calcium binding by arabinogalactan polysaccharides is important for normal plant development. Plant Cell 32: 3346–3369 32769130 10.1105/tpc.20.00027 PMC7534474

[kiac387-B59] Lund CH , Stenbaek A, Atmodjo MA, Rasmussen RE, Moller IE, Erstad SM, Biswal AK, Mohnen D, Mravec J, Sakuragi Y (2020) Pectin synthesis and pollen tube growth in *Arabidopsis* involves three GAUT1 Golgi-anchoring proteins: GAUT5, GAUT6, and GAUT7. Front Plant Sci 11: 585774 33072156 10.3389/fpls.2020.585774 PMC7533613

[kiac387-B60] Melnyk CW (2017) Grafting with *Arabidopsis thaliana*. Methods Mol Biol 1497: 9–18 27864753 10.1007/978-1-4939-6469-7_2

[kiac387-B61] Mielke S , Zimmer M, Meena MK, Dreos R, Stellmach H, Hause B, Voiniciuc C, Gasperini D (2021) Jasmonate biosynthesis arising from altered cell walls is prompted by turgor-driven mechanical compression. Sci Adv 7: eabf0356 33568489 10.1126/sciadv.abf0356 PMC7875531

[kiac387-B62] Mills RF , Doherty ML, López-Marqués RL, Weimar T, Dupree P, Palmgren MG, Pittman JK, Williams LE (2008) ECA3, a Golgi-localized P_2A_-type ATPase, plays a crucial role in manganese nutrition in Arabidopsis. Plant Physiol 146: 116–128 18024560 10.1104/pp.107.110817 PMC2230566

[kiac387-B63] Molina A , Miedes E, Bacete L, Rodríguez T, Mélida H, Denancé N, Sánchez-Vallet A, Rivière MP, López G, Freydier A, et al (2021) *Arabidopsis* cell wall composition determines disease resistance specificity and fitness. Proc Natl Acad Sci USA 118: e2010243118 33509925 10.1073/pnas.2010243118 PMC7865177

[kiac387-B64] Mollet JC , Leroux C, Dardelle F, Lehner A (2013) Cell wall composition, biosynthesis and remodeling during pollen tube growth. Plants 2: 107–147 27137369 10.3390/plants2010107 PMC4844286

[kiac387-B65] Mortimer JC , Scheller HV (2020) Synthesis and function of complex sphingolipid glycosylation. Trends Plant Sci 25: 522–524 32407692 10.1016/j.tplants.2020.03.007

[kiac387-B66] Nagashima Y , von Schaewen A, Koiwa H (2018) Function of N-glycosylation in plants. Plant Sci 274: 70–79 30080642 10.1016/j.plantsci.2018.05.007

[kiac387-B67] Nelson BK , Cai X, Nebenführ A (2007) A multicolored set of in *vivo* organelle markers for co-localization studies in Arabidopsis and other plants. Plant J 51: 1126–1136 17666025 10.1111/j.1365-313X.2007.03212.x

[kiac387-B68] Nour-Eldin HH , Hansen BG, Norholm MHH, Jensen JK, Halkier BA (2006) Advancing uracil-excision based cloning towards an ideal technique for cloning PCR fragments. Nucl Acid Res 34: e122 10.1093/nar/gkl635 PMC1635280 17000637

[kiac387-B69] Ogawa-Ohnishi M , Matsubayashi Y (2015) Identification of three potent hydroxyproline *O*-galactosyltransferases in Arabidopsis. Plant J 81: 736–746 25600942 10.1111/tpj.12764

[kiac387-B70] Ogawa-Ohnishi M , Matsushita W, Matsubayashi Y (2013) Identification of three hydroxyproline O-arabinosyltransferases in *Arabidopsis thaliana*. Nature Chem Biol 9: 726–730 24036508 10.1038/nchembio.1351

[kiac387-B71] Park YB , Cosgrove DJ (2012) Changes in cell wall biomechanical properties in the xyloglucan-deficient *xxt1/xxt2* mutant of Arabidopsis. Plant Physiol 158: 465–475 22108526 10.1104/pp.111.189779 PMC3252101

[kiac387-B72] Peaucelle A , Braybrook SA, Le Guillou L, Bron E, Kuhlemeier C, Höfte H (2011) Pectin-induced changes in cell wall mechanics underlie organ initiation in *Arabidopsis*. Curr Biol 21: 1720–1726 21982593 10.1016/j.cub.2011.08.057

[kiac387-B73] Peiter E , Montanini B, Gobert A, Pedas P, Husted S, Maathuis FJM, Blaudez D, Chalot M, Sanders D (2007) A secretory pathway-localized cation diffusion facilitator confers plant manganese tolerance. Proc Natl Acad Sci USA 104: 8532–8537 17494768 10.1073/pnas.0609507104 PMC1895984

[kiac387-B74] Pillai SE , Kumar C, Patel HK, Sonti RV (2018) Overexpression of a cell wall damage induced transcription factor, OsWRKY42, leads to enhanced callose deposition and tolerance to salt stress but does not enhance tolerance to bacterial infection. BMC Plant Biol 18: 177 30176792 10.1186/s12870-018-1391-5 PMC6122458

[kiac387-B75] Polko JK , Barnes WJ, Voiniciuc C, Doctor S, Steinwand B, Hill JL Jr., Tien M, Pauly M, Anderson CT, Kieber JJ (2018) SHOU4 proteins regulate trafficking of cellulose synthase complexes to the plasma membrane. Curr Biol 28: 3174–3182 30245104 10.1016/j.cub.2018.07.076

[kiac387-B76] Potelle S , Morelle W, Dulary E, Duvet S, Vicogne D, Spriet C, Krzewinski-Recchi MA, Morsomme P, Jaeken J, Matthijs G, et al (2016) Glycosylation abnormalities in Gdt1p/TMEM165 deficient cells result from a defect in Golgi manganese homeostasis. Hum Mol Genet 25: 1489–1500 27008884 10.1093/hmg/ddw026

[kiac387-B77] Qin Y , Leydon AR, Manziello A, Pandey R, Mount D, Denic S, Vasic B, Johnson MA, Palanivelu R (2009) Penetration of the stigma and style elicits a novel transcriptome in pollen tubes, pointing to genes critical for growth in a pistil. PLoS Genet 5: e1000621 19714218 10.1371/journal.pgen.1000621 PMC2726614

[kiac387-B78] Rissel D , Heym PP, Thor K, Brandt W, Wessjohann LA, Peiter E (2017) No silver bullet - Canonical poly(ADP-ribose) polymerases (PARPs) are no universal factors of abiotic and biotic stress resistance of *Arabidopsis thaliana*. Front Plant Sci 8: 59 28220129 10.3389/fpls.2017.00059 PMC5292411

[kiac387-B79] Robert M , Waldhauer J, Stritt F, Yang B, Pauly M, Voiniciuc C (2021) Modular biosynthesis of plant hemicellulose and its impact on yeast cells. Biotechnol Biofuels 14: 140 34147122 10.1186/s13068-021-01985-z PMC8214268

[kiac387-B80] Rui Y , Anderson CT (2016) Functional analysis of cellulose and xyloglucan in the walls of stomatal guard cells of Arabidopsis. Plant Physiol 170: 1398–1419 26729799 10.1104/pp.15.01066 PMC4775103

[kiac387-B81] Rui Y , Chen Y, Yi H, Purzycki T, Puri VM, Anderson CT (2019) Synergistic pectin degradation and guard cell pressurization underlie stomatal pore formation. Plant Physiol 180: 66–77 30804009 10.1104/pp.19.00135 PMC6501081

[kiac387-B82] Rui Y , Dinneny JR (2020) A wall with integrity: surveillance and maintenance of the plant cell wall under stress. New Phytol 225: 1428–1439 31486535 10.1111/nph.16166

[kiac387-B83] Schneider A , Steinberger I, Herdean A, Gandini C, Eisenhut M, Kurz S, Morper A, Hoecker N, Ruhle T, Labs M, et al (2016) The evolutionarily conserved protein PHOTOSYNTHESIS AFFECTED MUTANT71 is required for efficient manganese uptake at the thylakoid membrane in Arabidopsis. Plant Cell 28: 892–910 27020959 10.1105/tpc.15.00812 PMC4863382

[kiac387-B84] Seifert GJ (2020) On the potential function of type II arabinogalactan *O*-glycosylation in regulating the fate of plant secretory proteins. Front Plant Sci 11: 563735 33013983 10.3389/fpls.2020.563735 PMC7511660

[kiac387-B85] Shigaki T , Pittman JK, Hirschi KD (2003) Manganese specificity determinants in the *Arabidopsis* metal/H^+^ antiporter CAX2. J Biol Chem 278: 6610–6617 12496310 10.1074/jbc.M209952200

[kiac387-B86] Shikanai Y , Yoshida R, Hirano T, Enomoto Y, Li B, Asada M, Yamagami M, Yamaguchi K, Shigenobu S, Tabata R, et al (2020) Callose synthesis suppresses cell death induced by low-calcium conditions in leaves. Plant Physiol 182: 2199–2212 32024698 10.1104/pp.19.00784 PMC7140939

[kiac387-B87] Silva J , Ferraz R, Dupree P, Showalter AM, Coimbra S (2020) Three decades of advances in arabinogalactan-protein biosynthesis. Front Plant Sci 11: 610377 33384708 10.3389/fpls.2020.610377 PMC7769824

[kiac387-B88] Stribny J , Thines L, Deschamps A, Goffin P, Morsomme P (2020) The human Golgi protein TMEM165 transports calcium and manganese in yeast and bacterial cells. J Biol Chem 295: 3865–3874 32047108 10.1074/jbc.RA119.012249 PMC7086029

[kiac387-B89] Tabassum N , Eschen-Lippold L, Athmer B, Baruah M, Brode M, Maldonado-Bonilla LD, Hoehenwarter W, Hause G, Scheel D, Lee J (2020) Phosphorylation-dependent control of an RNA granule-localized protein that fine-tunes defence gene expression at a post-transcriptional level. Plant J 101: 1023–1039 31628867 10.1111/tpj.14573

[kiac387-B90] Thines L , Deschamps A, Sengottaiyan P, Savel O, Stribny J, Morsomme P (2018) The yeast protein Gdt1p transports Mn^2+^ ions and thereby regulates manganese homeostasis in the Golgi. J Biol Chem 293: 8048–8055 29632074 10.1074/jbc.RA118.002324 PMC5971465

[kiac387-B91] Thines L , Deschamps A, Stribny J, Morsomme P (2019) Yeast as a tool for deeper understanding of human manganese-related diseases. Genes 10: 545 31319631 10.3390/genes10070545 PMC6678438

[kiac387-B92] Thomine S , Lelièvre F, Debarbieux E, Schroeder JI, Barbier-Brygoo H (2003) AtNRAMP3, a multispecific vacuolar metal transporter involved in plant responses to iron deficiency. Plant J 34: 685–695 12787249 10.1046/j.1365-313x.2003.01760.x

[kiac387-B93] Ülker B , Peiter E, Dixon DP, Moffat C, Capper R, Bouché N, Edwards R, Sanders D, Knight H, Knight MR (2008) Getting the most out of publicly available T-DNA insertion lines. Plant J 56: 665–677 18644000 10.1111/j.1365-313X.2008.03608.x

[kiac387-B94] Voiniciuc C , Günl M (2016) Analysis of monosaccharides in total mucilage extractable from *Arabidopsis* seeds. Bio-protocol 6: e1801

[kiac387-B95] Wagner TA , Kohorn BD (2001) Wall-associated kinases are expressed throughout plant development and are required for cell expansion. Plant Cell 13: 303–318 11226187 10.1105/tpc.13.2.303 PMC102244

[kiac387-B96] Wang C , Xu W, Jin H, Zhang T, Lai J, Zhou X, Zhang S, Liu S, Duan X, Wang H, et al (2016) A putative chloroplast-localized Ca^2+^/H^+^ antiporter CCHA1 is involved in calcium and pH homeostasis and required for PSII function in *Arabidopsis*. Mol Plant 9: 1183–1196 27302341 10.1016/j.molp.2016.05.015

[kiac387-B97] Wang L , Wang W, Wang YQ, Liu YY, Wang JX, Zhang XQ, Ye D, Chen LQ (2013) Arabidopsis galacturonosyltransferase (GAUT) 13 and GAUT14 have redundant functions in pollen tube growth. Mol Plant 6: 1131–1148 23709340 10.1093/mp/sst084

[kiac387-B98] Wang Y , Li X, Fan B, Zhu C, Chen Z (2021) Regulation and function of defense-related callose deposition in plants. Int J Mol Sci 22: 2393 33673633 10.3390/ijms22052393 PMC7957820

[kiac387-B99] Wee EGT , Sherrier DJ, Prime TA, Dupree P (1998) Targeting of active sialyltransferase to the plant Golgi apparatus. Plant Cell 10: 1759–1768 9761801 10.1105/tpc.10.10.1759 PMC143948

[kiac387-B100] Wei X , Guo J, Li M, Liu Z (2015) Structural mechanism underlying the specific recognition between the Arabidopsis state-transition phosphatase TAP38/PPH1 and phosphorylated light-harvesting complex protein Lhcb1. Plant Cell 27: 1113–1127 25888588 10.1105/tpc.15.00102 PMC4558704

[kiac387-B101] Wu HC , Bulgakov VP, Jinn TL (2018) Pectin methylesterases: cell wall remodeling proteins are required for plant response to heat stress. Front Plant Sci 9: 1612 30459794 10.3389/fpls.2018.01612 PMC6232315

[kiac387-B102] Wu Z , Liang F, Hong B, Young JC, Sussman MR, Harper JF, Sze H (2002) An endoplasmic reticulum-bound Ca^2+^/Mn^2+^ pump, ECA1, supports plant growth and confers tolerance to Mn^2+^ stress. Plant Physiol 130: 128–137 12226493 10.1104/pp.004440 PMC166546

[kiac387-B103] Yang CH , Wang C, Singh S, Fan N, Liu S, Zhao L, Cao H, Xie W, Yang C, Huang CF (2021) Golgi-localised manganese transporter PML3 regulates *Arabidopsis* growth through modulating Golgi glycosylation and cell wall biosynthesis. New Phytol 231: 2200–2214 33454966 10.1111/nph.17209

[kiac387-B104] Zhang B , Zhang C, Liu C, Fu A, Luan S (2021) A Golgi-localized manganese transporter functions in pollen tube tip growth to control male fertility in *Arabidopsis*. Plant Commun 2: 100178 34027392 10.1016/j.xplc.2021.100178 PMC8132125

[kiac387-B105] Zhang B , Zhang C, Liu C, Jing Y, Wang Y, Jin L, Yang L, Fu A, Shi J, Zhao F, et al (2018) Inner envelope CHLOROPLAST MANGANESE TRANSPORTER 1 supports manganese homeostasis and phototrophic growth in *Arabidopsis*. Mol Plant 11: 943–954 29734003 10.1016/j.molp.2018.04.007

[kiac387-B106] Zhang GF , Staehelin LA (1992) Functional compartmentation of the Golgi apparatus of plant cells. Immunocytochemical analysis of high-pressure frozen- and freeze-substituted sycamore maple suspension culture cells. Plant Physiol 99: 1070–1083 16668973 10.1104/pp.99.3.1070 PMC1080586

[kiac387-B107] Zhao F , Chen W, Sechet J, Martin M, Bovio S, Lionnet C, Long Y, Battu V, Mouille G, Monéger F, et al (2019) Xyloglucans and microtubules synergistically maintain meristem geometry and phyllotaxis. Plant Physiol 181: 1191–1206 31537749 10.1104/pp.19.00608 PMC6836833

[kiac387-B108] Zykwinska A , Thibault JF, Ralet MC (2007) Organization of pectic arabinan and galactan side chains in association with cellulose microfibrils in primary cell walls and related models envisaged. J Exp Bot 58: 1795–1802 17383990 10.1093/jxb/erm037

