# Supplementary material for: The trans-Golgi-localized protein BICAT3 regulates manganese allocation and matrix polysaccharide biosynthesis
Source: Plant Physiol. 2022 Aug 22;190(4):2579–600. doi: 10.1093/plphys/kiac387 (PMC9706472; doi:10.1093/plphys/kiac387)
Supplement: kiac387_Supplementary_Data [file kiac387_supplementary_data.pdf]

Supplemental Data

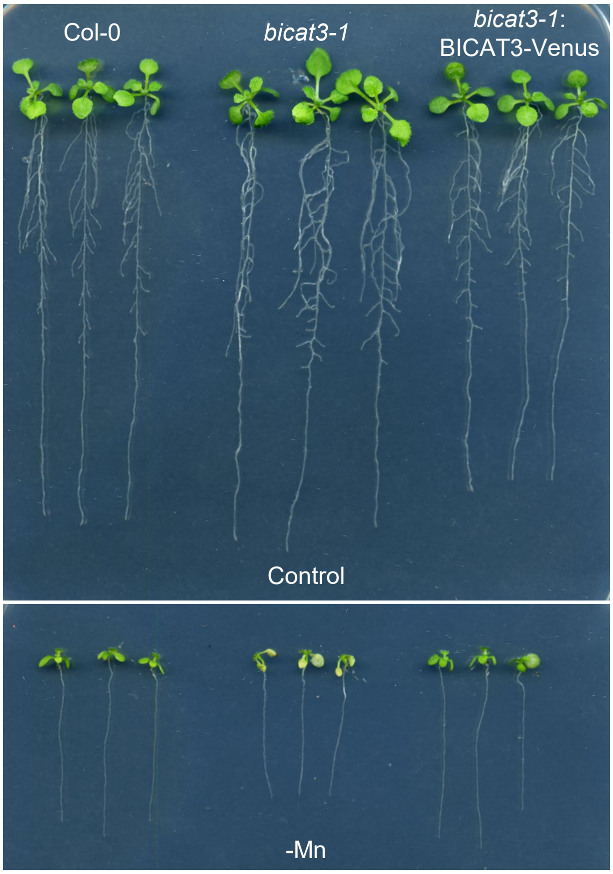

Supplemental Figure S1. Expression of *BICAT3:Venus* driven by the native *BICAT3* promoter complements the growth defect of the *bicat3-1* mutant under Mn deficiency.
The experiment was repeated twice with similar results.

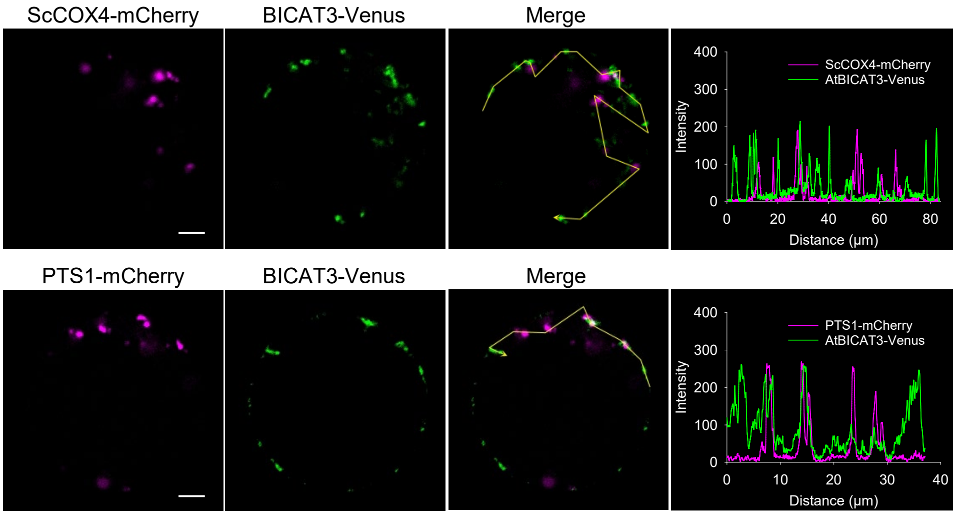

Supplemental Figure S2. BICAT3 does not co-localize with mitochondria and peroxisomes. *BICAT3-Venus* was co-expressed with a mitochondria marker (*ScCOX4*) and a peroxisome marker (*PTS1*) in Arabidopsis mesophyll protoplasts. Scale bars represent 5 µm.

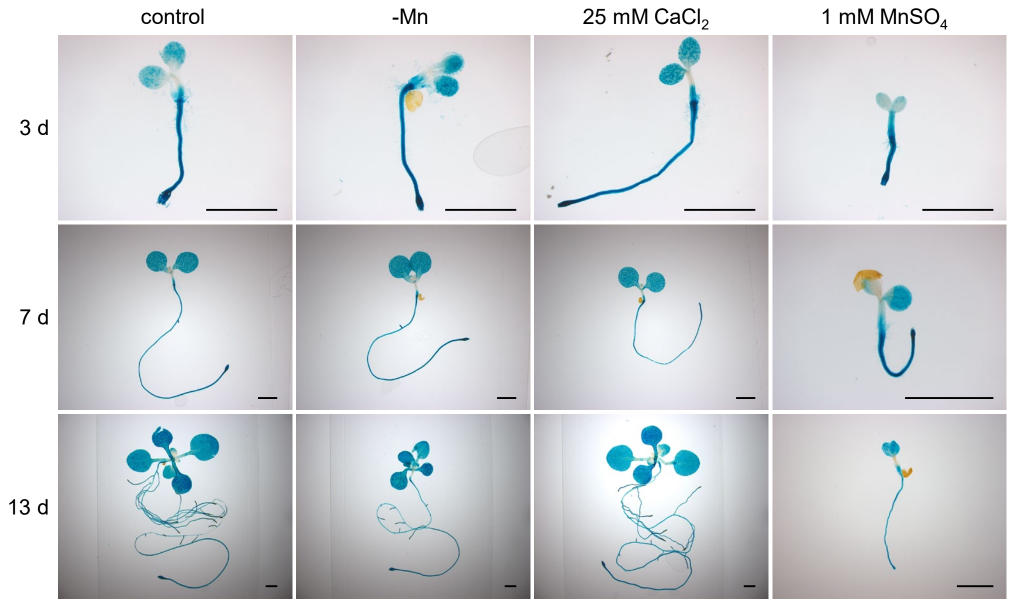

Supplemental Figure S3. GUS staining of *ProBICAT3-GUS* seedlings grown under different Mn^{2+} and Ca^{2+} supply levels.
Scale bars represent 2 mm.

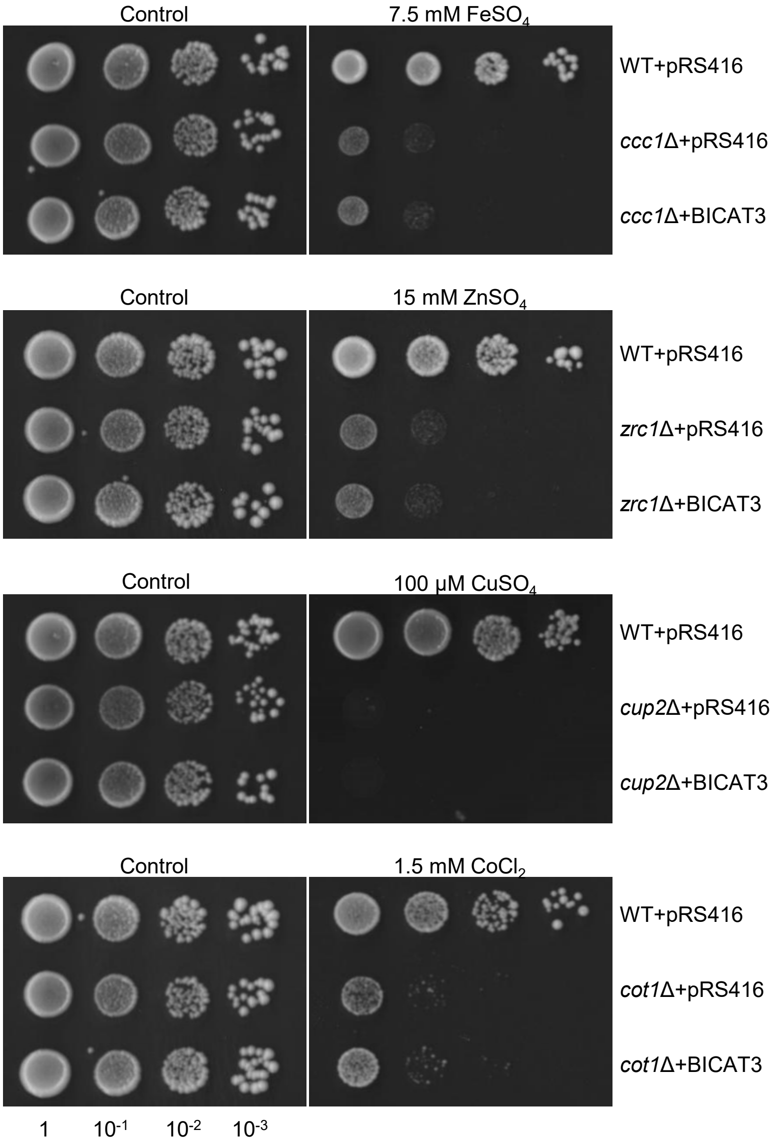

Supplemental Figure S4. BICAT3 does not complement Fe^{3+}-sensitive (*ccc1*Δ), Zn^{2+}-sensitive (*zrc1*Δ), Cu^{2+}-sensitive (*cup2*Δ), and Co^{2+}-sensitive (*cot1*Δ) yeast strains. Liquid cultures of strains were serially diluted and dropped onto media as indicated.

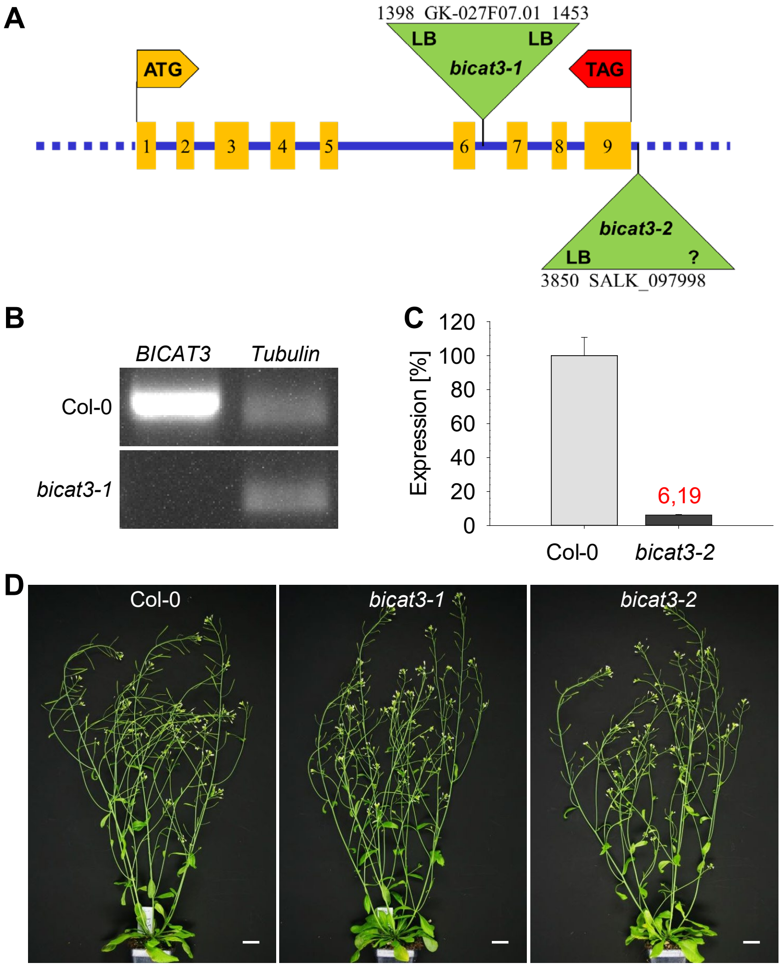

Supplemental Figure S5. Characterization of *bicat3* mutants.
(A) A model of the genomic regions and the T-DNA insertions in *BICAT3*. Coding regions are presented by boxes; introns are shown by a line; triangles indicate the sites of T-DNA insertions. The numbers indicate the first nucleotide after and the last nucleotide before the insertion, counting from the start codon. LB indicates the left border of the T-DNA, as determined by sequencing.
(B) Semi-quantitative RT-PCR of *BICAT3* in Col-0 and *bicat3-1* mutant. *Tubulin* was used as a housekeeping gene to ensure a similar amount of cDNA templates.
(C) Quantitative RT-PCR of *BICAT3* in Col-0 and *bicat3-2* mutant.
(D) Growth phenotypes of 6-week-old Col-0, *bicat3-1*, and *bicat3-2* plants grown on soil. Scale bars represent 2 cm.

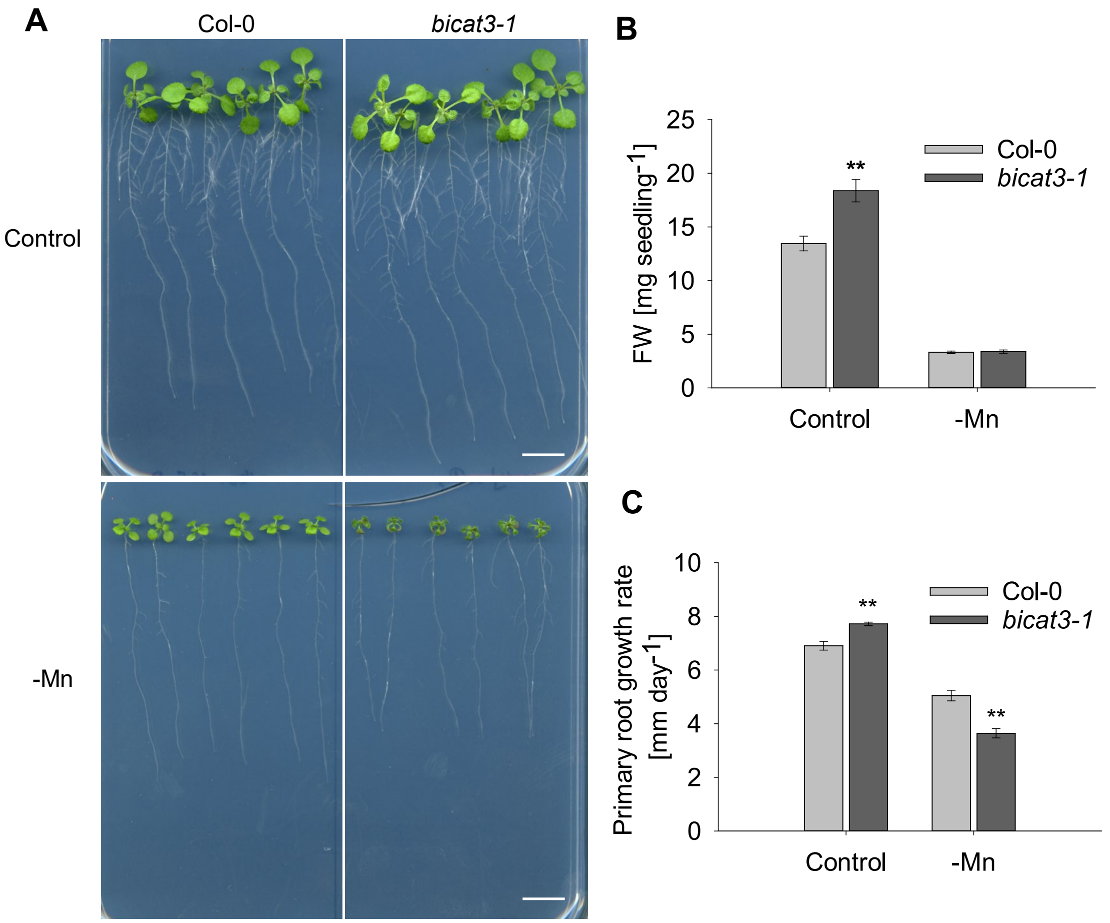

Supplemental Figure S6. *bicat3-1* shows retarded growth compared to the wild type under Mn^{2+} deficiency.
(A) Growth phenotypes of 2-week-old Col-0 and *bicat3* seedlings grown on control and Mn^{2+}-deficient plates. Scale bars represent 1 cm.
(B) Fresh weight of Col-0 and *bicat3-1* mutant grown on control and Mn^{2+}-deficient plates.
(C) Primary root growth rate of Col-0 and *bicat3-1* mutant grown on control and Mn^{2+}-deficient plates.
(B) and (C) Data represents means±SE of four plates containing six plants of each genotype. Data were analyzed by two-tailed Student's *t* test to identify significant differences between wild type and mutant (**, P< 0.01). The experiment was repeated twice with similar results.

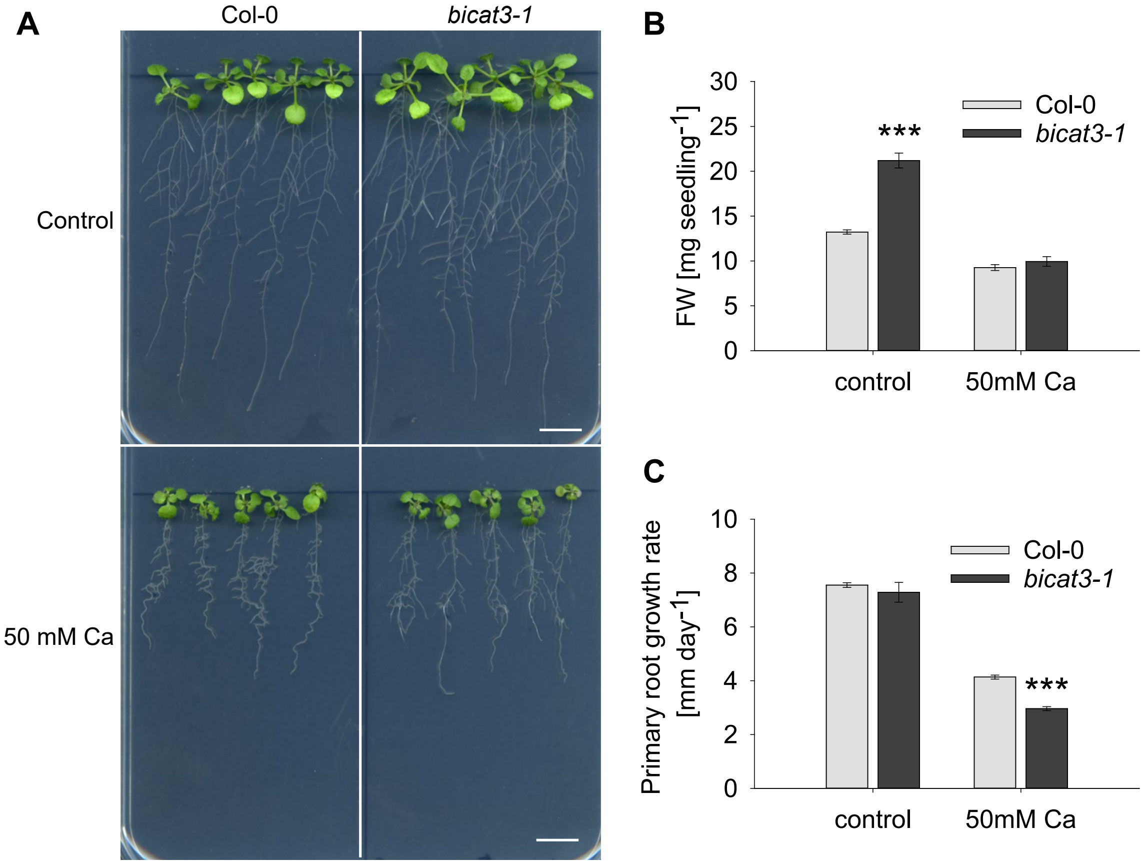

Supplemental Figure S7. *bicat3-1* shows retarded root growth compared to the wild type under Ca^{2+} toxicity.

(A) Growth phenotypes of 15-day-old Col-0 and *bicat3-1* mutants. Seedlings grew for 7 d on control plates, were subsequently transferred to control and Ca^{2+}-toxic plates, and cultured for another 8 d. Scale bars represent 1 cm.

(B) Fresh weight of Col-0 and *bicat3-1* mutant grown on control and Ca^{2+}-toxic plates.

(C) Primary root growth rate of Col-0 and *bicat3-1* mutant grown on control and Ca^{2+}-toxic plates. Data represent means±SE of three plates containing five plants of each genotype.

(B) and (C) Data represent means±SE of three plates containing five plants of each genotype. Data were analyzed by two-tailed Student's *t* test to identify significant differences between wild type and mutant (***, P<0.001). The experiment was repeated twice with similar results.

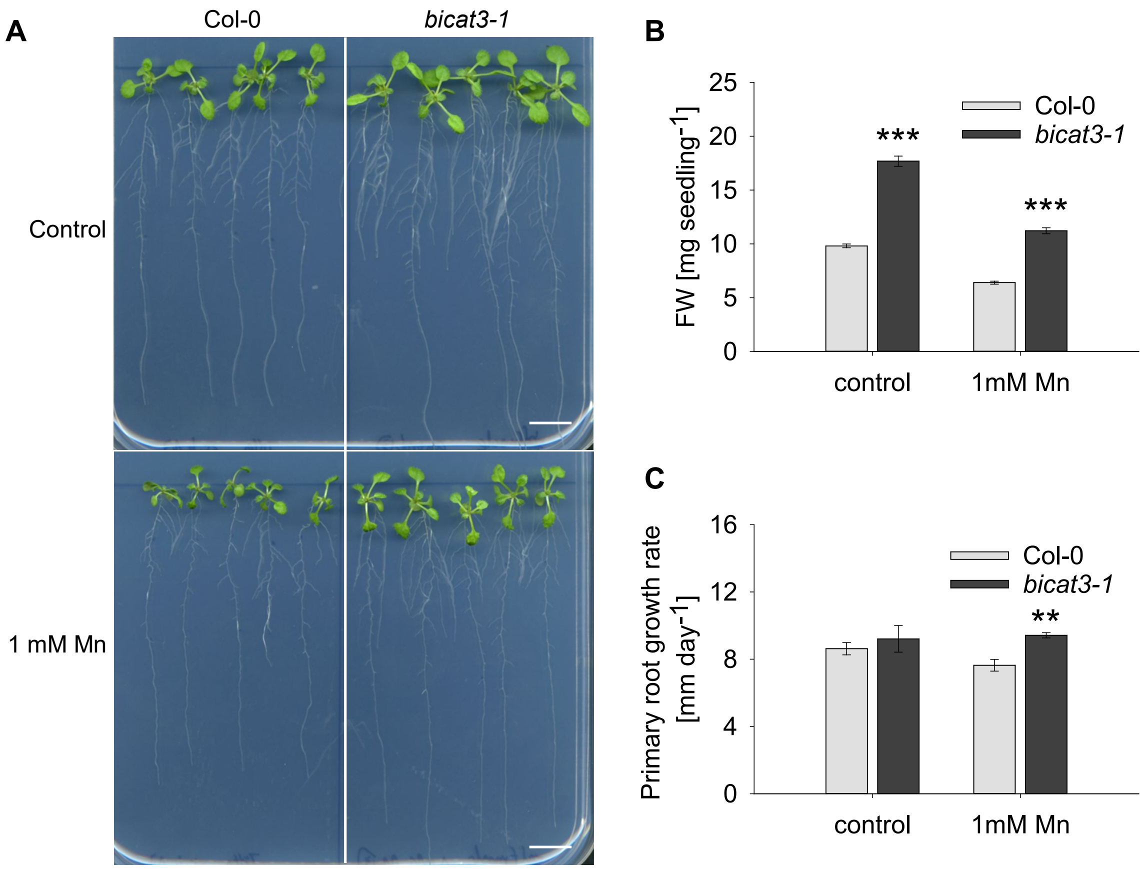

Supplemental Figure S8. *bicat3-1* shows improved growth compared to the wild type under Mn^{2+} toxicity.
(A) Growth phenotypes of 15-day-old Col-0 and *bicat3-1* mutant. Seedlings grew 7 d on control plates, were subsequently transferred to control and Mn^{2+}-toxic plates, and cultured for another 8 d. Scale bars represent 1 cm.
(B) Fresh weight of Col-0 and *bicat3-1* mutant grown on control and Mn^{2+}-toxic plates.
(C) Primary root growth rate of Col-0 and *bicat3-1* mutant grown on control and Mn^{2+}-toxic plates.
(B) and (C) Data represent means±SE of three plates containing five plants of each genotype. Data were analyzed by two-tailed Student's *t* test to identify significant differences between wild type and mutant (**, $P< 0.01$; ***, $P<0.001$). The experiment was repeated twice with similar results.

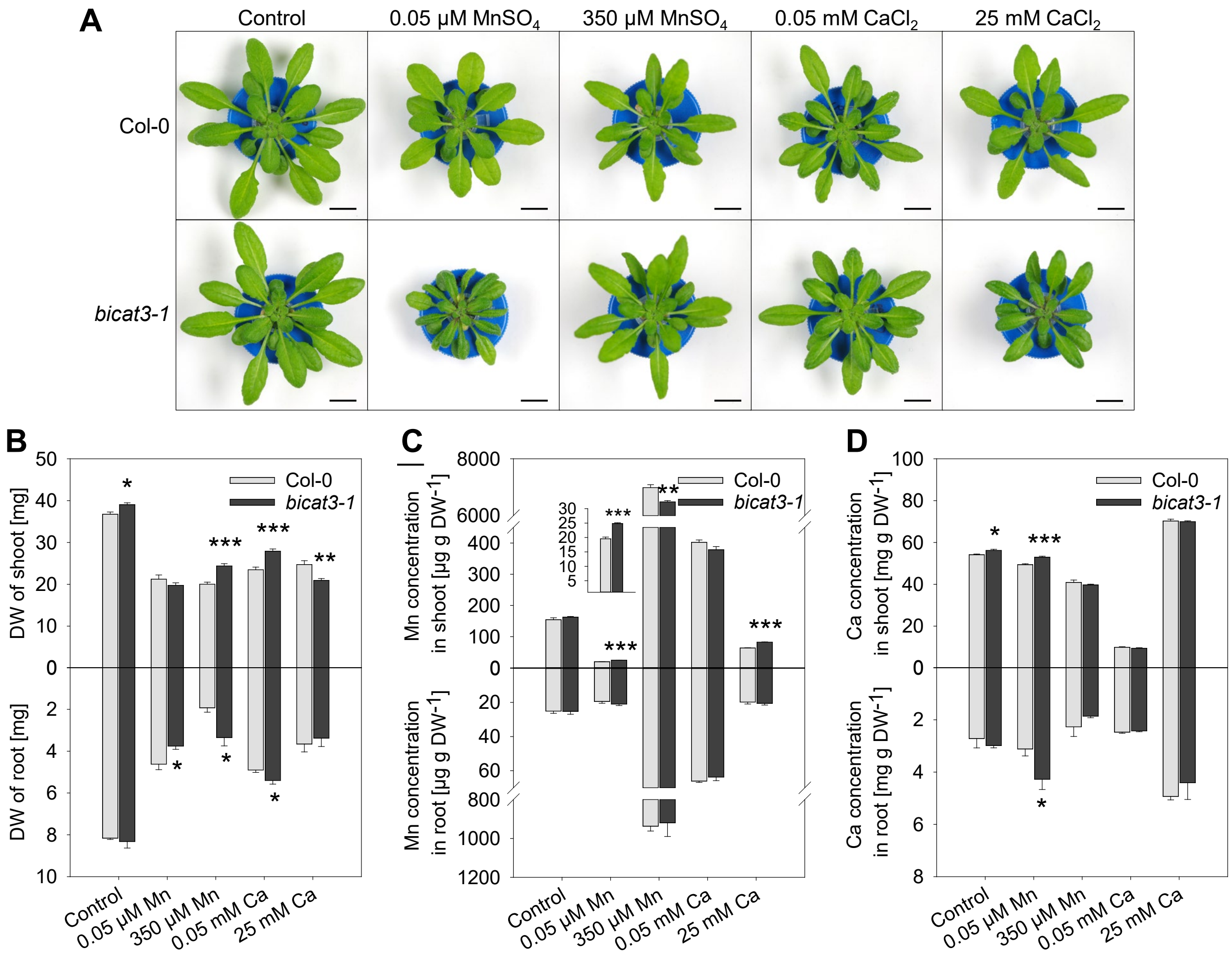

Supplemental Figure S9. Growth of Col-0 and *bicat3-1* under different Ca^{2+} and Mn^{2+} supply.

(A) Growth phenotypes of 5-week-old Col-0 and *bicat3-1* grown in hydroponics. Scale bars represents 1 cm.

(B) Shoot and root dry weight (DW) of Col-0 and *bicat3-1.*

(C) and (D) $Mn^{2+}$and Ca^{2} concentration of Col-0 and *bicat3-1* shoots and roots.

Data in (B), (C) and (D) indicate means+SE of five biological replicates. Data were analyzed by two-tailed Student's *t* test to identify significant differences between wild type and mutant (*, $P<0.05$; **, $P< 0.01$; ***, $P<0.001$). The experiment was repeated twice with similar results.

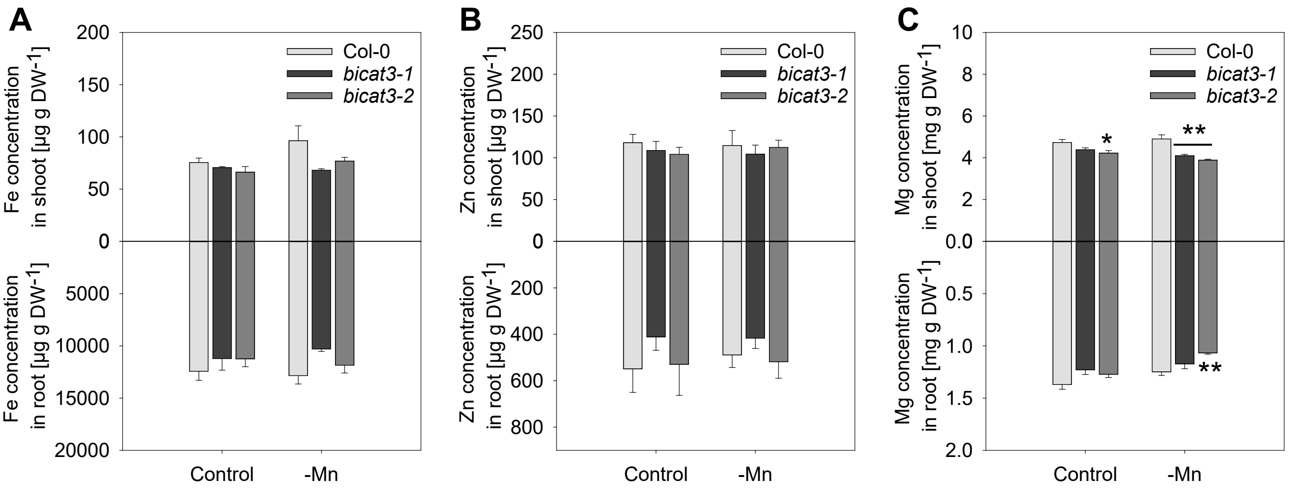

Supplemental Figure S10. Fe^{2+}, Zn^{2+}, and Mg^{2+} concentrations of 5-week-old plants cultivated in hydroponics with 3.5 µM Mn^{2+} (control) or 0.05 µM Mn^{2+} (-Mn).
(A) Fe concentration of Col-0, *bicat3-1*, and *bicat3-2* shoots and roots.
(B) Zn concentration of Col-0, *bicat3-1*, and *bicat3-2* shoots and roots.
(C) Mg concentration of Col-0, *bicat3-1*, and *bicat3-2* shoots and roots.
Data indicate means+SE of five biological replicates. Data were analyzed by two-tailed Student's *t* test to identify significant differences between wild type and mutant (*, P<0.05; **, P< 0.01).

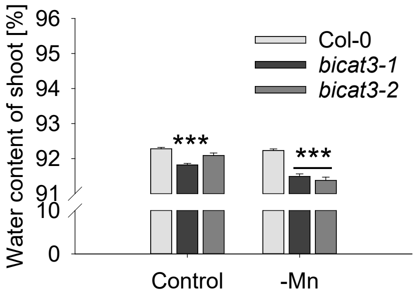

Supplemental Figure S11. Relative water content of shoots of 5-week-old Col-0, *bicat3-1*, and *bicat3-2* plants cultivated in hydroponics with 3.5 µM Mn^{2+} (control) or 0.05 µM Mn^{2+} (-Mn).
Data indicate means+SE of five biological replicates. Data were analyzed by two-tailed Student's *t* test to identify significant differences between wild type and mutant (***, $P<0.001$). The experiment was repeated twice with similar results.

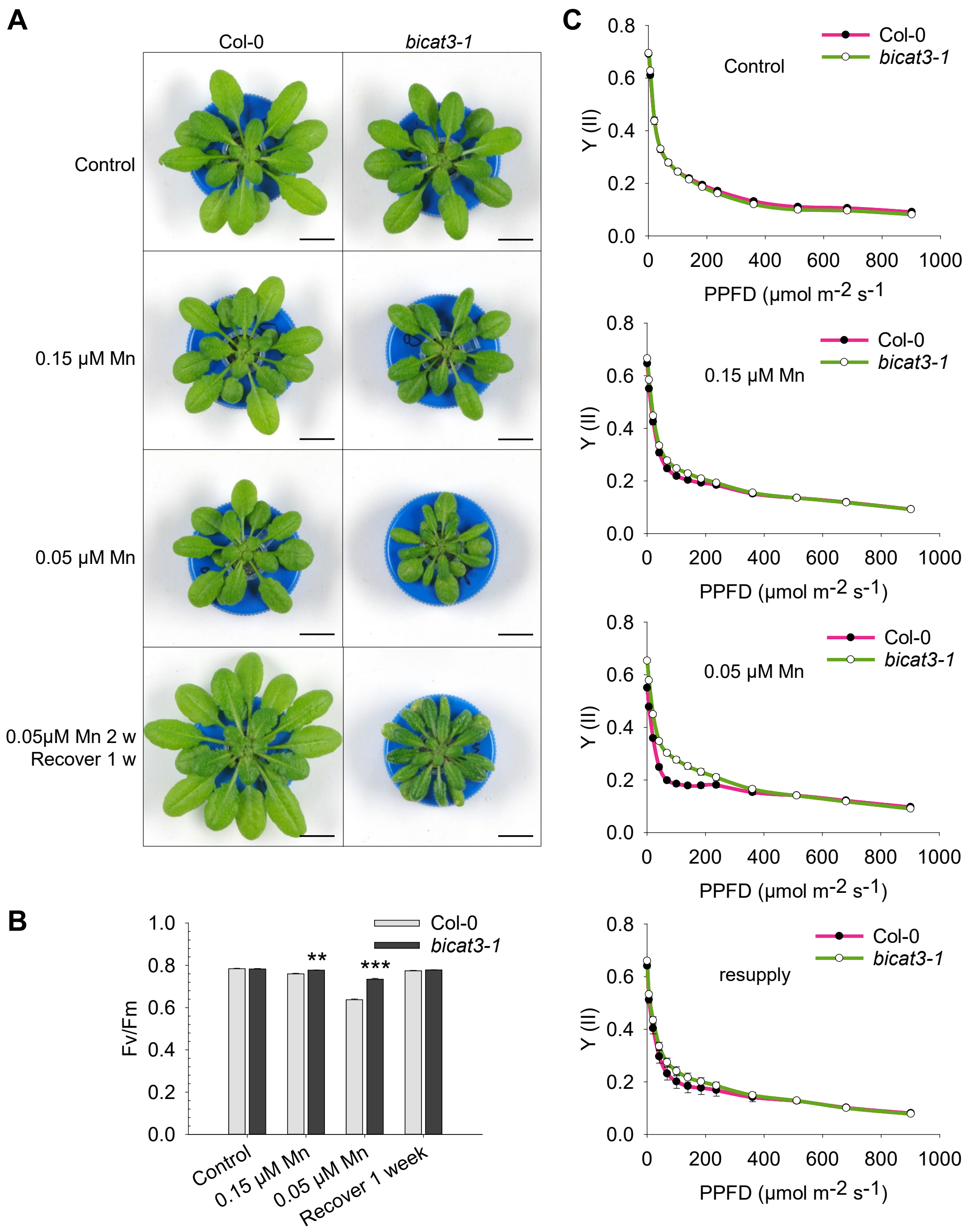

Supplemental Figure S12. Phenotypes of Col-0 and *bicat3-1* under different Mn^{2+} supply levels.

(A) Growth of 5-week-old Col-0 and *bicat3-1* in hydroponics under different Mn supply and after Mn^{2+} resupply. Scale bars represent 1 cm.

(B) Fv/Fm of Col-0 and *bicat3-1* under different Mn^{2+} supply and after Mn resupply.

(C) Efficiency of photosystem II [Y (II)] of Col-0 and *bicat3-1* under different Mn^{2+} supply and after Mn^{2+} resupply.

(B) and (C) Data indicate means±SE of five biological replicates. Data were analyzed by two-tailed Student's *t* test to identify significant differences between wild type and mutant (**, P< 0.01; ***, P<0.001). The experiment was repeated twice with similar results.

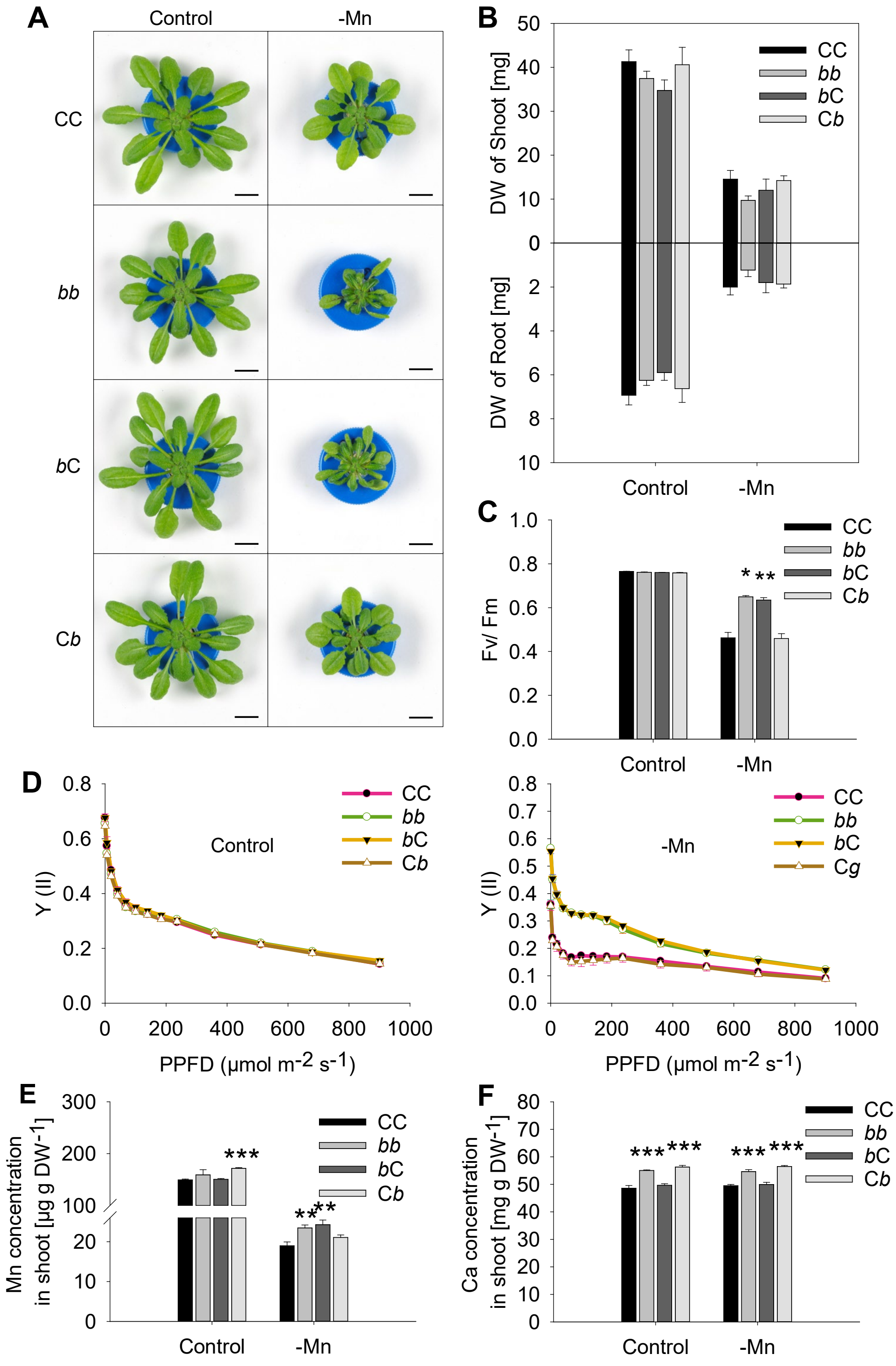

Supplemental Figure S13. Phenotypes of reciprocally grafted Col-0 and *bicat3-1* plants under control and Mn^{2+} deficiency (0.05 µM Mn^{2+}) conditions.

(A) Growth phenotypes of grafted plants. *CC*, Col-0 shoot and Col-0 root; *bb, bicat3-1* shoot and *bicat3-1* root; *b*C, Col-0 shoot and *bicat3-1* root; C*b*, Col-0 shoot and *bicat3-1* root. Scale bars represent 1 cm.

(B) Dry weight of shoots and roots of grafted plants.

(C) Fv/Fm of grafted plants.

(D) Efficiency of photosystem II [Y (II)] of grafted plants.

(E) Mn^{2+} concentrations in shoots of grafted plants.

(F) Ca^{2+} concentrations in shoots of grafted plants.

Data indicate means+SE of at least three biological replicates. Data were analyzed by two-tailed Student's *t* test to identify significant differences between *bb*, *b*C, C*b* and *CC* (*, P<0.05**, P< 0.01; ***, P<0.001). The experiment was repeated twice with similar results.

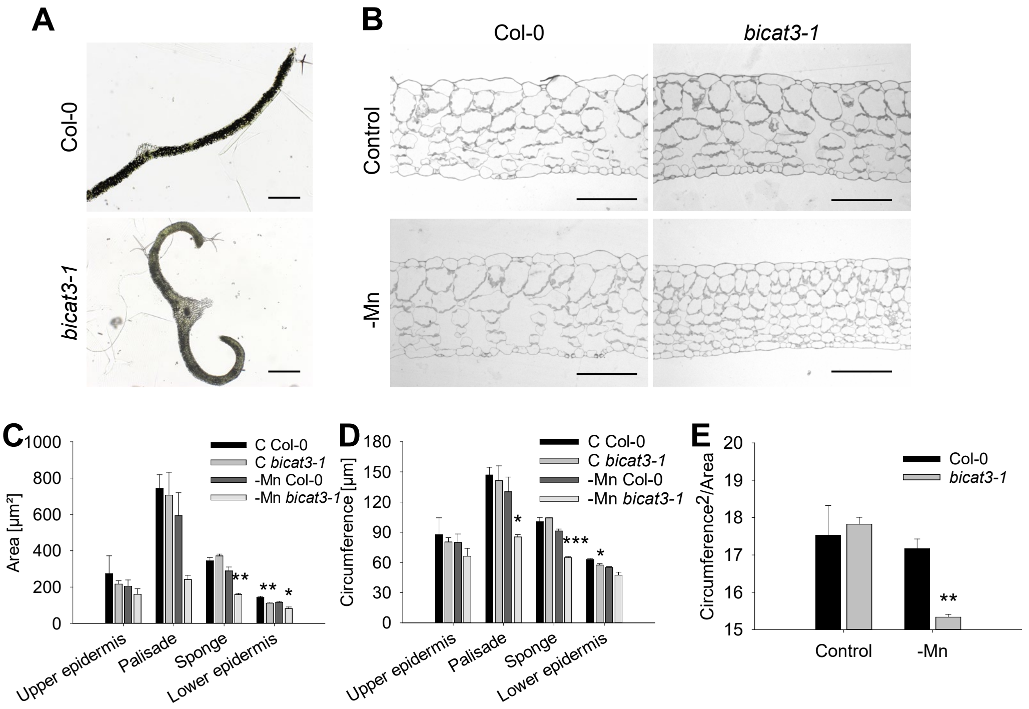

Supplemental Figure S14. The cell size and shape of *bicat3-1* leaves changes under Mn^{2+} deficiency (0.05 µM Mn^{2+}) compared to the wild type.

(A) Cross section of 5-week-old Col-0 and *bicat3-1* leaf grown in hydroponics under Mn^{2+} deficiency. Scale bars represent 500 µm.

(B) Cross sections of Col-0 and *bicat3-1* leaf blade under control and Mn^{2+} deficiency conditions. Scale bars represent 100 µm.

(C) Sectional area of different cell types of Col-0 and *bicat3-1* leaf blade under control and Mn^{2+} deficiency conditions.

(D) Circumference of different cell types of Col-0 and *bicat3-1* leaf blade under control and Mn^{2+} deficiency conditions.

(E) Circularity (circumference^2/area) of spongy mesophyll cells under control and Mn^{2+} deficiency conditions.

(C) to (E). Depending on the amount of cells, 10-100 cells were quantified in each section. The means of all cells from two sections of the same leaf were calculated and averaged, representing one biological replicate. Data represent the means+SE of three biological replicates. Data were analyzed by two-tailed Student's *t* test to identify significant differences between wild type and mutant (*, $P<0.05$; **, $P< 0.01$; ***, $P<0.001$).

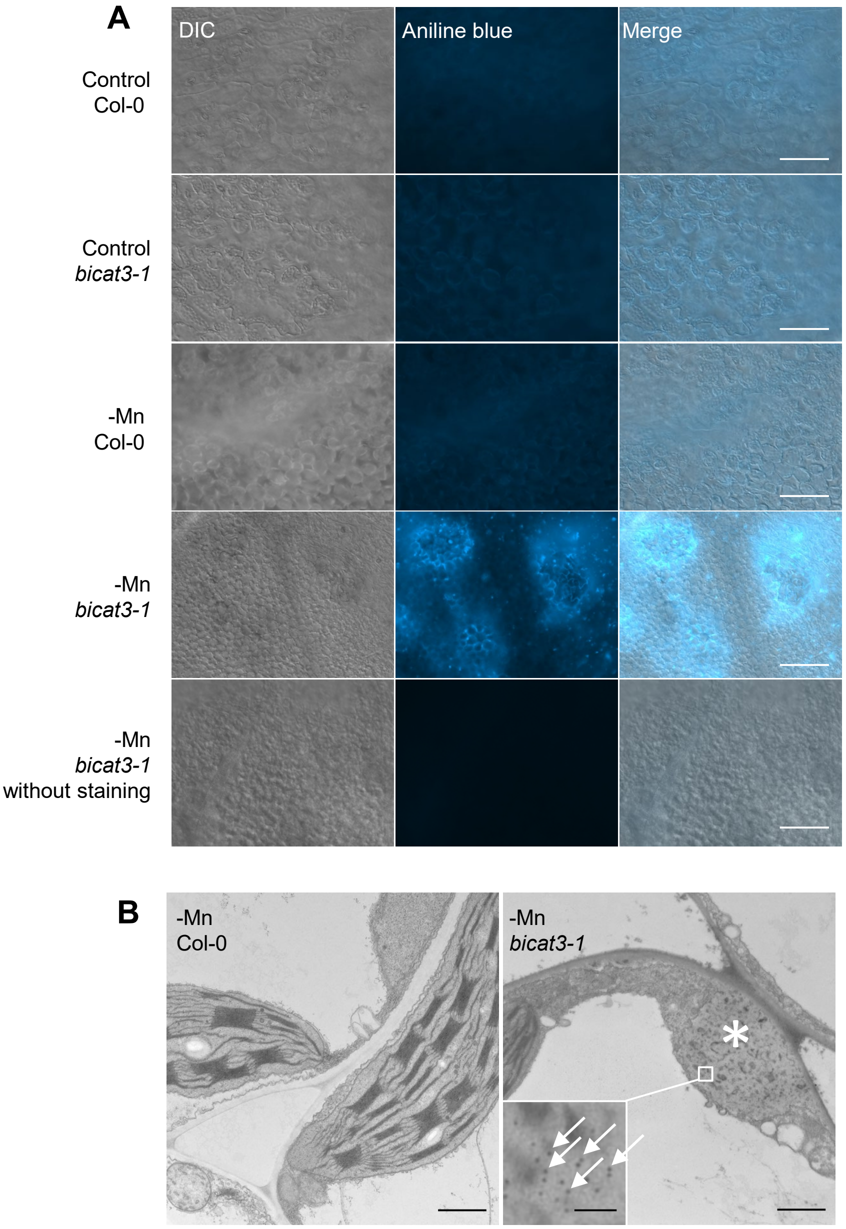

Supplemental Figure S15. *bicat3-1* accumulates more callose in leaves compared to the wild type under Mn^{2+} deficiency (0.05 µM Mn^{2+}).
(A) Callose accumulation in 5-week-old hydroponically cultured Col-0 and *bicat3-1* leaf detected by aniline blue staining. Scale bars represent 100 µm.
(B) Callose detection in Mn^{2+}-deficient Col-0 and *bicat3-1* by immunogold staining. Opposite to Col-0, mesophyll cells of *bicat3-1* contain regular plugs (asterisk) formed by callose. This is shown by numerous gold particles (Insert, arrows). Scale bars represent 1 µm; scale bar in inset represents 0.1 µm.

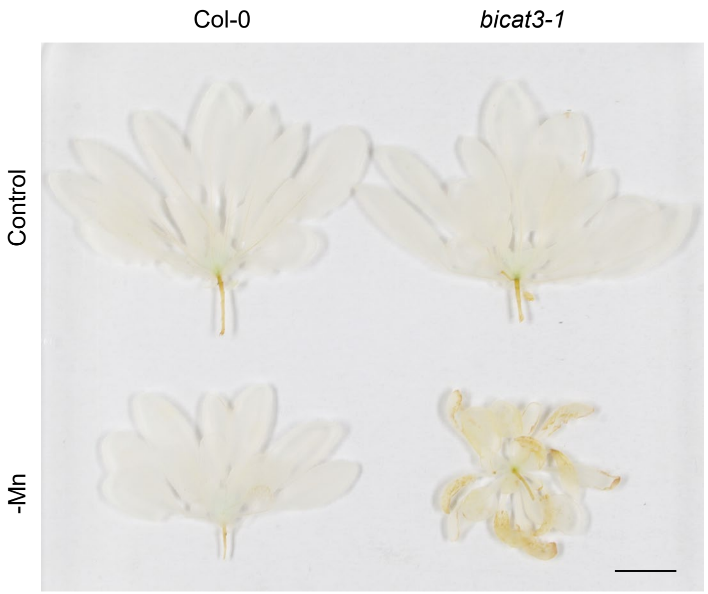

Supplemental Figure S16. *bicat3-1* accumulates more H_2O_2 in leaves compared to the wild type under Mn^{2+} deficiency (0.05 µM Mn^{2+}).
Plants plants were grown for 5 weeks in hydroponics. H_2O_2 accumulation was detected by DAB (3,3'-diaminobenzidine) staining. Scale bar represents 1 cm.

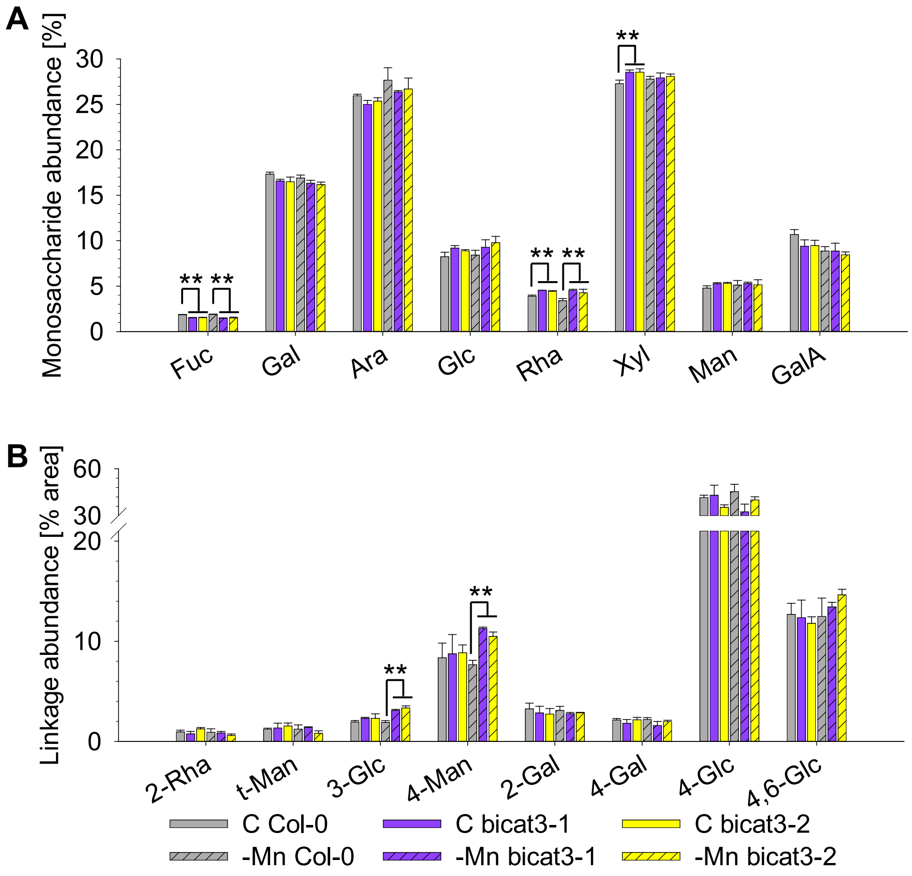

Supplemental Figure S17. Cell wall matrix sugar components in *bicat3* roots compared to the wild type under Mn^{2+} deficiency (0.05 µM Mn^{2+}) and control conditions.

(A) Monosaccharide composition of root cell wall alcohol-insoluble residue. Col-0 and *bicat3* plants were cultured for 5 weeks in hydroponics with 3.5 µM Mn^{2+} (C) or 0.05 µM Mn (-Mn).

(B) Glycosidic linkages of Col-0 and *bicat3* root cell wall alcohol-insoluble residue. Values represent molar percentage of total carbohydrates detected.

Data in (A) and (B) indicate means+SD of four and three independent biological replicates, respectively. The complete data set can be found in Supplementary Table 1. Data were analyzed by two-tailed Student's *t* test to identify significant differences between wild type and mutants (**, P< 0.01).

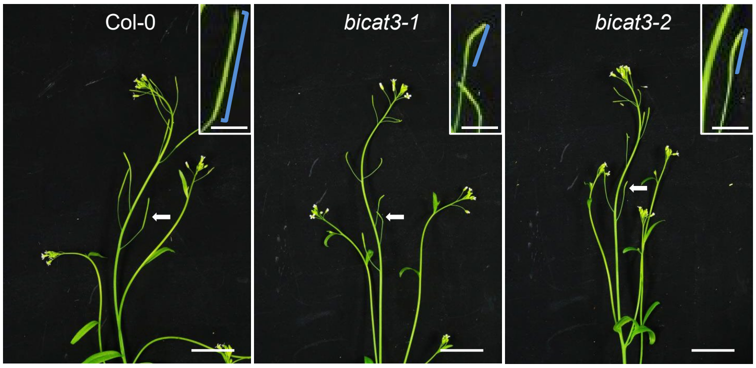

Supplemental Figure S18. *bicat3-1* and *bicat3-2* mutants produce shorter siliques compared to the wild type.
Plants were grown under greenhouse conditions. Scale bars represent 2 cm; scale bars in insets represent 0.5 cm.

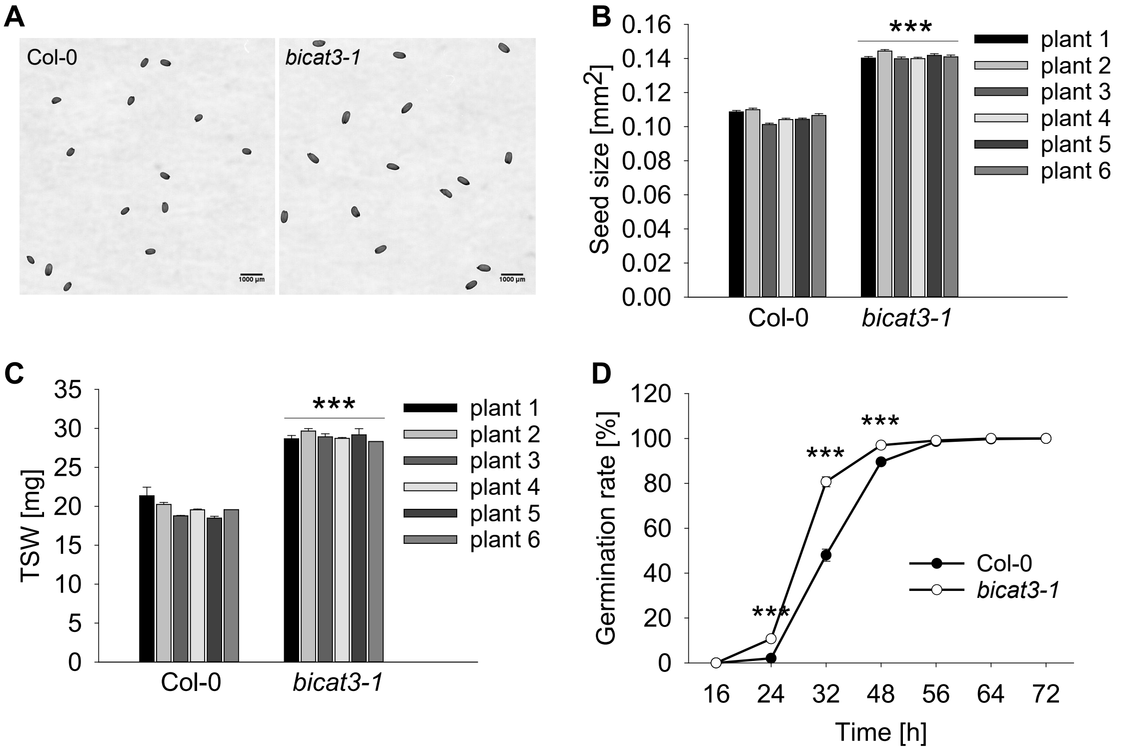

Supplemental Figure S19. The *bicat3-1* mutant produces larger seeds and germinates faster than the wild type.

(A) Col-0 seeds (left) and *bicat3-1* seeds (right). Scale bars represent 1 mm.

(B) Seed size of *bicat3-1* and Col-0. Data indicate means+SE of at least 200 seeds per plant.

(C) Thousand seed weight of *bicat3-1* and Col-0. Data indicate means+SE of three technical replicates.

(D) Germination of *bicat3-1* and Col-0 seeds. Data indicate means±SE of seven replicates with at least sixty seeds.

(B)-(D) Data were analyzed by two-tailed Student's *t* test to identify significant differences between wild type and mutant (***, $P < 0.001$). The experiment was repeated twice with similar results.

Col-0

bicat3-1

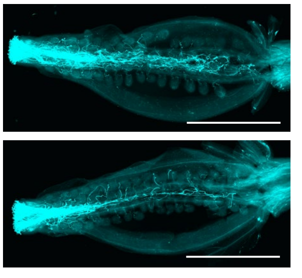

Supplemental Figure S20. Aniline blue staining of Col-0 and *bicat3-1* pollen tubes grown for 48 hours *in vivo*.
Scale bars represent 1 mm.

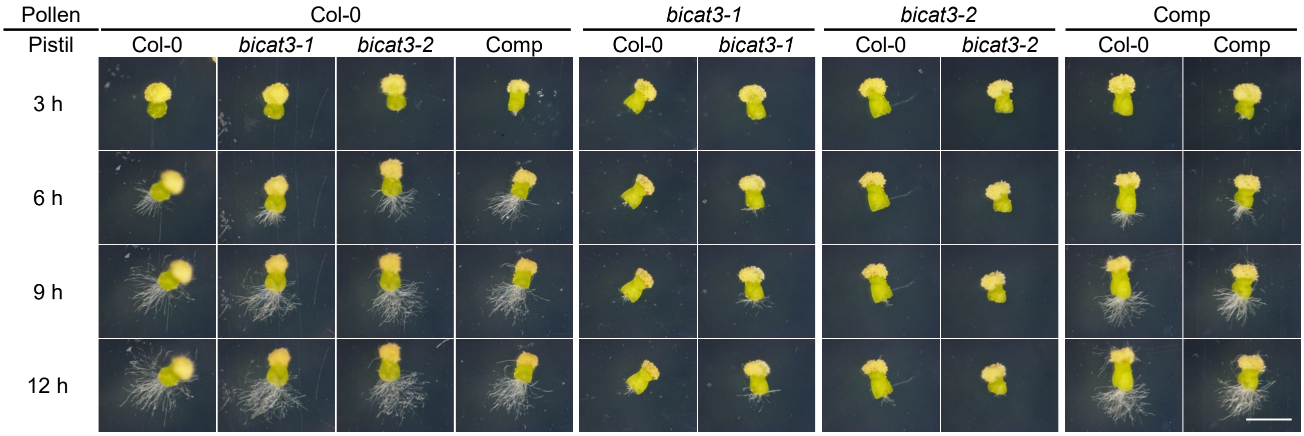

Supplemental Figure S21. *Semi in vivo* pollen tube growth assay of Col-0, *bicat3-1*, *bicat3-2*, and *bica3-1* complemented by expression of *BICAT3* driven by its native promoter.
Pictures were taken 3, 6, 9, and 12 h after pollination. Scale bar represents 1 mm and applies to all images.

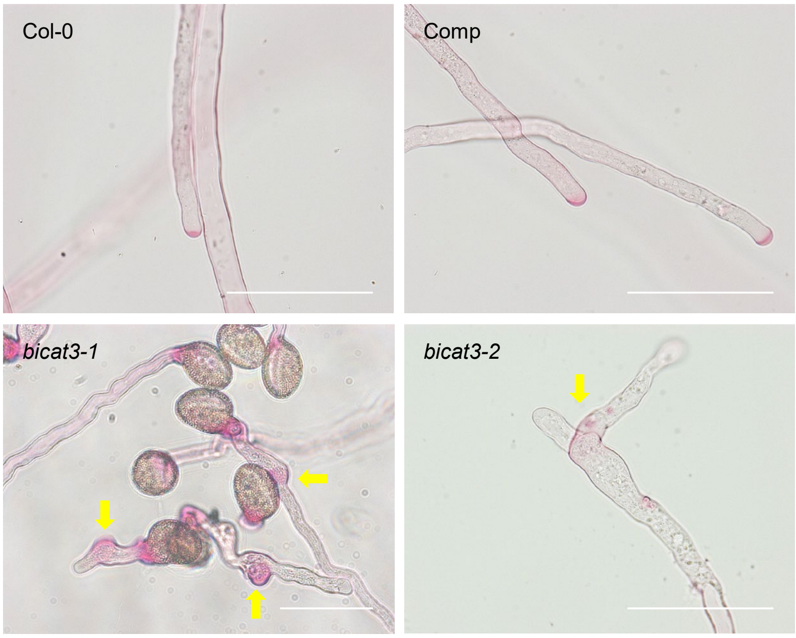

Supplemental Figure S22. *bicat3* mutant pollen tubes grow aberrantly *in vitro* and show abnormal pectin distribution.

Pollen were cultured 6 h *in vitro.* Pectin was detected by ruthenium red staining. Arrows indicate abnormal pectin deposition. Scale bars represent 50 µm.

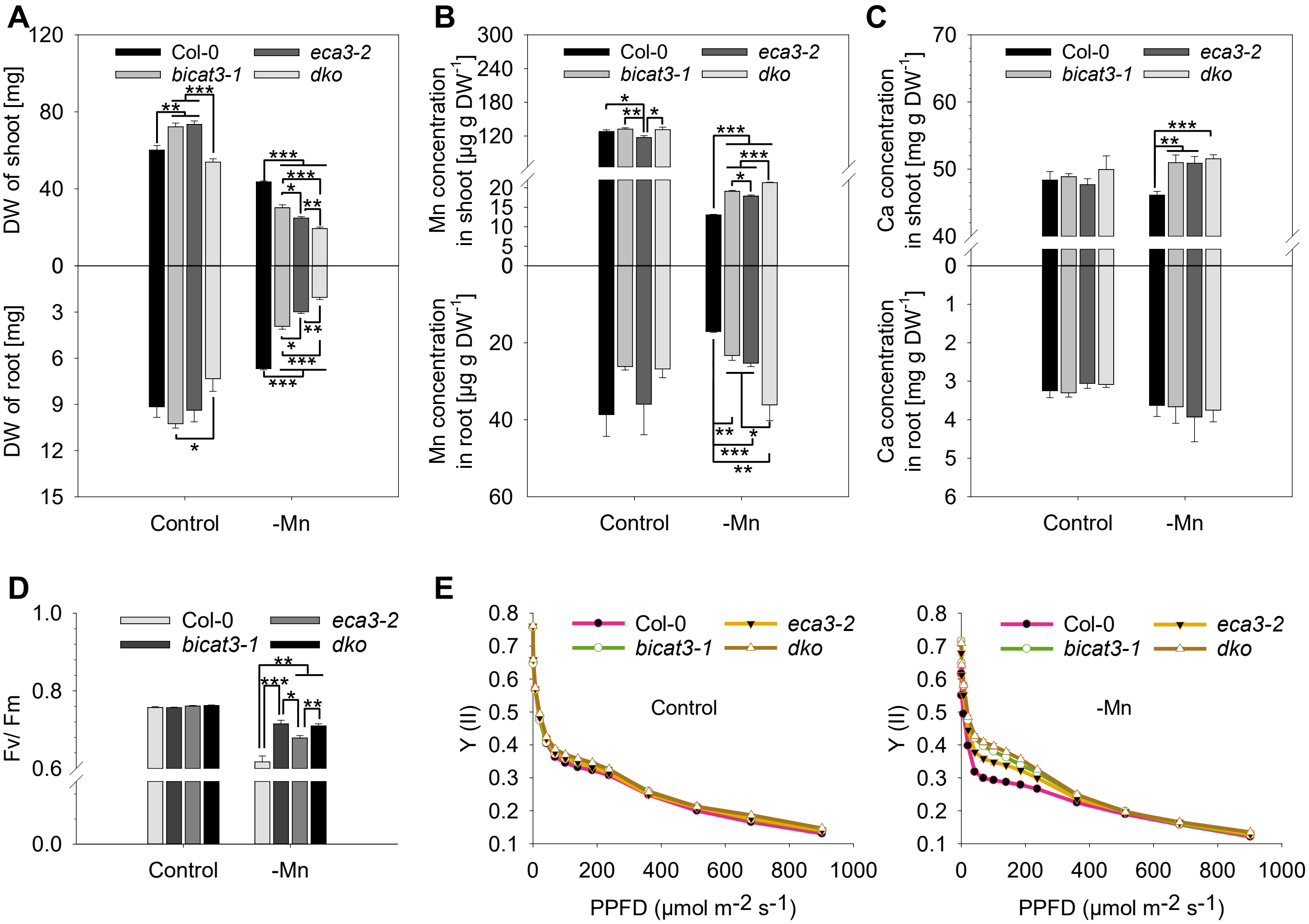

Supplemental Figure S23. BICAT3 and ECA3 distinctly determine growth, Mn^{2+} and Ca^{2+} accumulation, and photosynthesis under Mn^{2+} limitation.

(A) Dry weight (DW) of 6-week-old Col-0, *bicat3-1*, *eca3-2*, and *dko* (*bicat3-1 eca3-2*) plants grown in hydroponics under control (3.5 µM) and Mn^{2+}-deficient (0.05 µM) conditions.

(B) Mn^{2+} concentration of Col-0, *bicat3-1*, *eca3-2*, and *dko* plants under control and Mn^{2+}-deficient conditions.

(C) Ca^{2+} concentration of Col-0, *bicat3-1*, *eca3-2*, and *dko* plants under control and Mn^{2+}-deficient conditions.

(D) Fv/Fm of Col-0, *bicat3-1*, *eca3-2*, and *dko* plants under control and Mn^{2+}-deficient conditions.

(E) Efficiency of photosystem II [Y (II)] of Col-0, *bicat3-1*, *eca3-2*, and *dko* plants under control and Mn^{2+}-deficient conditions.

Data indicate means+SE of four biological replicates. Data were analyzed by two-tailed Student's *t* test to identify significant differences (*, $P<0.05$; **, $P< 0.01$; ***, $P<0.001$). The experiment was repeated twice with similar results.

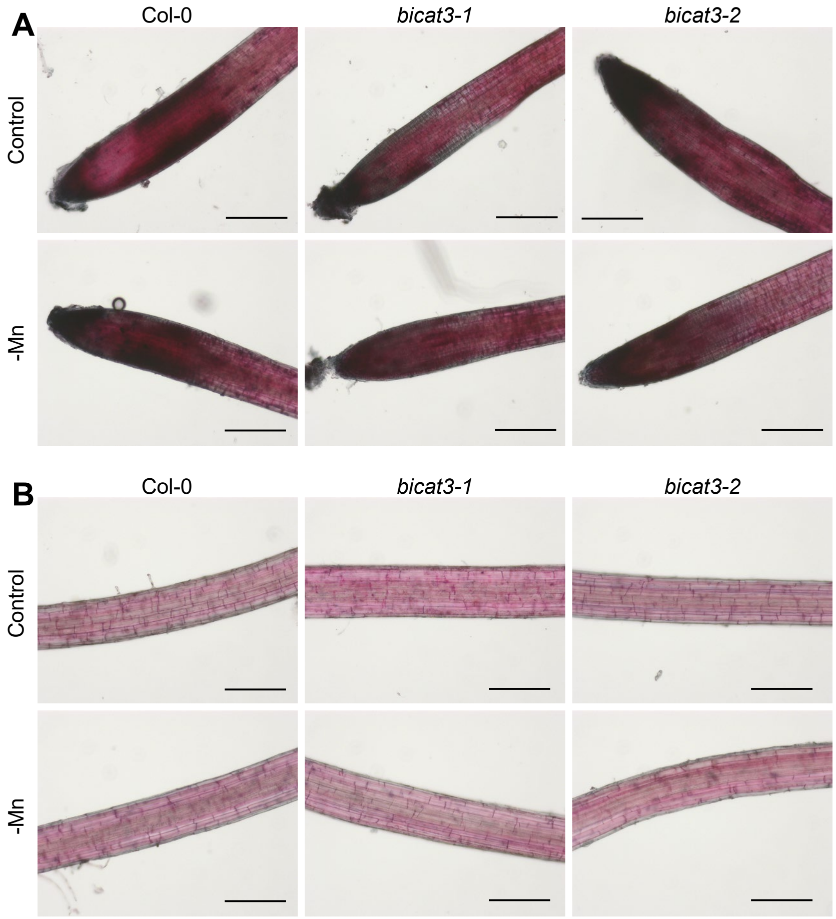

Supplemental Figure S24. Roots of *bicat3* mutants show no morphological defects under Mn^{2+} deficiency.
(A) Root tips of 5-week-old Col-0 and *bicat3* mutants grown in hydroponics. Bar represents 200 µm.
(B) Mature roots. Bar represents 200 µm.
Roots were stained with Ruthenium Red, indicating similar amounts of pectin. The experiment was repeated twice with similar results.

Supplemental Table S1. Glycosidic linkages of Col-0 and *bicat3* shoot and root cell wall alcohol-insoluble residue. Data indicate means and SD of three independent biological replicates. Data were analyzed by two-tailed Student's *t* test to identify significant differences (*, P<0.05; **, P< 0.01).

Linkage abundance (% area)		Control						$-Mn^{2+}$					
		Col-0		*bicat3-1*		*bicat3-2*		Col-0		*bicat3-1*		*bicat3-2*	
		Average	sd	Average	sd	Average	sd	Average	sd	Average	sd	Average	sd
Shoot	2-Rha	1.07%	0.22%	1.04%	0.19%	0.95%	0.05%	1.15%	0.10%	2.86% *	0.74%	1.77% *	0.26%
	t-Man	0.47%	0.07%	0.46%	0.06%	0.42%	0.13%	0.35%	0.04%	0.86% **	0.11%	0.83% *	0.25%
	t-Glc	0.97%	0.56%	0.77%	0.36%	0.49%	0.28%	0.65%	0.37%	0.48%	0.12%	0.23%	0.01%
	t-Gal	3.58%	0.52%	3.24%	0.51%	3.12%	0.63%	3.22%	0.50%	2.89%	0.27%	2.89%	0.70%
	5-Ara	2.22%	0.37%	2.18%	0.25%	2.10%	0.42%	1.53%	0.34%	1.53%	0.30%	1.31%	0.19%
	2-Xyl/4-Xyl	3.60%	0.54%	3.08%	0.44%	2.97%	0.67%	2.48%	1.20%	4.42%	1.02%	2.69%	0.82%
	3-Glc	2.73%	0.15%	3.17%	0.53%	3.31% *	0.20%	2.92%	0.67%	12.11%**	1.51%	11.62%**	3.13%
	4-Man	8.23%	0.65%	8.36%	0.56%	8.23%	0.32%	7.18%	1.16%	11.65% *	1.43%	12.60% *	1.91%
	2-Gal	2.07%	0.41%	1.70%	0.07%	1.83%	0.12%	2.08%	0.42%	0.94% *	0.16%	0.88% **	0.02%
	4-Gal	6.57%	0.39%	6.98%	0.50%	7.50% *	0.25%	3.85%	1.09%	1.09% *	0.04%	1.12% *	0.15%
	4-Glc	43.11%	1.91%	46.30%	0.85%	43.90%	1.18%	49.91%	3.44%	38.74% *	4.74%	41.00% *	4.12%
	2,4-Xyl	1.06%	0.24%	1.05%	0.17%	1.25%	0.32%	1.06%	0.32%	0.99%	0.15%	1.06%	0.11%
	6-Gal	1.04%	0.27%	1.20%	0.11%	1.22%	0.17%	1.39%	0.28%	1.60%	0.18%	1.82%	0.40%
	3,4-Glc	1.68%	0.79%	1.32%	0.21%	1.19%	0.15%	1.22%	0.12%	1.41%	0.28%	1.31%	0.35%
	4,6-Man	1.21%	0.08%	1.16%	0.03%	1.37%	0.36%	1.16%	0.54%	2.10%	0.62%	2.43%	0.69%
	4,6-Glc	17.42%	1.74%	16.00%	0.16%	17.73%	1.37%	18.08%	2.35%	13.98%	1.56%	14.26%	0.74%
	4,6-Gal	0.82%	0.15%	0.91%	0.27%	1.07% *	0.04%	0.34%	0.06%	0.31%	0.27%	0.42%	0.45%
	3,6-Gal	2.15%	0.69%	1.07%	0.40%	1.35%	0.24%	1.44%	0.43%	2.04%	0.38%	1.76%	0.05%
root	2-Rha	0.95%	0.18%	0.75%	0.25%	1.26%	0.13%	0.91%	0.35%	0.84%	0.15%	0.60%	0.15%
	t-Man	1.23%	0.10%	1.34%	0.48%	1.54%	0.30%	1.23%	0.41%	1.40%	0.07%	0.81%	0.25%
	t-Glc	0.42%	0.17%	0.36%	0.07%	0.75%	0.15%	0.60%	0.55%	0.99%	0.45%	0.44%	0.26%
	t-Gal	1.94%	0.29%	2.07%	0.42%	2.69% *	0.33%	2.12%	0.85%	1.95%	1.37%	1.96%	0.69%
	5-Ara	1.12%	0.18%	0.96%	0.25%	1.44%	0.09%	0.98%	0.40%	1.22%	0.08%	0.82%	0.19%
	2-Xyl/4-Xyl	7.62%	3.51%	8.07%	3.40%	13.58%	2.67%	7.55%	4.77%	9.86%	2.29%	4.49%	1.60%
	3-Glc	1.94%	0.15%	2.31% *	0.10%	2.31%	0.45%	1.90%	0.18%	3.09% **	0.11%	3.34% **	0.20%
	4-Man	8.35%	1.46%	8.74%	1.92%	8.85%	0.78%	7.66%	0.44%	11.27%**	0.13%	10.48%**	0.44%
	2-Gal	3.25%	0.57%	2.85%	0.67%	2.73%	0.56%	3.09%	0.42%	2.77%	0.13%	2.86%	0.05%
	4-Gal	2.14%	0.14%	1.81%	0.37%	2.16%	0.25%	2.18%	0.19%	1.58%	0.41%	1.98%	0.14%
	4-Glc	41.59%	1.55%	43.07%	6.44%	35.35%**	1.59%	45.39%	4.71%	32.42% *	5.01%	40.07%	1.96%
	2,4-Xyl	1.68%	0.17%	1.57%	0.19%	1.83%	0.23%	1.53%	0.16%	2.03% *	0.23%	1.88% *	0.07%
	6-Gal	2.15%	0.29%	2.75%	0.88%	2.40%	0.29%	1.74%	0.30%	3.31% **	0.46%	2.87% **	0.30%
	3,4-Glc	1.23%	0.18%	1.09%	0.21%	1.22%	0.34%	1.47%	0.33%	1.40%	0.37%	1.72%	0.27%
	4,6-Man	6.42%	1.34%	5.71%	1.46%	5.76%	1.04%	5.23%	0.69%	7.20%	1.12%	7.18%	0.44%
	4,6-Glc	12.68%	1.11%	12.37%	1.75%	11.79%	0.66%	12.47%	1.84%	13.43%	0.46%	14.63%	0.56%
	4,6-Gal	0.22%	0.02%	0.26%	0.08%	0.20%	0.09%	0.23%	0.02%	0.43%	0.14%	0.39%	0.23%
	3,6-Gal	5.07%	0.69%	3.89%	1.04%	4.15%	0.55%	3.71%	0.43%	4.80%	0.73%	3.48%	0.29%

Supplemental Table S2. Primers and constructs used in this study.

Number	Name	Sequence 5'- 3'	Purpose
PEO1453	T-DNA *bicat3-1*_F	TTGATATCGAGGAAGCACACC	Screening
PEO1454	T-DNA *bicat3-1*_R	CTCCTATGGCGTTCTTGTGAG	Screening
PEO1645	T-DNA *bicat3-2*_F	TGAAGTTGGCGACTCACAAG	Screening
PEO1646	T-DNA *bicat3-2*_R	CATCATCACGGGTATCCACA	Screening
PEO54	GABI_pAC161-8409	ATATTGACCATCATACTCATTGC	Screening
PEO17	SALK_LB	TGGTTCACGTAGTGGGCCATCG	Screening
PEO474	*TUB6*_F	AATCGACACTTTCAGTTCATCAGC	Screening
PEO475	*TUB6*_R	ATCCTCGTCTTCTTCATACTCGCC	Screening
PEO1423	*BICAT3*_F1	acccgggatccATGGGTTTGATTTCAAACCCTAC	Screening/ Cloning
PEO1424	*BICAT3*_R1	aaagtcgacCTACAATGGAGGATAGAAATAGGAGG	Screening/ Cloning
PEO1487	qRT *BICAT3*_F	TCGCCACCATCTTCTTCCTC	Screening (qRT-PCR)
PEO1488	qRT *BICAT3*_R	AACCATTCCACGACGACCAA	Screening (qRT-PCR)
PEO045	*Actin2_F*	TCCCTCAGCACATTCCAGCAGAT	Screening (qRT-PCR)
PEO046	*Actin2_R*	AACGATTCCTGGACCTGCCTCATC	Screening (qRT-PCR)
PEO1613	Loc *BICAT3*_F2	aacccgggATGGGTTTGATTTCAAACCCTAC	colocalization
PEO1425	Loc *BICAT3*_R2	acccgggCAATGGAGGATAGAAATAGGAGGA	colocalization
PEO1642	Pro *BICAT3*_F	aacccgggGCCCTGTTCGTTTGCCTGT	Promoter
PEO1474	Pro *BICAT3*_R	ttcccgggGAGTGATCTGAGAGTTTTGCAGAT	Promoter
PEO1473	Comp *BICAT3*_F	aacccgggATAACAAATGTTTTACTTTGCTGTG	Complementation
PEO1610	Comp *BICAT3*_R	aacccgggCACGACATCTCTCATCTCTC	Complementation
PEO2330	User Pro *BICAT3*_F	GGCTTAAUGCCCTGTTCGTTTGCCTGTCGC	Complementation
PEO2331	User Pro *BICAT3*_R	AGCCTCCTUAGCAGCTGCCTCTGCGAGTGATCTGAGAGTTTTGCAGAT	Complementation
PEO2332	User *BICAT3-Venus*_F	AAGGAGGCUGCAGCTAAGGCTATGGGTTTGATTTCAAACCCTAC	Complementation
PEO2333	User *BICAT3-Venus*_R	ATTTAGGUTCAGGAGCTGGTTTTTATCCCTTGTACAGCT	Complementation
PEO2149	User Nos_F	ACCTAAAUGCACCTCCAATTCCCGATCGTTCAAAC	Complementation
PEO2150	User Nos_R	GGTTTAAUCCCCGATCTAGTAACATAGAT	Complementation

Constructs	Vectors	Inserts	Primers	Restriction sites
pGreenII0229-gBICAT3	pGreenII0229	genomic DNA of *BICAT3*	PEO1424+ PEO1624	*XmaI*
pCAMBIA2300u-PrBICAT3-BICAT3-Venus-tNos	pCAMBIA2300u	*BICAT3* promoter	PEO2330+ PEO2331	PacI /Nt.BbvCI<br>User cassette
		BICAT3-Venus	PEO2332+ PEO2333	User cassettes
		NOS terminator	PEO2149+ PEO2150	User cassette<br>PacI /Nt.BbvCI
pART7-BICAT3-Venus	pART7-Venus	*BICAT3* CDS	PEO1613+ PEO1425	*XmaI*
pART7-BICAT3-mCherry		*BICAT3* CDS	PEO1613+ PEO1425	*XmaI*
pBI101-PrBICAT3	pBI101	*BICAT3* promoter	PEO1642+ PEO1474	*XmaI*
pRS416-BICAT3	pRS416	*BICAT3* CDS	PEO1423+ PEO1424	*BamHI/ SalI*

Supplemental Table S3. Media used in this study.

Self-made ½ MS medium

Compound	Concentration
Sucrose	0.25 % (m/v)
Agar	0.8 % (m/v)
$MgSO_4*7H_2O$	0.75 mM
KH_2PO_4	0.62 mM
NH_4NO_3	10.31 mM
KNO_3	9.4 mM
MES (pH 5.8)	10 mM
$CoCl_2*6H_2O$	0.05 µM
$CuSO_4*5H_2O$	0.05 µM
H_3BO_3	50.14 µM
KI	2.5 µM
$Na_2MoSO_4*2H_2O$	0.52 µM
$ZnSO_4*4H_2O$	18.42 µM
Fe-EDTA	50 µM
$MnSO_4*H_2O$	37.88 µM
$CaCl_2*2H_2O$	1.5 mM

Hydroponic medium for Arabidopsis

Compound	Concentration
NH_4SO_4	0.5 mM
KNO_3	2 mM
$MgSO_4*7H_2O$	0.5 mM
KH_2PO_4	0.3125 mM
Fe-EDTA	42.5 µM
$CuSO_4*5H_2O$	0.125 µM
$ZnSO_4*4H_2O$	0.25 µM
H_3BO_3	17.5 µM
$NaMoO_4$	0.05 µM
$CoCl_2 * 6H_2O$	0.0025 µM
$MnSO_4*H_2O$	3.5 µM
$CaCl_2*2H_2O$	2 mM
adjust pH to 5.8 with KOH	
